# Whole transcriptional analysis identifies markers of B, T and plasma cell signaling pathways in the mesenteric adipose tissue associated with Crohn’s disease

**DOI:** 10.1186/s12967-020-02220-3

**Published:** 2020-01-30

**Authors:** Francesca Aparecida Ramos da Silva, Lívia Bitencourt Pascoal, Isabella Dotti, Maria de Lourdes Setsuko Ayrizono, Daniel Aguilar, Bruno Lima Rodrigues, Montserrat Arroyes, Elena Ferrer-Picon, Marciane Milanski, Lício Augusto Velloso, João José Fagundes, Azucena Salas, Raquel Franco Leal

**Affiliations:** 1 IBD Research Laboratory, Colorectal Surgery Unit, Department of Surgery, School of Medical Sciences, University of Campinas (UNICAMP), Carlos Chagas Street, 420, Cidade Universitária Zeferino Vaz, Campinas, São Paulo 13083-878 Brazil; 2 Department of Gastroenterology, IDIBAPS, Hospital Clínic, Barcelona, Spain; 3 Biomedical Research Networking Center in Hepatic and Digestive Diseases (CIBEREHD), Barcelona, Spain; 4 Laboratory of Metabolic Disorders, School of Applied Sciences, University of Campinas (UNICAMP), Limeira, São Paulo Brazil; 5 Laboratory of Cell Signaling, School of Medical Sciences, University of Campinas (UNICAMP), Campinas, São Paulo Brazil

**Keywords:** Crohn’s disease, Inflammatory bowel disease, Mesenteric adipose tissue, Transcriptomics, Immunohistochemistry

## Abstract

**Background:**

Crohn’s disease (CD) is a multifactorial disease characterized by chronic intestinal inflammation. The increased visceral adiposity near the affected intestinal area, of which mesenteric adipose tissue (MAT) is the main component, is a feature of CD. Both protective and pathological roles have been attributed to this disease-associated tissue in CD. To understand the contribution of MAT to CD pathophysiology, a molecular and cellular signature of disease-associated MAT in CD patients was provided.

**Methods:**

We performed an observational study with whole transcriptional analysis by RNA sequencing (RNA-seq) of MAT and ileal mucosa from CD patients with active disease and controls. qPCR and immunohistology were performed for validation analysis.

**Results:**

RNA-seq identified 17 significantly regulated genes (|FC| > 1.5; FDR < 0.05) in CD-MAT compared to non-IBD controls, with a marked upregulation of plasma cell genes (i.e., IGLL5, MZB1, CD79A, POU2AF1, FCRL5, JCHAIN, DERL3, SDC1, PIM2). A less strict statistical cutoff value (|FC| > 1.5, nominal p ≤ 0.05) yielded a larger list of 651 genes in CD-MAT compared to controls. CD ileum showed the significant regulation compared to control ileum of 849 genes (|FC| > 1.5; FDR < 0.05) or 2654 genes (|FC| > 1.5, nominal p ≤ 0.05). Ingenuity Pathway Analysis revealed the significant regulation of pathways related to T- and B cell functionality in the MAT of CD patients. Despite the differences between the MAT and ileal signatures of CD patients, we identified a subset of 204 genes significantly modulated in both tissues compared to controls. This common signature included genes related to the plasma cell signature. Genes such as S100A8, S100A9 (calprotectin) and IL1B, which are associated with acute inflammatory response, were exclusively regulated in the ileal mucosa of CD disease. In contrast, some genes encoding for lymphocyte receptors such as MS4A1, CD3D and CD79A were exclusively regulated in CD-MAT, exhibiting a different pattern of immune cell activation compared to the ileal mucosa in CD patients. qPCR and immunohistology confirmed the presence of large infiltrates of CD3^+^ CD20^+^ lymphocytes and CD138^+^ plasma cells in CD-MAT.

**Conclusion:**

Our data strongly supports the role of CD-associated MAT as a site for T-, B- and plasma cell activation, and suggests that it could also act as a reservoir of memory immune responses.

## Background

Crohn’s disease (CD) is a multifactorial disease characterized by chronic intestinal inflammation. Increased mesenteric adipose tissue (MAT) around the intestinal affected area is a feature of the disease. Fat wrapping and mesenteric thickening are unique to CD, as described by Crohn himself in 1932 [1]. Increasing evidence supports a link between the development of mesenteric and intestinal abnormalities in CD [2–4]. Interestingly, the transition zone between normal to fat wrapping, corresponds to the mucosal transition zone as well, and most of the mucosal ulceration is almost always confined to the mesenteric margin of the intestine, suggesting the involvement of this tissue in the pathophysiology of the disease [5–7]. Moreover, emerging radiological data suggest that mesenteric processes in CD may occur earlier than previously thought. Alterations in the mesentery, observed via magnetic resonance imaging, could occur before ulcers in the mucosa can be observed by colonoscopy [8–10]. Taken together, this data suggests that the wrapping mesenteric tissue may play a role in promoting mucosal inflammation and fibrosis.

Previous studies have shown that MAT from CD patients secretes a diverse spectrum of biologically active substances known as adipokines [8–12]. These substances exhibit endocrine or paracrine functions and are important in regulating local and systemic homeostasis [13–15]. A study by our group showed that the MAT of CD patients exhibits increased STAT1 activation, while the intestinal mucosa shows increased NF-κB activation, demonstrating tissue-specific differences as regards nuclear transcription factor activation [16]. In a recent study, Han et al. has shown in a murine model of infection that mesentery fat may constitute a reservoir of memory immune cells [17]. Another study analyzed the T-cell compartment in the MAT and intestinal mucosa of CD patients, and reported a distinct pattern of T-cell populations between ileal and colonic CD [18]. However, no studies to date have compared the cellular composition and overall signature of the MAT associated with CD versus that of non-inflammatory bowel disease (non-IBD) controls. In this study we performed whole transcriptional analysis to elucidate the molecular and cellular composition of this tissue to better understand its potential role in CD pathogenesis.

## Methods

### Patient and sample selection

Samples from the MAT and ileal mucosa located in the affected intestinal area were taken from 26 patients with ileocecal CD (CD Group) after surgical resection. Patients were included in the study after having signed written informed consent form from November 2014 to October 2017. All subjects were recruited at the Clinical Hospital of the University of Campinas (Unicamp). The control group (CTR) of MAT was composed of samples from 17 patients who underwent intestinal resection of the left colon for non-inflammatory disease [rectal cancer (n = 12), rectal prolapse (n = 3) or large polyps (n = 2)] with normal distal ileum. From these subjects MAT samples near the ileum were collected during surgery. The CTR group of ileal mucosa included ileal biopsy samples collected from 12 patients with no history of IBD who underwent ileocolonoscopy for screening or diagnostic purposes and who presented no endoscopic or histologic abnormalities. Six to eight mucosal samples of 3 mm from each surgical resected specimen were collected, using endoscopic punch forceps which was the same methodology applied to the controls of ileal mucosa biopsy collected during ileocolonoscopy. The mucosal samples were collected from the affected mucosal area of the same bowel segment from which creeping fat was obtained. Mucosal biopsies were taken from the margin of the ulcers, not from inside the ulcers to avoid necrotic areas and fibrin deposits. The samples were stored at − 80 °C immediately (15 min) after surgical resection while still at the operating room. All patients included in the study were on anti-TNF therapy and were operated upon because all failed secondarily to respond to anti-TNF treatment. None of them had been exposed to any other biologic at the time of surgery.

An observational study was performed and CD patients were grouped into two cohorts. A formal sample size calculation was not performed, because this was an exploratory study.

Table 1 shows the clinical and demographic characteristics of subjects who participated in the RNA-seq analysis and those included in the biological validation of the whole transcriptional study.

**Table 1:** Clinical and demographic characteristics of the patients included in the analysis of RNA-seq, qPCR and immunostaining

	RNA sequencing and qPCR cohort			qPCR and IH cohort		
	CD group	CTR group (MAT)	CTR group (ileal mucosa)	CD group	CTR group (MAT)	CTR group (ileal mucosa)
Number	8	4	4	18	13	8
Gender (M/F)	4/4	2/2	2/2	6/12	4/9	2/6
Age (years)	38 [20–66]	64.5 [58–67]^#^	57.5 [44–60]	36 [20–70]	56 [26–61]	57 [44–71]^#^
Disease duration (years)	6 [1–14]	–	–	6.5 [1–30]	–	–
Age at diagnosis (A1/A2/A3)^a^	1/5/2	–	–	1/14/3	–	–
Location (L1/L2/L3/L4)^a^	4/0/4/0	–	–	5/0/13/0	–	–
Behaviour (B1/B2/B3)^a^	0/3/5	–	–	0/12/6	–	–
Perianal disease (yes/no)	1/7	–	–	2/16	–	–
Immunosuppressant (yes/no)	5/3	–	–	7/11	–	–
Anti-TNFα therapy (yes/no)	8/0	–	–	18/0	–	–
CDAI	301.9 [261.4–609.6]	–	–	325.5 [162–479.8]	–	–
Presence of ulcers (yes/no)	8/0^b^	0/4^c^	0/4^c^	18/0^b^	0/13^c^	0/8^c^
Body weight (kg)	58 [48–79]	60 [52–63]	65.8 [54–72.6]	56.5 [43.4–78]	74 [49–107]	72 [58–98]
Body mass index	22.7 [15.5–26.5]	21 [20.8–21.5]	26.8 [24–29.6]	21.4 [14.7–30.4]	29.7 [21.2–36.1]	25.5 [22.7–38]

Numerical variables are described as median [min, max] and categorical variables as absolute frequencies

*CD* Crohn’s disease, *MAT* mesenteric adipose tissue, *M* male, *F* female, *TNF* tumor necrosis factor, *CDAI* Crohn’s Disease Activity Index

^#^*p* < 0.05 is considered statistically significant versus CD group

^a^Montreal classification

^b^Presence of ulcers in the ileum as evaluated by macroscopic and microscopic histological examination of the surgical specimen by a pathologist. The pathology reports by an experienced pathologist identified some degree of fibrostenotic disease in the surgical specimen together with the presence of ulcers and infiltrating immune cells in the mucosa of all patients included in the study

^c^Presence of ulcers in the ileum as evaluated by colonoscopy

### RNA extraction

Total RNA from MAT and ileal mucosa samples was extracted using the RNeasy Mini Kit (Qiagen, USA) according to the manufacturer’s instructions.

### RNA sequencing

For RNAseq analysis, the quality of total RNA was assessed using the Integrity Index (RIN) of the Agilent Bioanalyzer 2100^®^ apparatus (Agilent Technologies, Inc., Santa Clara, Calif., USA) prior to depletion of Ribosomal RNA (rRNA). Only RNA samples with an RIN equal to or greater than 7 were included in the analysis. RNA concentrations were determined by a Qubit RNA BR Assay Kit (Invitrogen) according to the manufacturer’s instructions.

cDNA libraries were prepared from 1 µg of total RNA according to the manufacturer’s instructions. RNA fragmentation, synthesis of the first and second cDNA strands, repair of cDNA strands, adenylation, indexing and fixation of adapters were performed with a TruSeq RNA Library Prep Kit v2 (Illumina Inc., San Diego, CA, USA) converted into libraries of cDNA using Illumina TruSeq Stranded mRNA. The samples were then sequenced on an HISeq^®^ 2500 platform. Calibration control and raw data quality analysis were performed via the Cutadapt v. 1.7.1.

RNA-seq generated a total of 778,614,568 reads with 100 bp and with 98.93% bases above Q30. An average of 32,442,274 million reads was produced for each sample. The sequences were aligned with the STAR Program aligner v.2.5.2a (Ensembl gtf annotation file-release GRCh38.10), thus allowing analysis of the sequences against the human reference genome and its identification. The mean sequence of alignment rate was 92.49%. Details on the performance of the mapping are provided as Additional file 1. Gene expression was quantified using the RSEM tool v.1.2.31 [19], generating a matrix of expected counts for 23,686 genes. Gene expression levels from ileal mucosa samples and MAT samples were independently normalized using the *voom* function from Linear Models for Microarray Analysis (LIMMA) v.3.34.5 [20] in R [21]. Genes with low expression levels and low expression variation across all samples from each group were removed [22, 23], resulting in 10,084 usable genes for mucosa samples and 9086 for MAT samples (see Additional files 2, 3, 4, 5). Differential expression analysis was carried out with LIMMA. P-values were adjusted for multiple testing using the Benjamini–Hochberg method [24].

### Analysis of pathways and biological processes

Analysis of significantly regulated biological pathways and processes was performed using the Ingenuity Pathway Analysis (IPA) Software (QIAGEN Inc., https://www.qiagenbioinformatics.com/products/ingenuity-pathway-analysis). The list of canonical pathways regulated and their statistical significance for each comparison was obtained. Canonical pathways were represented using polar plot representations of the pathways and their the − log(p-value) and were generated using the plotrix R package [25].

Network analysis through IPA provided the graphical representation of networks of genes commonly regulated. Genes are represented as nodes and the biological relationship between two nodes as an edge (line). Red and green nodes represent genes positively and negatively regulated genes.

### cDNA synthesis and quantitative real-time PCR (qPCR)

For qPCR analysis, RNA purity and concentration were determined by UV spectrophotometry at 260 nm using the BioTek Eon Microplate Spectrophotometer and Gen5 v 2.0 software. For cDNA synthesis, the High Capacity cDNA Reverse Transcription Kit (Applied Biosystems, Foster City, CA, USA) was used according to the manufacturer’s instructions. qPCR reactions were performed using the TaqMan™ system (Applied Biosystems). Primers consisted of the following: CD79A (HS00998119), MS4A1 (HS00544819), CTLA4 (Hs00175480), CD3D (Hs00174158), S100A8 (Hs00374264), IL1B (Hs_01555410) and GAPDH. qPCR was performed with the StepOnePlus System (Applied Biosystems) using the TaqMan Fast Advanced master mix (Life Technologies). All measurements were normalized by the expression of the GAPDH gene using the delta-delta Ct method.

### Tissue staining

Paraffin-embedded MAT blocks of 7 CD patients and 7 controls from the biological validation cohort were used for staining assays.

For histological analysis, 5 µm-thick sections were stained with H&E dye (Sigma-Aldrich).

For immunostaining, 2 µm-thick sections were pre-treated for de-paraffinization, rehydration, and epitope retrieval using EnVision FLEX Target Retrieval Solution, Low pH (Dako, Carpinteria, CA) in conjunction with PT Link (Dako). A warming step of 20 min at 95 °C was used. Sections were blocked for 30 min with 1% BSA or with animal-free blocking solution (Vector Laboratories) (only when incubated with anti-CD138 antibody). Samples were incubated overnight at 4 °C using the following commercially available antibodies: anti-CD45 (1:50; BD Pharmingen, San Jose, CA), anti-CD20 (1:100; Dako), anti-CD3 (1:200; Abcam, Cambridge, UK), anti-CD138 (1:200; R&D Systems, Minneapolis, MN) and anti-IgG (1:2000; Dako).

For immunohistochemical staining, signal detection was determined using the immunoperoxidase detection system (Vector Laboratories), followed by incubation with DAB solution (Dako). Slides were mounted with Mounting Medium (Dako). For dual immunofluorescence, biotinylated anti-mouse (1:200; Vector Laboratories) followed by incubation with streptavidin AlexaFluor 555 (1:1000; Invitrogen, Carlsbad, CA) to amplify CD20 signal, and anti-rabbit AlexaFluor 488 (1:500; Jackson Immunoresearch, West Grove, PA) were used. An incubation step with 0.1% Sudan Black (Sigma-Aldrich) for 20 min at room temperature was included to reduce tissue autofluorescence. Sections were then mounted with Vectashield Mounting Medium with DAPI (Vector Laboratories).

Photomicrographs were recorded using the Nikon Eclipse Ti S microscope with control software (NIS_Elements BR) or using Zeiss Axioplan 2 microscope with digital camera (Olympus DP–72) and control software (Cellsens).

### Statistical analysis

The results of qPCR were reported as median with interquartile ranges. To test for distributional adequacy, the Kolmogorov–Smirnov test was used to investigate if the data followed a normal distribution or a Gaussian distribution (p > 0.1). Data were analyzed using the non-parametric Mann–Whitney Test. The level of significance was set at p < 0.05.

## Results

### RNA sequencing identified a distinct signature in the mesenteric adipose tissue of Crohn’s disease patients

RNA-seq of 16 samples (MAT and ileal mucosa) from 8 CD patients and 8 non-IBD controls was performed. A comparative analysis of differentially expressed genes in CD-associated versus control MAT identified 17 genes that were differentially expressed to a significant degree (|FC| > 1.5; FDR < 0.05). Most of these genes were related to plasma cell function (IGLL5, MZB1, CD79A, FCRL5, JCHAIN, DERL3, MEI1, PIM2, MIR650 and SDC1) (Table 2).

**Table 2:** List of top genes modulated in the mesenteric adipose tissue of Crohn’s disease

	Fold change CD vs. CTR	Adjusted p value	Gene name
MIR650^a^	23.45	0.01	microRNA 650
FAM30A^a^	22.50	0.01	Family with sequence similarity 30 member A
IGLL5^a^	17.95	0.01	Immunoglobulin lambda-like polypeptide 5
MZB1^a^	17.06	0.01	Marginal zone B and B1 cell-specific protein
CD79A^a^	14.64	0.01	CD79a molecule, immunoglobulin-associated alpha
POU2AF1^a^	13.99	0.02	POU class 2 associating factor 1
FCRL5^a^	13.28	0.04	Fc receptor-like 5
JCHAIN^a^	12.03	0.01	Joining chain of multimeric IgA and IgM
DERL3^a^	8.35	0.01	Derlin 3
CYP4F35P	7.90	0.01	Cytochrome P450, family 4, subfamily F, polypeptide 35, pseudogene
SDC1^a^	7.54	0.04	syndecan 1 (CD138)
ANKRD36BP2	7.09	0.01	Ankyrin repeat domain 36B pseudogene 2
CCL11	5.67	0.05	Chemokine (C-C motif) ligand 11
PIM2^a^	4.52	0.04	Pim-2 oncogene
MEI1^a^	4.26	0.05	Meiosis inhibitor 1
FAM46C	3.86	0.05	Family with sequence similarity 46, member C
PAPPA	− 4.31	0.01	Pregnancy-associated plasma protein A, pappalysin 1

*CD* Crohn’s disease, *CTR* control

^a^Encoding genes of plasma cell function

**Fig. 1:**
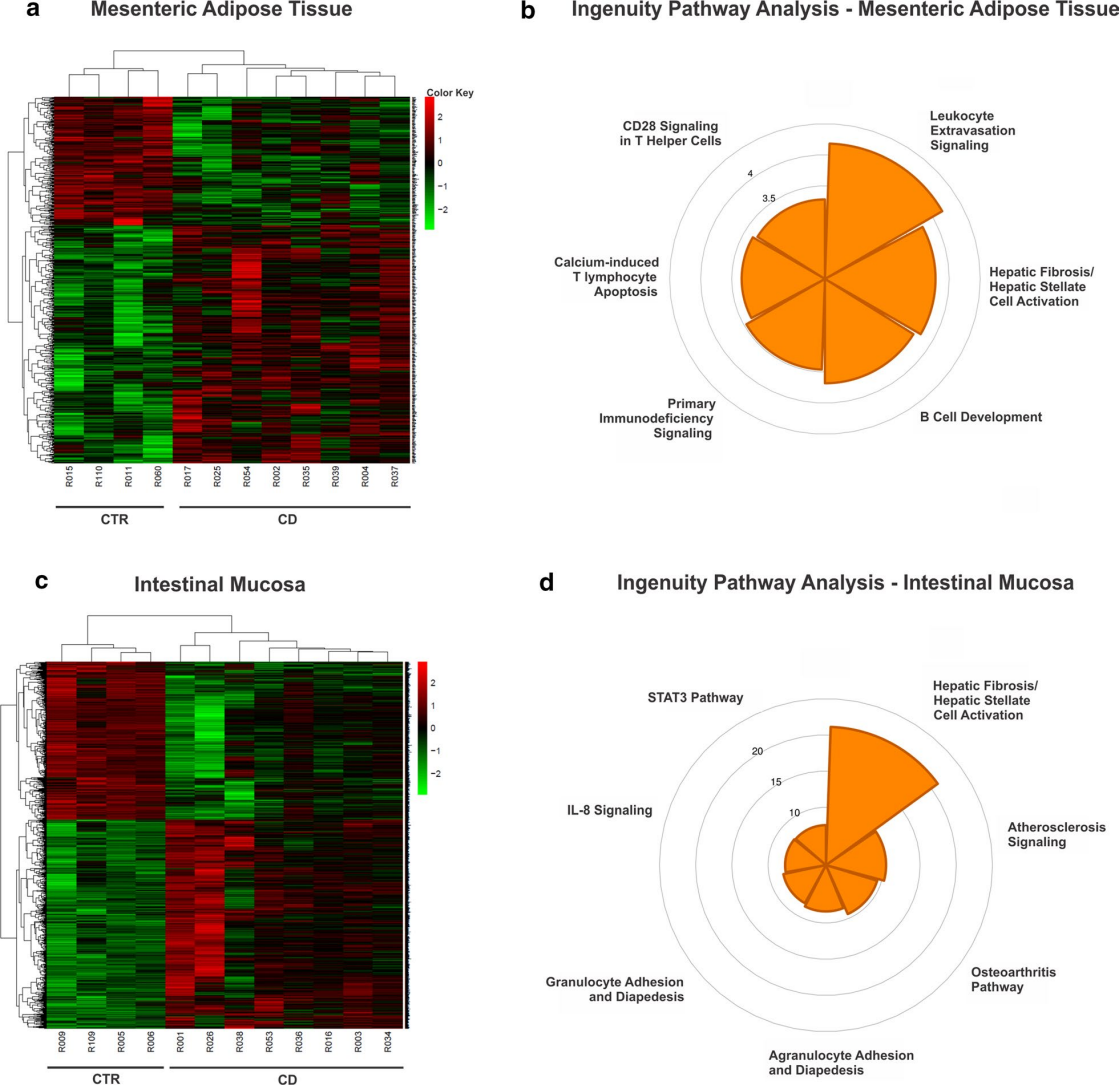
Heat map representation and pathway analysis of the differentially expressed genes in the mesenteric adipose tissue (MAT) and ileal mucosa of Crohn’s disease (CD) compared to the respective control (CTR) groups based on RNA sequencing. For the CD group, n = 8. For the CTR group, n = 4. **a**, **c** Each line represents one individual gene, and each column an experimental sample. Differentially upregulated genes are shown in red and downregulated genes are shown in green. Based on the number of sequences identified by each gene in the CTR and CD groups, it was possible to quantify the expressed genes using the RSEM tool v.1.2.31. **b** Canonical pathways significantly regulated genes in the MAT of CD patients are shown in a polar plot representing the − log(p-value) of each pathway association. The analysis was based on Ingenuity Pathway Analysis (IPA) Software (QIAGEN Inc., https://www.qiagenbioinformatics.com/products/ingenuity-pathway-analysis). **d** Polar plots representing the most significantly regulated canonical pathways in the ileal mucosa of CD patients. The analysis was based on Ingenuity Pathway Analysis (IPA). The − log(p-value) of each pathway association is shown

Analysis using a less strict statistical cutoff value (|FC| > 1.5, nominal p ≤ 0.05) revealed a larger list of genes: 425 genes upregulated and 226 genes downregulated in CD-associated adipose tissue compared to controls. Figure 1a shows a heatmap representation of these 651 differentially expressed genes (Additional file 6). Network analysis (IPA) revealed a significant regulation of canonical pathways associated with different cellular functions, primarily related to T- and B-cell subsets (Fig. 1b).

We next compared the transcriptional profiles of ileal mucosa in CD and non-IBD controls and identified 849 significantly regulated genes between the two groups (|FC| > 1.5; FDR < 0.05). Using the same, less strict, cutoff criteria described above (nominal p ≤ 0.05), we detected 2654 differentially expressed genes, including 1513 upregulated genes and 1141 downregulated genes (Additional file 7). The heatmap of these differentially expressed genes in the ileal mucosa is represented in Fig. 1c, and their network analysis (IPA) in Fig. 1d.

### An increase in plasma cell signatures was observed in the ileum and adjacent mesenteric adipose tissue of Crohn’s disease patients

**Fig. 2:**
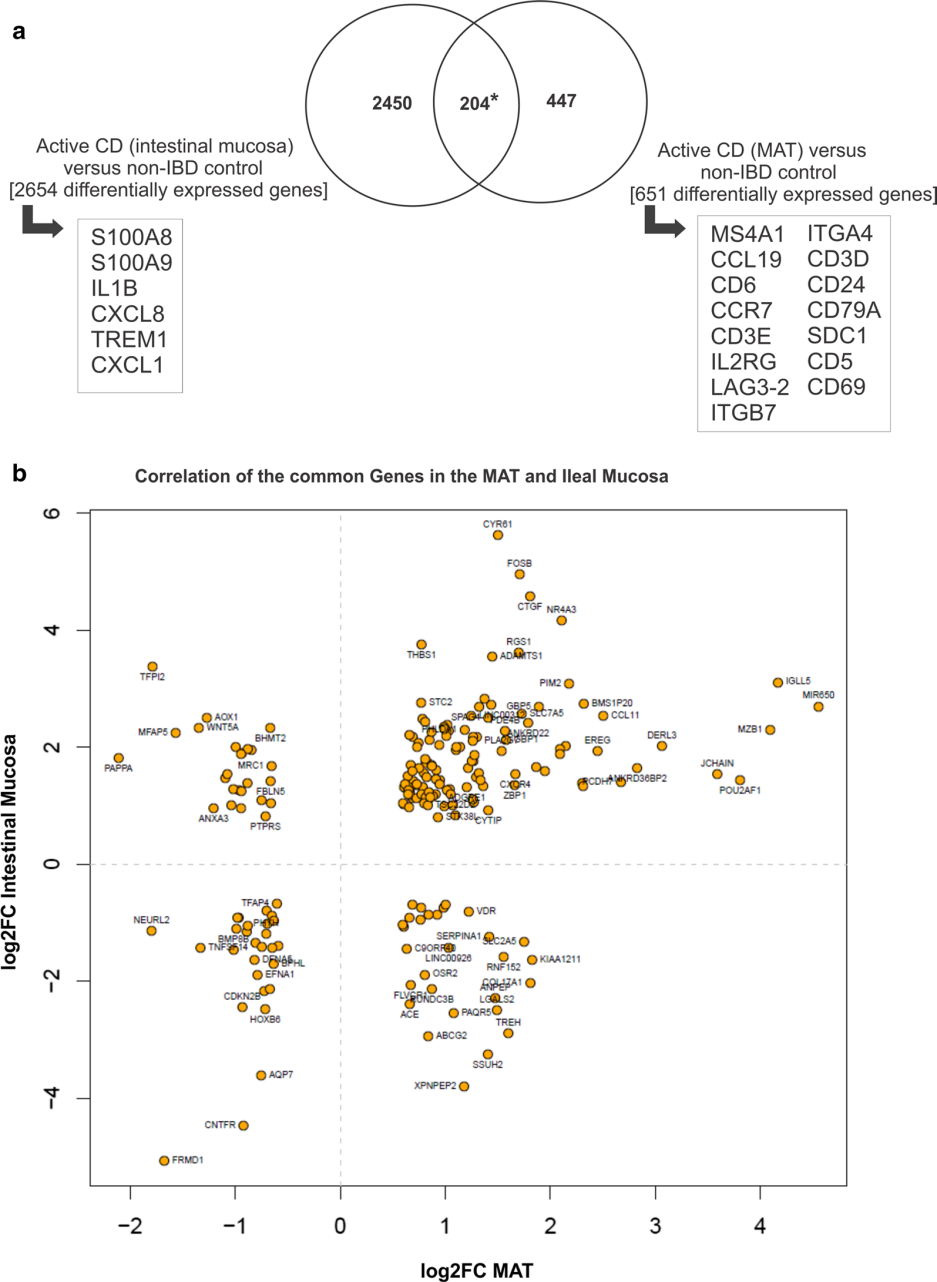
Plasma cell signature in the ileal mucosa and mesenteric adipose tissue (MAT) of Crohn’s disease (CD) patients. **a** Venn Diagram showing the intersection* of differentially expressed genes in MAT and ileal mucosa of CD patients versus controls with non-inflammatory bowel disease (non-IBD). **b** Correlation of the common genes in the MAT and ileal mucosa in CD patients. The logarithm of the fold change (log2FC) of significantly regulated genes in CD-ileum compared to CTRL-ileum is plotted against log2FC in CD-MAT compared to CTRL-MAT

We then compared the genes that were differentially regulated both in the MAT and ileum of CD patients relative to controls and identified a group of 204 genes that were common to both signatures (Fig. 2a). Figure 2b shows the fold change (FC) for each gene in the MAT and ileum of CD compared to controls. Our results indicate that the highest upregulated genes in CD-associated MAT (including plasma cell-related DERL3, MZB1, JCHAIN, IGLL5, POU2AF1, MIR650 and PIM2) were also significantly upregulated in the adjacent ileal mucosa. In contrast, some genes (i.e. TFPI2, AOX1, WNT5A, PAPPA, XPNPEP2, SSUH2, ABCG2, etc.) showed an inverse FC correlation between the ileum and MAT. While some of these genes are primarily expressed by stromal cells, including fibroblasts and epithelial cells, pathway analysis did not provide any further information (data not shown).

In addition to the 204 genes regulated in the MAT and ileum, the majority of the signatures remained exclusively and differentially modulated in each of the tissues. Remarkably, genes that were highly upregulated in CD ileal mucosa, such as S100A8 and S100A9 (calprotectin), IL1B, CXCL8, TREM1 and CXCL1, which are associated with acute pro-inflammatory response pathways, were not differentially expressed in the adjacent MAT. Likewise, several genes encoding for surface proteins expressed on T and B cells pathways—such as MS4A1(CD20), CD6, CD3D, CD3E, IL2RG, LAG3-2, CD24, CD79A, CD5, CD69 and related to leukocyte extravasation signaling pathway, such as the integrins ITGB7 and ITGA4—were exclusively regulated in CD-MAT (Figs. 1b, d and 2a). Interestingly, we did not find a consistent set of M2 macrophage phenotype marker in our data; only CD163 (log2FC = − 0.95, p = 0.0085) was differently expressed in the MAT of CD patients compared to the control group (Additional file 6). Other markers such as, SR, MMR/CD206, CD200R, TGM2, DecoyR, IL-1RII, CD86, TLR1, TLR8 and VEGF were not modulated in the RNA seq data.

### Biological validation of RNA-seq results

To confirm our results, we utilized qPCR analysis to validate the expression of a number of representative genes in an additional group of MAT samples (n = 26 for the CD group and n = 17 for the non-IBD control group).

**Fig. 3:**
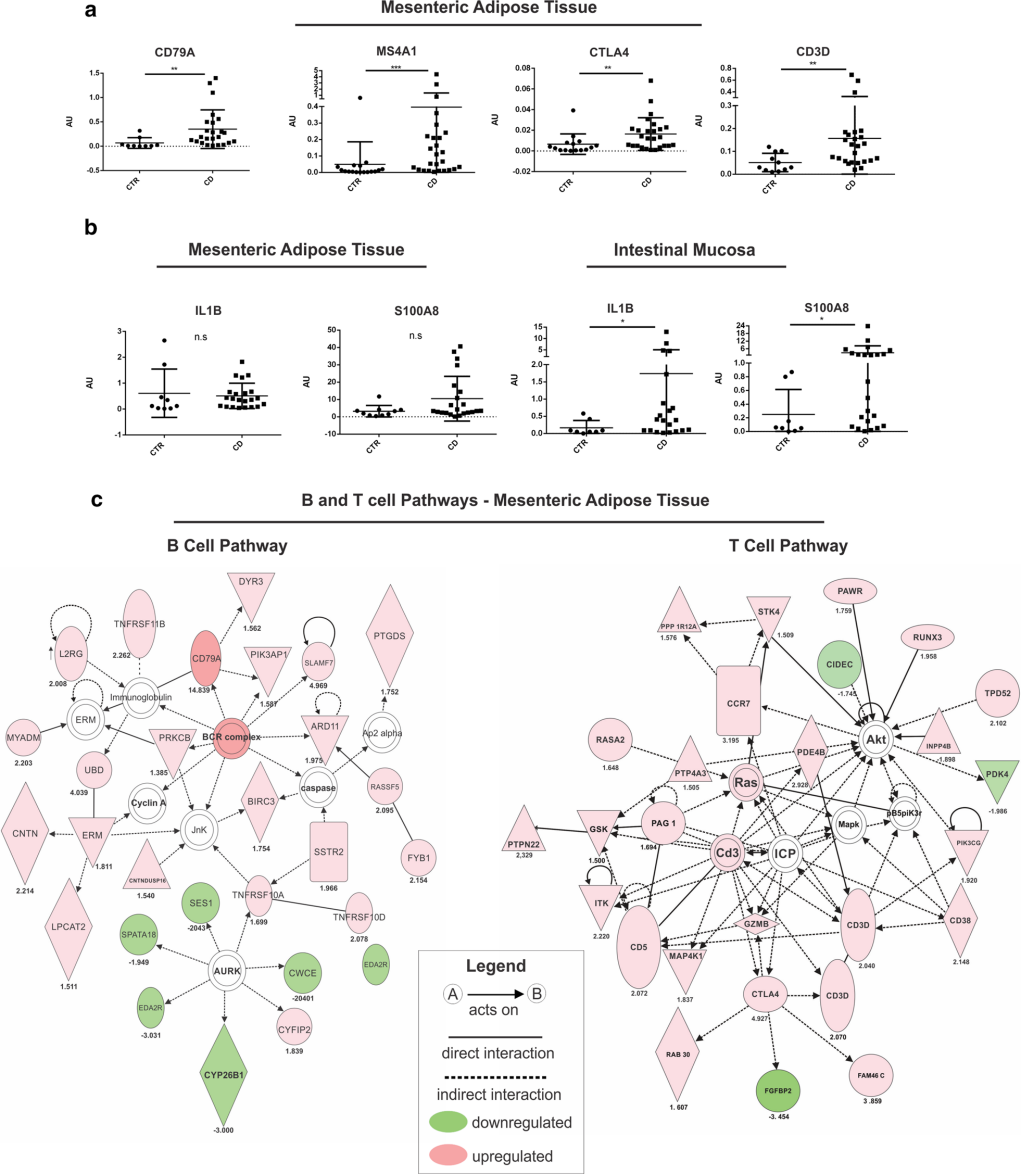
Transcriptional expression of CD79, MS4A1, CTLA4 and CD3D in the mesenteric adipose tissue (MAT) of Crohn’s disease (CD) patients. **a** mRNA levels (qRT-PCR) of CD79, MS4A1, CTLA4 and CD3D were investigated in the MAT of CD patients (CD group) compared to controls (CTR group). All these genes were differentially expressed in the MAT of CD compared to the control, identified by RNA sequencing. **b** Transcriptional levels of pro-inflammatory markers in the MAT and ileal mucosa of CD patients (CD group) and respective controls (CTR group). For CD, *n* = 26; for CTR (MAT), *n*=17, for CTR (ileum), n = 12, ∗*p* < 0.05 is considered statistically significant versus control group. **c** Network analysis was performed by Ingenuity Pathway Analysis (IPA) of the differentially expressed genes by RNA sequencing. The networks represent the molecular relationships between genes and genes products that are present in CD-MAT and that belong to B cell or T cell pathways

The selected genes included the following: CD79A (FC = 14.64), which is essential for signaling through the B-cell receptor; MS4A1 (FC = 11.29), a gene encoding the CD20 involved in the regulation of B-cell activation and proliferation; and CTLA4 (FC = 4.93), a receptor upregulated upon T-cell activation and an important inhibitor of T-cell function. Moreover, the TCR complex proteins CD3D was also significantly overexpressed in CD MAT (FC = 2.07). PCR analysis confirmed the increased expression of CD79A (p = 0.0007), MS4A1 (p = 0.0005), CTLA4 (p < 0.0001) and CD3D (p = 0.0048) in CD compared to control MAT (Fig. 3a).

Furthermore, we measured the expression of IL1B (FC = 9.31) and S100A8 (FC = 21.04), two genes that we found to be significantly upregulated by RNAseq in CD ileal mucosa compared to controls, though not in CD-MAT. In this larger data set PCR analysis confirmed the significant regulation of both IL1B and S100A8 in CD-ileum, but not in CD-MAT (Fig. 3b). Figure 3c illustrates the network interactions between some of the genes significantly regulated by RNAseq in the adipose tissue of CD patients (Fig. 3c).

### Crohn’s disease associated-mesenteric adipose tissue showed an increase in B, T and plasma cell infiltration

**Fig. 4:**
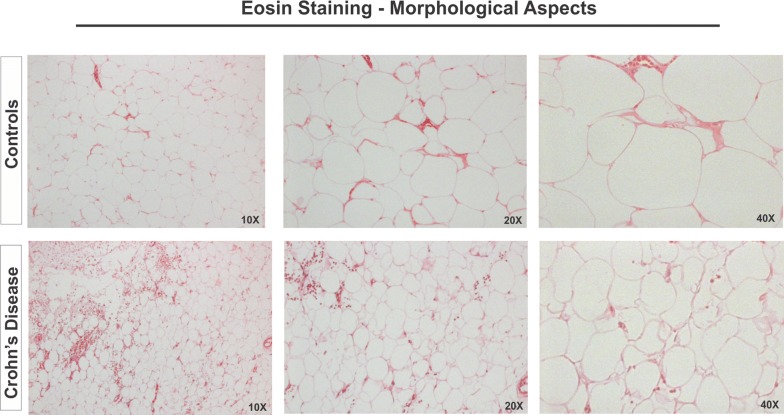
Structural histological analysis of mesenteric adipose tissue (MAT) from Crohn’s disease (CD) patients. Eosin staining was performed on paraffin-embedded slides from the MAT of Crohn’s disease (CD) patients and controls (CTR). 10×, 20× and 40× objective lenses were used as indicated in each image

Finally, we confirmed our transcriptional findings using immunostaining of paraffin-fixed tissues. Eosin staining of MAT showed a reduced adipocyte area and perimeter in CD patients compared to controls, in agreement with the published literature [26] (Fig. 4).

**Fig. 5:**
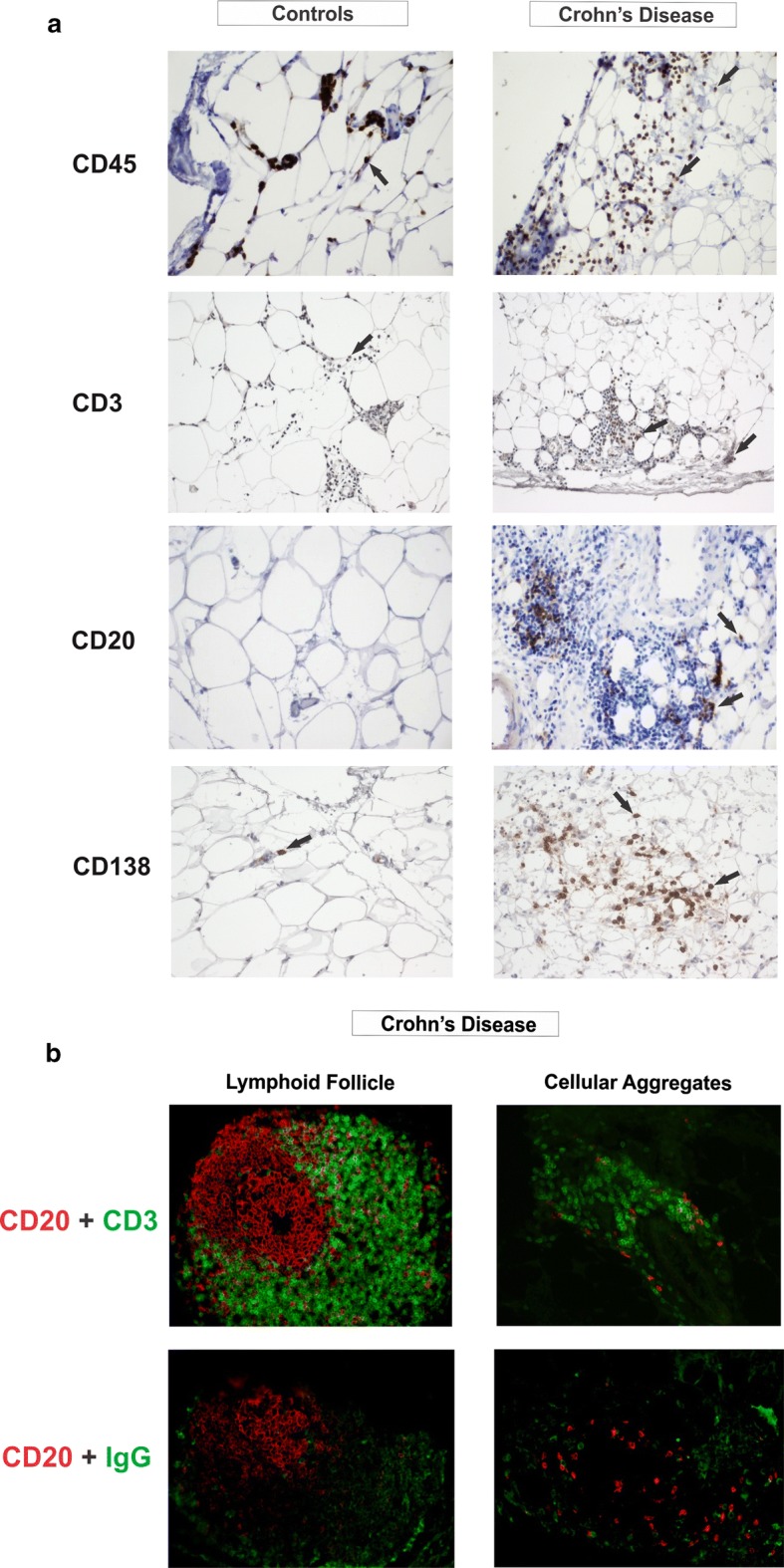
Immunostaining of Crohn’s disease mesenteric adipose tissue (MAT) showed an increase in B, T and plasma cell infiltration. **a** Immunohistochemical analysis of CD45, CD20, CD3 and CD138 were performed on paraffin-embedded slides from the MAT of CD and CTR groups. The arrows show cells positive to the staining. 20× objective lens was used. **b** Double-immunofluorescent staining of CD20 with CD3 or IgG was performed on paraffin-embedded slides from the MAT of CD and CTR groups. Positive cells for CD20 are shown in red, while positive cells for CD3 and IgG are shown in green. 20× objective lens was used

The staining of CD45, a marker of most hematopoietic cells (except for plasma cells, which are known to down-modulate its expression) confirmed the presence of abundant infiltrating immune cells in the MAT from CD. CD3 immunoreactivity identified all T lymphocytes, while CD20 was used as a marker expressed by naïve and memory B cells and CD138 was used as a marker of plasma cells. In agreement with the transcriptional data, a marked increase in CD3, CD20 and CD138 positive cells forming abundant cellular aggregates were noticed in the MAT of CD patients, while small aggregates of isolated hematopoietic cells were seen in some areas of the MAT of healthy controls (Fig. 5a). Immunofluorescence analysis also confirmed the presence of B and T cells, as well as of B cells and plasma cells (identified by IgG) in CD-MAT in both follicular structures and large cell aggregates (Fig. 5b).

## Discussion

Altered MAT near the affected intestinal area is considered to be characteristic of severe CD [6, 27–30]. Persistent alterations in this tissue even after endoscopic remission can be observed by magnetic resonance imaging [31], and may be associated with a complicated disease course and abdominal surgery in CD [32]. There is now more evidence to link intestinal and mesenteric abnormalities in CD, and this knowledge may influence clinical and surgical management of this disease. An example is the study by Groff et al. [33], which reported more perineal complications in those CD patients who had a close rectal dissection, thus leaving the mesorectum in the pelvis, when compared to total mesorectal dissection (59.5% versus 17.6%), which resulted in lower healing rates. Another study showed the association of mesenteric lymphatic density with disease behavior and postoperative recurrence [34]. Yet another group concluded that the inclusion of mesentery in ileocolic resections for CD reduces recurrences requiring follow-on surgery [35]. However, these results must be heeded with caution and larger cohorts of patients are needed to validate them.

Consistent with other studies [5, 16, 26], we also found morphological differences in the MAT of CD patients compared to controls. These alterations, such as reduced adipocyte area and perimeter in the histologic analysis, were also present in the mesentery of severely obese patients without IBD [36]. However, CD patients from the present study were not obese, and although they presented altered MAT (creeping fat) near the affected intestinal area, seen by clinical macroscopic analysis of the surgical specimen, they had a normal or low body mass index (BMI). Mesentery fat has several components, such as differentiated mature adipocytes, an extracellular matrix with fibroblasts, microvascular pericytes (important in angiogenesis), lymphatics, nerves, mesenchymal stem cells, pre-adipocytes and immune cells [36–38]. Although histological abnormalities, such as fibrosis and perivascular and perineuronal inflammation, have already been described in MAT-associated CD, their role in the development and clinical presentation of the disease remains unclear [39].

Recently, Kredel et al. identified a different pattern of T-cell subpopulations in the MAT associated with ileal, colonic–CD and ulcerative colitis, depending on the disease location and the type of IBD [18]. However, they did not perform a comparative analysis with the MAT of control individuals, and most of the patients with colonic-CD underwent surgery due to therapy-refractory inflammation and not for stenosing disease, in which creeping fat is generally present [40]. In a previous study, they identified an increase of regulatory M2 macrophages in the MAT of CD [41]. However, in our data we did not observe a clear M2 macrophage signature, showing that the MAT in CD may not play a protective role in this affection.

Ours is the first study to employ RNA-seq analysis to evaluate genome-wide transcriptome changes of MAT in CD patients and controls. RNA-seq technology was chosen because of the advantages it provides compared to the other methods for generating global transcriptomic profiles [42].

The analysis of differentially expressed genes in the MAT and ileum of CD patients identified the distinct molecular signaling pathways activated in CD, depending on the studied tissue. Transcripts related to acute inflammatory response, including neutrophil and activated macrophage markers, were exclusively regulated in the CD ileal mucosa, whereas genes encoding for T-cell expressed proteins, such as CD3D, CD3E, MS4A1 and CTLA4, were exclusively overexpressed in the CD-MAT, showing relevant tissue-specific differences and suggesting that MAT in CD patients may constitute a memory immune response reservoir potentially implicated in the pathophysiology of the disease. On the other hand, transcripts such as S100A8, S100A9 (calprotectin) and IL1B, which are highly upregulated in the CD ileal mucosa, were not significantly increased in the CD-MAT. The S100A8 qPCR analysis (Fig. 3b) clearly showed a wide range of variation among patients. Such differences may occur because the presence of fibrostenosis concomitant to the inflammatory process in the surgical specimens, as analyzed by an experienced pathologist. However, overall we show the significantly higher transcriptional levels of S100A8 (calprotectin) in the ileal mucosa, and equal levels to the controls in the mesenteric adipose tissue, displaying tissue specific-differences.

Remarkably, only 204 genes were modulated both in the MAT and ileal mucosa of CD patients compared to their respective controls. Interestingly, there was an evident common plasma cell signature that was up-regulated (MZB1, POUZAF1, IGLL5, JCHAIM, DERL3 and PIM2) in both tissues. These data, which we validated in an independent cohort by qPCR and immunostaining, strongly supports the role of the MAT in CD as a niche for lymphocytes (T and B cell, as well as plasma cells) and a site of antigen presentation.

As has been demonstrated in other adipose depots, MAT is able to propagate metabolic and inflammatory signals systemically, potentially modulating the clinical characteristics of CD. MAT may respond to environmental stimuli and coordinate intestinal responses locally and systemically [43]. One of the responses that may be modulated by MAT is immunological. Our study suggests that CD-associated MAT mainly differs from control adipose tissue from the same location in its high content of CD3^+^ T, CD79A^+^CD20^+^B and positive antibody-producing plasma cell markers. Although we did not perform IHC of the ileal mucosa samples in this study, the literature has extensively shown that the ileal mucosa contains a large immune cell infiltrate [44]. However, the relationship with the MAT near the affected intestinal area is still a subject of investigation. Randolph et al. [45] also studied CD surgical specimens and reported the presence of CD3^+^ T cells and CD20^+^ B cells in the MAT lymph nodes, linking them to lymphatic vessel remodeling. They confirmed B cell-rich aggregates on these lymphatic collecting vessels, which enter and exit lymph nodes, known as tertiary lymphoid organs [45, 46]. In addition, the study suggests that tertiary lymphoid organs are anatomically located to assume immunological roles and to participate in altering the course of immunological communication. Thus, processes that remodel these vessels may affect communication between lymph nodes and the intestinal lamina propria [45]. However, unlike our own, this study’s investigation of the cellular composition of MAT samples from the control group involved a different anatomic than that of the CD group. The most novel finding from our study was the marked signature of antibody-producing plasma cells, which is characteristic of CD-associated MAT and which was shared by the adjacent inflamed ileal mucosa. This suggests a possible involvement of MAT not only in the maintenance of inflammation in CD, through a reservoir of immune cells, but also in antibody-producing responses. Indeed, higher antibody responses have been associated with more complicated CD, including ileal stenosing disease [47–50].

One limitation of our study was the difference concerning the median age of the patients included in the study. This occurred because patients who undergo colonoscopy examination for colorectal cancer screening and those who undergo colorectal resection for cancer are usually older than CD patients. Another limitation is the sample size. Despite the small sample size, this observational study has the benefit of interrogating thousands of genes on each samples. Future validation studies can focus on selected genes/pathways in larger cohorts. Moreover, while our study does not directly test the hypothesis that antibody production in the MAT may contribute to disease in these patients, it does certainly suggest a new role for MAT in CD pathogenesis that would be worth exploring in future studies.

## Conclusion

There are many pathways by which MAT may contribute to CD, and our study sheds new light on the role this tissue plays in storing memory immune cells and potentially supporting antigen-driven immune responses. The fact that the most distinct signature identified in CD-MAT was that of antigen-producing plasma cells points towards a potential novel implication of this tissue vis-à-vis antibody responses and warrants further investigation.

## Supplementary information

**Additional file 1.** Statistical details of the RNA sequencing analysis.

**Additional file 2.** Mapping stats (1/4). Number of reads per sample.

**Additional file 3.** Mapping stats (2/4). Basic mapping stats.

**Additional file 4.** Mapping stats (3/4). Mismatch rate per base.

**Additional file 5.** Mapping stats (4/4). Ratio of uniquely mapped reads to multi-mappers.

**Additional file 6.** List of 651 differentially expressed genes identified by RNA-Seq (p < 0.05) in the mesenteric adipose tissue of Crohn’s disease patients compared to the controls sorted by log2 fold change.

**Additional file 7.** List of 2.654 differentially expressed genes identified by RNA-Seq (p < 0.05) in the ileal mucosa of Crohn’s disease patients compared to the controls sorted by log2 fold change.

## Data Availability

All relevant data supporting the findings of this study are available within the paper. The RNA sequencing data of this study are available from the corresponding author upon reasonable request. Details of the RNA sequencing analysis and of the mapping performance are available as additional files.
