# Supplementary material for: Whole transcriptional analysis identifies markers of B, T and plasma cell signaling pathways in the mesenteric adipose tissue associated with Crohn’s disease
Source: J Transl Med. 2020 Jan 30;18:44. doi: 10.1186/s12967-020-02220-3 (PMC6993458; doi:10.1186/s12967-020-02220-3)
Supplement: Supplementary file 1 — Additional file 1. Statistical details of the RNA sequencing analysis. [file 12967_2020_2220_MOESM1_ESM.pdf]

**Additional file 1. Statistical details of the RNA sequencing analysis.**

1) Removal of genes with low expression. A gene was considered to have low expression if: (1) its expression level was 0 across all samples, or (2) its mean expression level was lower than the $10^{th}$ quantile of the distribution of mean expression levels of all genes across all samples.

2) Removal of genes with low coefficient of variation (CV). CV of gene *i* was defined as:

$$CV_i = \frac{\sigma_i}{\bar{x}_i}$$

where $x_i$ is the mean of the expression of gene *i* and $\sigma_i$ is its standard deviation. A gene *i* was removed from the analysis if $CV_i$ was lower than the $25^{th}$ quantile of the distribution of the CV of all genes across all samples.
