# Supplementary figures and images for: Whole transcriptional analysis identifies markers of B, T and plasma cell signaling pathways in the mesenteric adipose tissue associated with Crohn’s disease
Source: J Transl Med. 2020 Jan 30;18:44. doi: 10.1186/s12967-020-02220-3 (PMC6993458; doi:10.1186/s12967-020-02220-3)

**Additional file 2. Mapping stats (1/4).** Number of reads per sample.

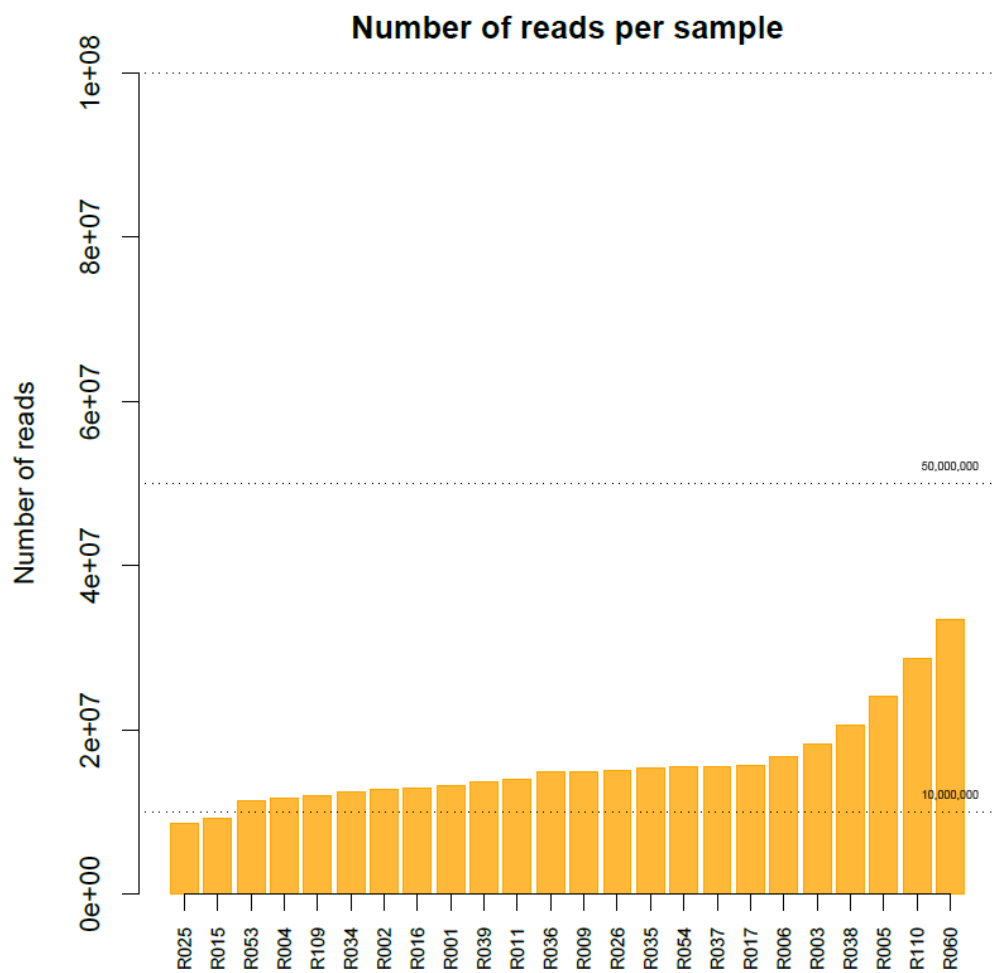

Supplement: Supplementary file 2 — Additional file 2. Mapping stats (1/4). Number of reads per sample. [file 12967_2020_2220_MOESM2_ESM.pdf]

Additional file 3. Mapping stats (2/4). Basic mapping stats.

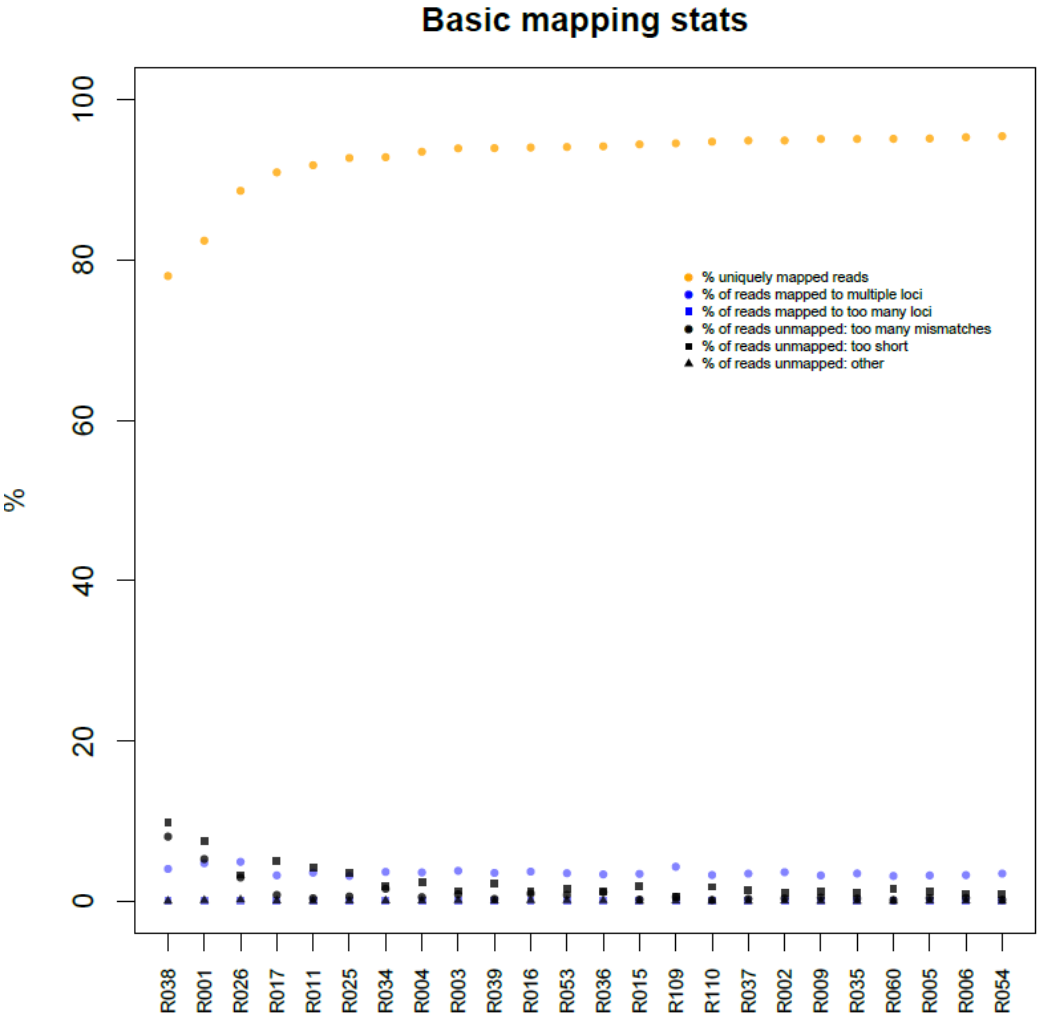

Supplement: Supplementary file 3 — Additional file 3. Mapping stats (2/4). Basic mapping stats. [file 12967_2020_2220_MOESM3_ESM.pdf]

**Additional file 4. Mapping stats (3/4).** Mismatch rate per base.

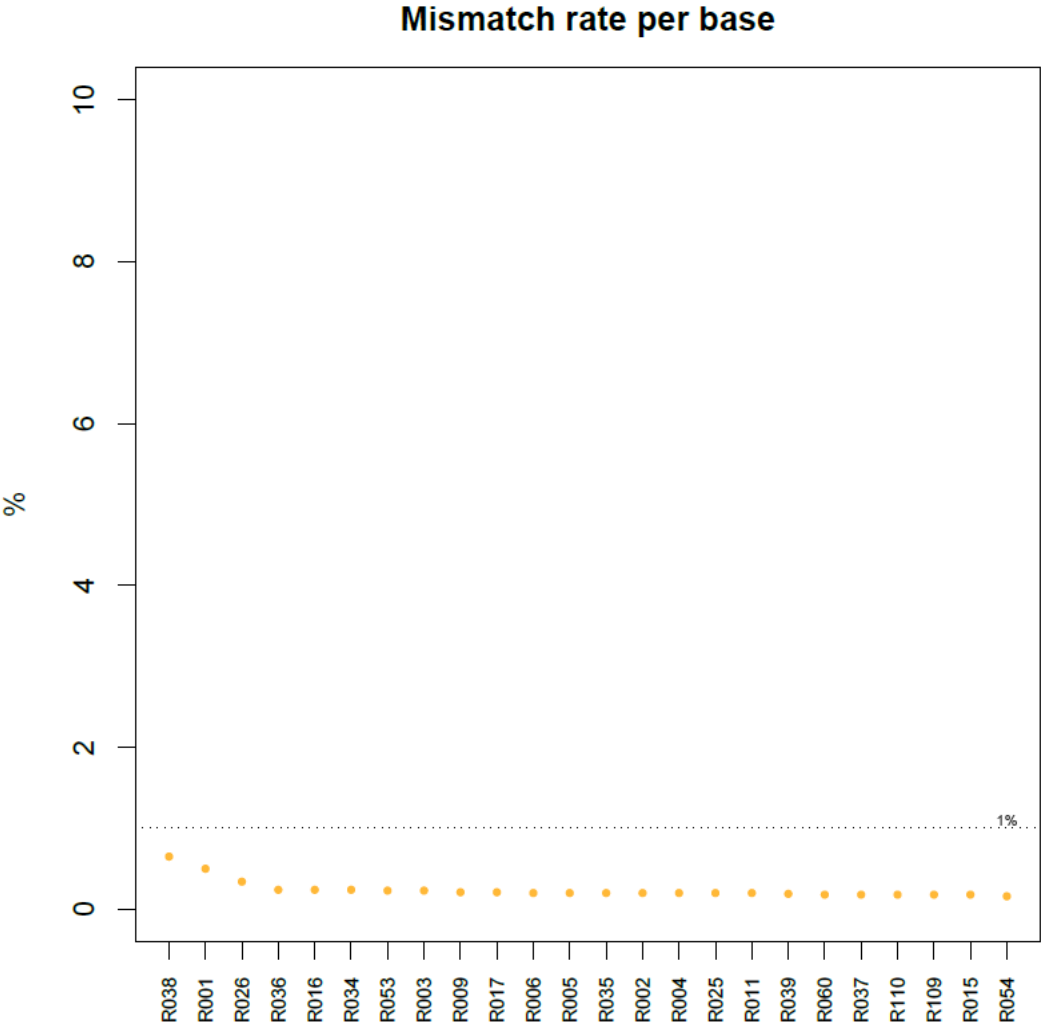

Supplement: Supplementary file 4 — Additional file 4. Mapping stats (3/4). Mismatch rate per base. [file 12967_2020_2220_MOESM4_ESM.pdf]

**Additional file 5. Mapping stats (4/4).** Ratio of uniquely mapped reads to multi-mappers.

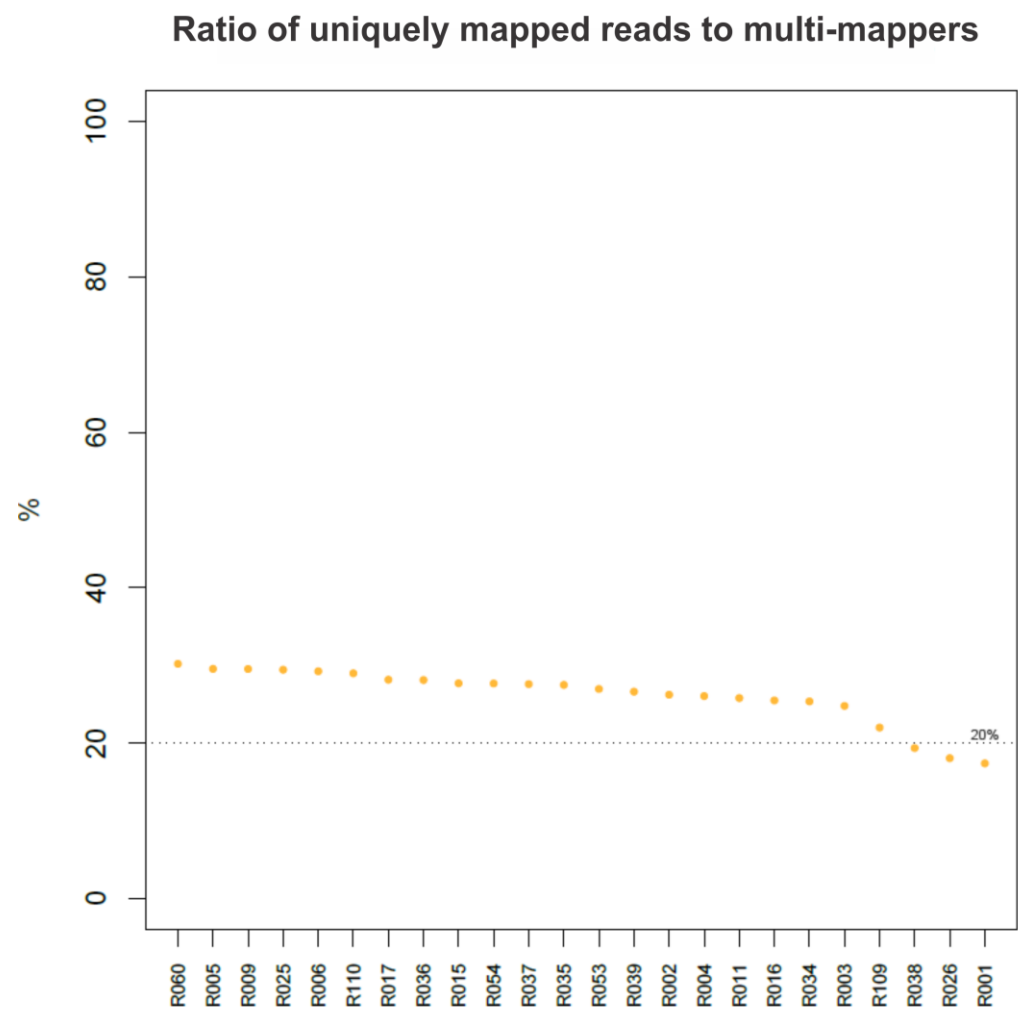

Supplement: Supplementary file 5 — Additional file 5. Mapping stats (4/4). Ratio of uniquely mapped reads to multi-mappers. [file 12967_2020_2220_MOESM5_ESM.pdf]
