# Supplementary material for: Whole transcriptional analysis identifies markers of B, T and plasma cell signaling pathways in the mesenteric adipose tissue associated with Crohn’s disease
Source: J Transl Med. 2020 Jan 30;18:44. doi: 10.1186/s12967-020-02220-3 (PMC6993458; doi:10.1186/s12967-020-02220-3)
Supplement: Supplementary file 6 — Additional file 6. List of 651 differentially expressed genes identified by RNA-Seq (p < 0.05) in the mesenteric adipose tissue of Crohn’s disease patients compared to the controls sorted by log2 fold change. [file 12967_2020_2220_MOESM6_ESM.pdf]

Additional file 6. List of 651 differentially expressed genes identified by RNA-Seq (P < 0.05) in the mesenteric adipose tissue of Crohn's disease patients compared to the controls sorted by Log2 fold change.

Gene	Log2 Fold Change	P-value	Locus
MYOC	-3.67	0.0001	1q23-q24
PKHD1L1	-2.56	0.0301	8q23
HP	-2.53	0.0054	16q22.2
MCOLN3	-2.39	0.0004	1p22.3
CETP	-2.36	0.0053	16q21
PRG4	-2.33	0.0143	1q25-q31
CYP4B1	-2.29	0.0134	1p34-p12
PI16	-2.27	0.0011	6p21.2
SERPINA3	-2.20	0.0004	14q32.1
PAPPA	-2.11	0.0000	9q33.2
CLEC4GP1	-2.01	0.0012	19p13.2
RASL10B	-1.98	0.0006	17q12
SCARA5	-1.93	0.0389	8p21.1
ALDH1A2	-1.91	0.0098	15q21.3
NEURL2	-1.80	0.0052	20q13.12
FGFBP2	-1.79	0.0240	4p16
TFPI2	-1.79	0.0137	7q22
GLYAT	-1.77	0.0482	11q12.1
FRMD1	-1.67	0.0129	6q27
EPB42	-1.65	0.0069	15q15-q21
AQP7P3	-1.63	0.0018	9p12
SGCG	-1.63	0.0002	13q12
EDA2R	-1.60	0.0054	Xq12
CYP26B1	-1.58	0.0035	2p13.2
MFAP5	-1.56	0.0011	12p13.1-p12.3
TMEM100	-1.52	0.0011	17q22
WT1	-1.50	0.0193	11p13
SPOCK1	-1.50	0.0101	5q31.2
PAMR1	-1.48	0.0464	11p13
TRHDE-AS1	-1.43	0.0015	12q21.1
PGM5-AS1	-1.38	0.0153	9q13
LOC100289230	-1.37	0.0002	5q21.1
OSR1	-1.36	0.0180	2p24.1
FGF1	-1.35	0.0323	5q31
PROCR	-1.35	0.0141	20q11.2
WNT5A	-1.35	0.0104	3p21-p14

TNFSF14	-1.33	0.0483	19p13.3
GFPT2	-1.32	0.0401	5q34-q35
ALDH1L1-AS2	-1.29	0.0108	3q21.3
C14ORF180	-1.28	0.0009	NA
AOX1	-1.27	0.0065	2q33
VWCE	-1.26	0.0091	11q12.2
LYVE1	-1.26	0.0044	11p15
ADCY2	-1.24	0.0314	5p15.3
TIMP4	-1.22	0.0277	3p25
LOC286367	-1.21	0.0202	9q31.1
ANXA3	-1.20	0.0288	4q21.21
RBPMS-AS1	-1.20	0.0197	8p12
GPX3	-1.19	0.0015	5q23
NOX4	-1.19	0.0019	11q14.2-q21
LOC100506388	-1.17	0.0109	NA
PTPRO	-1.16	0.0020	12p13-p12;12p13.3-p13.2
HOXC9	-1.15	0.0069	12q13.3
AKR1C2	-1.13	0.0167	10p15-p14
ALDH2	-1.12	0.0053	12q24.2
GSN	-1.12	0.0043	9q33
PID1	-1.11	0.0059	2q36.3
PPP1R1A	-1.11	0.0149	12q13.2
THAP7-AS1	-1.10	0.0073	22q11.21
HSD11B1	-1.10	0.0218	1q32-q41
MGC45800	-1.10	0.0236	4q34.3
MESP1	-1.10	0.0322	15q26.1
APOD	-1.09	0.0334	3q26.2-qter
APCDD1	-1.08	0.0135	18p11.22
CTTNBP2	-1.06	0.0005	7q31
C17ORF58	-1.06	0.0044	NA
SLC7A8	-1.05	0.0003	14q11.2
CALB2	-1.05	0.0256	16q22.2
HPGDS	-1.04	0.0385	4q22.3
NTRK2	-1.04	0.0097	9q22.1
CRYAB	-1.04	0.0232	11q22.3-q23.1
SESN1	-1.03	0.0001	6q21
CFD	-1.02	0.0410	19p13.3
TMEM147-AS1	-1.02	0.0023	NA
BMP8B	-1.01	0.0253	1p35-p32
FBXO27	-1.00	0.0069	19q13.2
GAPDHS	-1.00	0.0045	19q13.12
SLC1A3	-1.00	0.0341	5p13
CLEC3B	-0.99	0.0438	3p22-p21.3
CPAMD8	-0.99	0.0152	19p13.11
ZNF436-AS1	-0.99	0.0023	NA
PDK4	-0.99	0.0161	7q21.3

ALDH4A1	-0.99	0.0366	1p36
ADH1B	-0.98	0.0386	4q23
IL17D	-0.98	0.0095	13q11
ENPP1	-0.98	0.0212	6q22-q23
CORO6	-0.98	0.0035	17q11.2
KLF15	-0.98	0.0283	3q21.3
FAM13A	-0.97	0.0134	4q22.1
CLSTN2	-0.96	0.0304	3q23
SPATA18	-0.96	0.0229	4q12
HEPACAM	-0.96	0.0147	11q24.2
FCER2	-0.96	0.0127	19p13.3
PLEKHF1	-0.96	0.0067	19q12
GPBAR1	-0.95	0.0496	2q35
SHISA6	-0.95	0.0478	17p12
CD163	-0.95	0.0085	12p13.3
FDXR	-0.94	0.0046	17q25.1
LINC00899	-0.94	0.0105	22q13.31
C1QTNF1	-0.94	0.0077	17q25.3
CPM	-0.94	0.0318	12q14.3
LINC01089	-0.94	0.0032	NA
PHGDH	-0.93	0.0187	1p12
CDKN2B	-0.93	0.0206	9p21
HEBP2	-0.93	0.0155	6q24
TXNIP	-0.93	0.0097	1q21.1
TM7SF2	-0.92	0.0368	11q13
CNTFR	-0.92	0.0268	9p13
AMPH	-0.92	0.0446	7p14-p13
TNNT3	-0.92	0.0042	11p15.5
MPPED2	-0.91	0.0014	11p13
POLN	-0.90	0.0271	4p16.3
COLEC12	-0.90	0.0268	18pter-p11.3
CA8	-0.90	0.0420	8q12.1
ZNF540	-0.89	0.0046	19q13.12
SLIT1	-0.89	0.0358	10q23.3-q24
KCNIP2	-0.89	0.0382	10q24
LAMA2	-0.88	0.0147	6q22-q23
RNF157	-0.88	0.0302	17q25.1
F13A1	-0.88	0.0301	6p25.3-p24.3
TSKU	-0.88	0.0306	11q13.5
MIR29C	-0.86	0.0139	1q32.2
TNFAIP8	-0.86	0.0092	5q23.1
HIF3A	-0.85	0.0358	19q13.32
FST	-0.85	0.0298	5q11.2
DDB2	-0.85	0.0136	11p12-p11
DUOX1	-0.83	0.0369	15q15.3
LYNX1	-0.83	0.0177	8q24.3

LINC01354	-0.83	0.0221	NA
HADH	-0.83	0.0099	4q22-q26
POLR2J3	-0.82	0.0308	7q22.1
AVPI1	-0.82	0.0166	10q24.2
DFNA5	-0.82	0.0494	7p15
GLRB	-0.81	0.0380	4q31.3
FAM213A	-0.81	0.0182	10q23.1
ULK4	-0.80	0.0219	3p22.1
CIDEC	-0.80	0.0458	3p25.3
CDKL1	-0.80	0.0214	14q21.3
PFKFB1	-0.79	0.0210	Xp11.21
RAMP2-AS1	-0.79	0.0445	17q21.31
EFNA1	-0.79	0.0041	1q21-q22
CDNF	-0.79	0.0108	10p13
MGST1	-0.78	0.0174	12p12.3-p12.1
PLA2G16	-0.78	0.0272	11q12.3
RGL3	-0.76	0.0340	19p13.2
UGP2	-0.76	0.0192	2p14-p13
HOTAIRM1	-0.76	0.0185	7p15.2
MRPS31P5	-0.75	0.0257	13q
AQP7	-0.75	0.0338	9p13
FZD4	-0.75	0.0199	11q14.2
UST	-0.75	0.0165	6q25.1
STRADB	-0.75	0.0207	2q33.1
PLIN1	-0.74	0.0269	15q26
MDH1	-0.73	0.0212	2p13.3
TYRO3	-0.73	0.0360	15q15
PRKAG2-AS1	-0.73	0.0283	NA
RPARP-AS1	-0.73	0.0405	NA
MYZAP	-0.73	0.0279	15q21.3
AGTR1	-0.72	0.0470	3q24
ADIRF	-0.72	0.0125	10q23.2
RTN1	-0.72	0.0086	14q23.1
TMEM17	-0.72	0.0034	2p15
ACAT1	-0.71	0.0039	11q22.3
HOXB6	-0.71	0.0071	17q21.3
PTPRS	-0.71	0.0112	19p13.3
PHYH	-0.71	0.0017	10p13
YBX2	-0.71	0.0299	17p13.1
E2F1	-0.70	0.0295	20q11.2
PPP1R3E	-0.70	0.0040	14q11.2
MAPKAPK5-AS1	-0.70	0.0014	12q24.12
ITM2A	-0.70	0.0166	Xq13.3-Xq21.2
PHF7	-0.70	0.0066	3p21.1
GPR34	-0.69	0.0194	Xp11.4
PQLC2L	-0.69	0.0143	NA

FAH	-0.69	0.0455	15q25.1
TUB	-0.69	0.0407	11p15.5
EIF4EBP1	-0.69	0.0136	8p12
C6ORF226	-0.69	0.0279	NA
LONRF2	-0.69	0.0494	2q11.2
FAM184B	-0.68	0.0416	4p16
ALDH1A1	-0.68	0.0145	9q21.13
C14ORF79	-0.67	0.0478	NA
MAOB	-0.67	0.0465	Xp11.23
ZNF500	-0.67	0.0020	16p13.3
ZNF19	-0.67	0.0396	16q22
BHMT2	-0.67	0.0430	5q13
FBLN5	-0.66	0.0107	14q32.1
DSE	-0.66	0.0086	6q22
MASP2	-0.66	0.0064	1p36.3-p36.2
FGF14-AS2	-0.66	0.0148	13q33.1
VEGFB	-0.66	0.0327	11q13
PDGFC	-0.65	0.0195	4q32
MRC1	-0.65	0.0241	10p12.33
RAB11B-AS1	-0.65	0.0107	NA
USP30	-0.65	0.0064	12q24.11
TMEM204	-0.65	0.0142	16p13.3
CYB5A	-0.65	0.0141	18q23
PAXIP1-AS1	-0.64	0.0097	7q36.2
BPHL	-0.64	0.0189	6p25
METTL7A	-0.63	0.0169	12q13.12
FRS3	-0.63	0.0197	6p21.1
C19ORF12	-0.63	0.0365	NA
ECHDC2	-0.63	0.0396	1p32.3
C10orf25	-0.63	0.0338	10q11.21
EPOR	-0.63	0.0328	19p13.3-p13.2
GPX4	-0.63	0.0218	19p13.3
RIDA	-0.63	0.0172	NA
LOC100288911	-0.62	0.0203	2p22.3
TNIP1	-0.62	0.0396	5q32-q33.1
URB1-AS1	-0.62	0.0349	NA
PABPN1	-0.62	0.0152	14q11.2
TFAP4	-0.60	0.0187	16p13
ITGB1BP1	-0.60	0.0053	2p25.2
HDDC2	-0.60	0.0044	6q13-q24.3
TNFRSF21	-0.59	0.0234	6p21.1
ZNF229	-0.59	0.0255	19q13.31
C15ORF40	-0.59	0.0043	NA
SPAG16	-0.59	0.0045	2q34
SCRN2	-0.59	0.0473	17q21.32
CORO2B	-0.59	0.0081	15q23

C9orf37	-0.59	0.0101	9q34.3
LPIN3	-0.59	0.0213	20q12
NSUN5P1	-0.59	0.0114	7q11.23
TSPAN6	-0.59	0.0451	Xq22
SDHAF4	-0.59	0.0077	NA
NIFK-AS1	-0.59	0.0200	NA
PPP1R15B	0.59	0.0435	1q32.1
STK4	0.59	0.0211	20q11.2-q13.2
CSK	0.59	0.0096	15q24.1
LLGL2	0.59	0.0324	17q25.1
PTP4A3	0.59	0.0316	8q24.3
ARSG	0.59	0.0403	17q24.2
NEU1	0.59	0.0381	6p21.3
ZNF597	0.59	0.0178	16p13.3
HERC3	0.59	0.0052	4q21
ADRA2C	0.59	0.0333	4p16
TBC1D32	0.60	0.0284	6q22.31
LPCAT2	0.60	0.0076	16q12.2
TET2	0.60	0.0474	4q24
SLC2A1	0.60	0.0294	1p34.2
ZCCHC6	0.60	0.0186	9q21
KMT2E	0.60	0.0085	7q22.1
SDSL	0.60	0.0327	12q24.13
CYP51A1	0.60	0.0242	7q21.2
GCNT2	0.60	0.0362	6p24.2
ASAP1	0.61	0.0052	8q24.1-q24.2
FANCM	0.61	0.0434	14q21.2
MAPK8IP1	0.61	0.0140	11p11.2
CDR2	0.61	0.0176	16p12.3
C21ORF91	0.61	0.0046	NA
PLCG2	0.62	0.0139	16q24.1
TPM4	0.62	0.0378	19p13.1
CEP135	0.62	0.0352	4q12
CHST11	0.62	0.0467	12q
DUSP16	0.62	0.0094	12p13
TRANK1	0.63	0.0381	3p22.2
ZNF831	0.63	0.0354	20q13.32
APAF1	0.63	0.0157	12q23
C9ORF40	0.63	0.0148	NA
MAN2A1	0.64	0.0241	5q21-q22
PPTC7	0.64	0.0383	12q24.11
STK17A	0.64	0.0385	7p13
RCAN3	0.64	0.0146	1p35.3-p33
FAM129B	0.64	0.0148	9q34.13
DYRK3	0.64	0.0447	1q32.1
EOGT	0.65	0.0227	3p14.1

NBEA	0.65	0.0132	13q13
IGDCC4	0.65	0.0387	15q22.31
RHOJ	0.65	0.0040	14q23.2
LBR	0.65	0.0088	1q42.1
PAPLN	0.65	0.0261	14q24.2
CDH24	0.65	0.0088	14q11.2
SFMBT2	0.65	0.0148	10p14
PPP1R12A	0.66	0.0157	12q15-q21
ITM2C	0.66	0.0281	2q37
LUZP1	0.66	0.0180	1p36
NOTCH3	0.66	0.0411	19p13.2-p13.1
ST8SIA4	0.66	0.0332	5q21
ACE	0.66	0.0104	17q23.3
ETV3	0.66	0.0352	1q21-q23
SLC37A1	0.66	0.0113	21q22.3
PGM2L1	0.66	0.0078	11q13.4
PTPRJ	0.67	0.0338	11p11.2
CD93	0.67	0.0224	20p11.21
C3ORF14	0.67	0.0128	NA
FLVCR1	0.67	0.0351	1q32.3
PIK3AP1	0.68	0.0358	10q24.1
WIPF1	0.68	0.0126	2q31.1
HLA-B	0.68	0.0209	6p21.3
FEM1C	0.68	0.0277	5q22
MICAL1	0.68	0.0264	6q21
ITPR1	0.68	0.0438	3p26.1
RAB30	0.68	0.0461	11q12-q14
SRD5A1	0.69	0.0172	5p15
TMEM170B	0.69	0.0354	6p24.2
ZC3HAV1	0.69	0.0085	7q34
PLEKHO1	0.69	0.0287	1q21.2
MALAT1	0.69	0.0232	11q13.1
COL7A1	0.69	0.0493	3p21.1
ZNF281	0.69	0.0128	1q32.1
FAM53C	0.69	0.0426	5q31
TUBA1A	0.70	0.0155	12q13.12
DLG3	0.70	0.0300	Xq13.1
HIF1A	0.70	0.0277	14q23.2
GNA13	0.70	0.0127	17q24.3
TMEM229B	0.70	0.0443	14q24.1
SOWAHC	0.71	0.0042	2q13
SIDT1	0.71	0.0147	3q13.2
SH3BP1	0.71	0.0287	22q13.1
FAM135A	0.72	0.0111	6q13
SYNJ2	0.72	0.0480	6q25.3
RASA2	0.72	0.0463	3q22-q23

PDE4D	0.72	0.0386	5q12
RYBP	0.73	0.0181	3p13
NXPE3	0.73	0.0015	3q12.3
SPN	0.73	0.0358	16p11.2
PLEKHG2	0.73	0.0455	19q13.2
GUCY1B3	0.73	0.0497	4q31.3-q33
STUM	0.73	0.0222	NA
HMGCR	0.74	0.0127	5q13.3-q14
CPEB2	0.74	0.0093	4p15.33
LOC284454	0.74	0.0488	19p13.13
CAPN5	0.75	0.0370	11q14
ZNF548	0.75	0.0049	19q13.43
LRCH2	0.75	0.0027	Xq23
FAM13C	0.75	0.0101	10q21.1
ITGB7	0.76	0.0147	12q13.13
PAG1	0.76	0.0376	8q21.13
DOCK10	0.76	0.0065	2q36.2
TNFRSF10A	0.76	0.0189	8p21
GPR160	0.77	0.0287	3q26.2-q27
CXORF21	0.77	0.0456	NA
PAQR8	0.77	0.0024	6p12.1
STC2	0.77	0.0030	5q35.1
DENND1C	0.77	0.0344	19p13.3
ARL4C	0.77	0.0243	2q37.1
IVNS1ABP	0.77	0.0416	1q25.1-q31.1
THBS1	0.77	0.0318	15q15
ZNF850	0.77	0.0364	19q13.12
LAG3	0.78	0.0197	12p13.32
DNAJB4	0.78	0.0222	1p31.1
ENTPD7	0.78	0.0232	NA
CYP7B1	0.78	0.0463	8q21.3
TRAF4	0.79	0.0301	17q11-q12
ADA2	0.79	0.0379	NA
ZNF154	0.79	0.0145	19q13.4
PPFIA3	0.79	0.0028	19q13.33
BCL2L11	0.80	0.0013	2q13
FHL3	0.80	0.0332	1p34
FOXF1	0.80	0.0237	16q24
OSR2	0.81	0.0064	8q22.2
COL24A1	0.81	0.0433	1p22.3
ARID5B	0.81	0.0187	10q21.2
PTGDS	0.81	0.0236	9q34.2-q34.3
BIRC3	0.81	0.0459	11q22
PPP1R3B	0.81	0.0173	8p23.1
MCL1	0.81	0.0286	1q21
PARP15	0.81	0.0413	3q21.1

PAWR	0.81	0.0116	12q21
SDE2	0.82	0.0029	1q42.12
TUBA4A	0.82	0.0249	2q35
DLC1	0.82	0.0083	8p22
TOX	0.82	0.0099	8q12.1
GEM	0.82	0.0371	8q13-q21
IFFO2	0.82	0.0017	1p36.13
LRRC70	0.82	0.0167	5q12.1
GZMB	0.83	0.0431	14q11.2
LMNA	0.83	0.0489	1q22
ITGAL	0.83	0.0260	16p11.2
ABCG2	0.84	0.0032	4q22
ARHGAP15	0.84	0.0075	2q22.2-q22.3
ARNTL2	0.84	0.0206	12p12.2-p11.2
TGFA	0.84	0.0334	2p13
RASAL3	0.84	0.0243	19p13.12
CX3CL1	0.85	0.0488	16q13
KLF4	0.85	0.0465	9q31
MBOAT2	0.85	0.0176	2p25.1
ITGA5	0.85	0.0288	12q11-q13
SMIM5	0.85	0.0396	17q25.1
ERN1	0.86	0.0070	17q24.2
TMC8	0.86	0.0314	17q25.3
STK17B	0.86	0.0311	2q32.3
TAP1	0.87	0.0172	6p21.3
KLHL6	0.87	0.0287	3q27.3
PELI1	0.87	0.0170	2p13.3
MRVI1	0.87	0.0385	11p15
DYSF	0.87	0.0460	2p13.3
DUSP1	0.87	0.0248	5q34
set/01	0.87	0.0276	16p11.1
RUNDC3B	0.87	0.0393	7q21.12
ADORA2A	0.87	0.0124	22q11.23
RASSF2	0.88	0.0312	20p13
MAP4K1	0.88	0.0179	19q13.1-q13.4
CYFIP2	0.88	0.0193	5q33.3
PRR26	0.88	0.0259	10p15.3
ZSWIM4	0.88	0.0323	19p13.13
PHACTR1	0.88	0.0493	6p24.1
RALGPS2	0.89	0.0042	1q25.2
DNAJC6	0.89	0.0073	1p31.3
NCOA2	0.89	0.0438	8q13.3
SKIL	0.89	0.0470	3q26
AVPR1A	0.89	0.0309	12q14-q15
PRRT1	0.90	0.0191	6p21.32
IGSF10	0.90	0.0344	3q25.1

GALNT17	0.90	0.0169	NA
RASD2	0.91	0.0178	22q13.1
C1ORF226	0.91	0.0060	NA
AHR	0.91	0.0012	7p15
NHSL2	0.91	0.0310	Xq13.1
ZNF93	0.91	0.0228	19p12
SEMA4D	0.91	0.0171	9q22.2
SLC17A9	0.91	0.0076	20q13.33
PSMB9	0.92	0.0072	6p21.3
PRKCB	0.92	0.0224	16p11.2
ANO9	0.92	0.0469	11p15.5
SLC6A1	0.92	0.0089	3p25.3
JUND	0.92	0.0489	19p13.2
INPP4B	0.92	0.0267	4q31.21
UGCG	0.93	0.0460	9q31
FCMR	0.93	0.0249	NA
STK38L	0.93	0.0005	12p11.23
PIK3CG	0.94	0.0046	7q22.3
HBEGF	0.94	0.0435	5q23
IKZF1	0.95	0.0058	7p13-p11.1
KLF6	0.95	0.0062	10p15
ADAM19	0.95	0.0228	5q33.3
CNN2	0.95	0.0014	19p13.3
TMC4	0.95	0.0124	19q13.42
WDFY4	0.96	0.0309	10q11.23
ATP2A3	0.96	0.0078	17p13.3
DOCK5	0.96	0.0120	8p21.2
PCSK7	0.96	0.0094	11q23-q24
GCH1	0.96	0.0046	14q22.1-q22.2
TESPA1	0.97	0.0318	12q13.2
OSBPL10	0.97	0.0067	3p22.3
RUNX3	0.97	0.0138	1p36
SSTR2	0.98	0.0263	17q24
CEP128	0.98	0.0121	14q31.1
CARD11	0.98	0.0210	7p22
KBTBD8	0.98	0.0079	3p14
BRSK1	0.98	0.0309	19q13.4
HAPLN3	0.99	0.0068	15q26.1
KIF21B	0.99	0.0212	1q32.1
IL34	0.99	0.0063	16q22.1
SEMA7A	0.99	0.0125	15q22.3-q23
SCIMP	0.99	0.0329	17p13.2
ADGRL3	0.99	0.0321	NA
BHLHE22	0.99	0.0153	8q13
ISG20	0.99	0.0475	15q26
GPR65	1.00	0.0345	14q31-q32.1

SMAD7	1.00	0.0030	18q21.1
TES	1.00	0.0076	7q31.2
IL2RG	1.01	0.0299	Xq13.1
ATF3	1.01	0.0262	1q32.3
NLRC5	1.01	0.0048	16q13
DUSP8	1.02	0.0160	11p15.5
PCDH17	1.02	0.0171	13q21.1
FBLIM1	1.02	0.0073	1p36.21
TRIM9	1.03	0.0299	14q22.1
RASGRP1	1.03	0.0125	15q14
CD3E	1.03	0.0174	11q23
PRDM1	1.03	0.0066	6q21
LINC00926	1.03	0.0021	15q21.3
PLXNC1	1.03	0.0202	12q23.3
TCF7	1.03	0.0061	5q31.1
EXOC3L4	1.04	0.0042	14q32.32
CD48	1.04	0.0037	1q21.3-q22
RNF19B	1.04	0.0089	1p35.1
SPRY1	1.04	0.0028	4q28.1
SYTL2	1.05	0.0130	11q14
CD3D	1.05	0.0291	11q23
MTCL1	1.05	0.0311	NA
CD5	1.05	0.0275	11q13
NEDD9	1.05	0.0207	6p25-p24
NUP210	1.05	0.0036	3p25.1
SYTL1	1.05	0.0055	1p36.11
TNFRSF10D	1.06	0.0378	8p21
ATP8B1	1.06	0.0045	18q21.31
CORO2A	1.06	0.0161	9q22.3
RASSF5	1.07	0.0177	1q32.1
TPD52	1.07	0.0070	8q21
SRF	1.07	0.0001	6p21.1
TMEM200A	1.07	0.0076	6q23.1
IPCEF1	1.08	0.0158	6q25.2
HEPH	1.08	0.0121	Xq11-q12
PAQR5	1.08	0.0025	15q23
FOSL2	1.09	0.0234	2p23.3
GK	1.09	0.0422	Xp21.3
MIAT	1.09	0.0123	22q12.1
TSC22D2	1.09	0.0113	3q25.1
CD38	1.10	0.0355	4p15
FOS	1.11	0.0307	14q24.3
MPZL3	1.11	0.0243	11q23.3
FYB	1.11	0.0189	5p13.1
ABHD17C	1.11	0.0045	15q25.1
FOXC1	1.12	0.0399	6p25

CD37	1.13	0.0141	19q13.3
MMP16	1.13	0.0211	8q21.3
ITGA4	1.13	0.0015	2q31.3
ZBTB21	1.14	0.0019	21q22.3
MYADM	1.14	0.0162	19q13.42
COL4A4	1.14	0.0302	2q35-q37
CNTN1	1.15	0.0191	12q11-q12
WEE1	1.15	0.0099	11p15.3-p15.1
ITK	1.15	0.0048	5q31-q32
ETV7	1.15	0.0442	6p21
BCL11B	1.16	0.0096	14q32.2
CHSY3	1.16	0.0058	5q23.3
FHL5	1.16	0.0290	6q16.1-q16.3
CARMIL2	1.17	0.0112	NA
TNFRSF11B	1.18	0.0263	8q24
XPNPEP2	1.18	0.0122	Xq25
RYR2	1.18	0.0287	1q43
DUSP5	1.18	0.0013	10q25
SGK1	1.18	0.0117	6q23
CD6	1.18	0.0228	11q13
PHLDA1	1.18	0.0102	12q15
SLC45A3	1.19	0.0078	1q32.1
RET	1.20	0.0014	10q11.2
RNF24	1.20	0.0023	20p13
ADGRE1	1.21	0.0496	NA
CSRNP1	1.22	0.0325	3p22
SEMA4A	1.22	0.0465	1q22
PTPN22	1.22	0.0089	1p13.2
VDR	1.22	0.0069	12q13.11
STARD4	1.23	0.0118	5q22.1
LINC00312	1.25	0.0061	3p25.3
BASP1	1.25	0.0358	5p15.1
TRIB1	1.26	0.0242	8q24.13
COL3A1	1.26	0.0410	2q31
CLDN7	1.26	0.0451	NA
SLC2A3	1.27	0.0328	12p13.3
LYZ	1.27	0.0324	12q15
BATF2	1.27	0.0138	11q13.1
NAPSB	1.27	0.0367	19q13.33
ADAMTS14	1.27	0.0174	10q21
EGR3	1.28	0.0212	8p23-p21
HSH2D	1.29	0.0146	19p13.12
DAPP1	1.29	0.0157	4q25-q27
FILIP1L	1.30	0.0117	3q12.1
GALNT3	1.30	0.0073	2q24-q31
FAM26F	1.32	0.0398	6q22.1

ADAM28	1.32	0.0133	8p21.2
APOLD1	1.32	0.0189	12p13.1
RUNX2	1.32	0.0076	6p21
SLC16A6	1.34	0.0244	17q24.2
ST6GAL2	1.34	0.0219	2q11.2-q12.1
CPNE5	1.36	0.0113	6p21.1
KCNN4	1.36	0.0018	19q13.2
NRXN3	1.36	0.0191	14q31
CASS4	1.37	0.0054	20q13.31
COL14A1	1.37	0.0127	8q23
EDIL3	1.39	0.0380	5q14
HLA-DRB5	1.39	0.0131	6p21.3
HOPX	1.40	0.0226	4q12
LEF1	1.40	0.0063	4q23-q25
SSUH2	1.40	0.0304	3p26.1
SPAG4	1.41	0.0056	20q11.21
CYTIP	1.41	0.0104	2q11.2
ABCC3	1.41	0.0001	17q22
SERPINA1	1.42	0.0245	14q32.1
SIK1	1.44	0.0155	21q22.3
PDGFD	1.44	0.0050	11q22.3
ADAMTS1	1.45	0.0036	21q21.2
MS4A2	1.45	0.0444	11q12-q13
ANPEP	1.48	0.0321	15q25-q26
LGALS2	1.49	0.0186	22q13.1
FZD10-AS1	1.50	0.0267	12q24.33
CYR61	1.50	0.0005	1p22.3
P2RY10	1.50	0.0005	Xq21.1
TAGAP	1.52	0.0073	6q25.3
SPINT1	1.53	0.0443	15q15.1
PLA2G7	1.54	0.0188	6p21.2-p12
AIM2	1.54	0.0125	1q22
RNF152	1.56	0.0009	18q21.33
RHOH	1.56	0.0008	4p13
SP140	1.57	0.0001	2q37.1
PDE4B	1.57	0.0009	1p31
GBP1	1.58	0.0011	1p22.2
IL7R	1.59	0.0003	5p13
TREH	1.60	0.0242	11q23.3
PLAC8	1.61	0.0066	4q21.22
FHL2	1.61	0.0007	2q12.2
HLA-DOB	1.63	0.0057	6p21.3
F2RL1	1.63	0.0131	5q13
ZBP1	1.66	0.0016	20q13.31
CXCR4	1.67	0.0003	2q21
CD69	1.67	0.0042	12p13

CCR7	1.68	0.0011	17q12-q21.2
RGS1	1.70	0.0006	1q31
ITIH3	1.70	0.0359	3p21.1
FOSB	1.71	0.0078	19q13.32
SLC7A5	1.73	0.0471	16q24.3
LTB	1.73	0.0051	6p21.3
LDLR	1.74	0.0129	19p13.2
SLC2A5	1.75	0.0432	1p36.2
LY9	1.78	0.0038	1q23.3
P2RX1	1.79	0.0469	17p13.3
ANKRD22	1.79	0.0458	10q23.31
CTGF	1.81	0.0025	6q23.1
COL17A1	1.81	0.0303	10q24.3
KIAA1211	1.83	0.0043	4q12
IRF4	1.84	0.0013	6p25-p23
LAX1	1.86	0.0004	1q32.1
GBP5	1.89	0.0161	1p22.2
FAM46C	1.95	0.0001	1p12
CD24	1.95	0.0369	6q21
TDO2	2.01	0.0225	4q31-q32
UBD	2.01	0.0225	6p21.3
FCAR	2.06	0.0417	19q13.42
SHROOM3	2.08	0.0289	4q21.1
PI15	2.08	0.0456	8q21.11
MEI1	2.09	0.0001	22q13.2
CLC	2.09	0.0003	19q13.1
NR4A3	2.11	0.0387	9q22
TMC5	2.14	0.0403	16p12.3
CNN1	2.15	0.0483	19p13.2-p13.1
PIM2	2.18	0.0001	Xp11.23
CP	2.25	0.0009	3q23-q25
CTLA4	2.30	0.0047	2q33
SLAMF7	2.31	0.0016	1q23.1-q24.1
BMS1P20	2.32	0.0033	NA
APOL4	2.32	0.0149	22q11.2-q13.2
SLC44A4	2.40	0.0121	6p21.3
EREG	2.45	0.0398	4q13.3
CCL11	2.50	0.0001	17q12
ECEL1	2.52	0.0008	2q37.1
PCDH7	2.67	0.0026	4p15
TSPAN1	2.68	0.0028	1p34.1
LGALS4	2.80	0.0338	19q13.2
ANKRD36BP2	2.83	0.0000	2p11.2
SDC1	2.91	0.0000	2p24.1
CYP4F35P	2.98	0.0000	18p11.21
DERL3	3.06	0.0000	22q11.23

CCL19	3.31	0.0009	9p13
MS4A1	3.50	0.0024	11q12
JCHAIN	3.59	0.0000	NA
FCRL5	3.73	0.0000	1q21
POU2AF1	3.81	0.0000	11q23.1
CD79A	3.87	0.0000	19q13.2
ALDOB	3.95	0.0205	9q21.3-q22.2
MZB1	4.09	0.0000	5q31.2
IGLL5	4.17	0.0000	22q11.22
FAM30A	4.49	0.0000	NA
MIR650	4.55	0.0000	22q11.22
MUC2	4.63	0.0101	11p15.5
OLFM4	4.84	0.0024	13q14.3
PIGR	5.02	0.0111	1q31-q41
ADAMDEC1	5.12	0.0009	8p21.2
REG1A	5.31	0.0149	2p12
CLCA1	5.69	0.0053	1p22.3
