# Supplementary material for: Whole transcriptional analysis identifies markers of B, T and plasma cell signaling pathways in the mesenteric adipose tissue associated with Crohn’s disease
Source: J Transl Med. 2020 Jan 30;18:44. doi: 10.1186/s12967-020-02220-3 (PMC6993458; doi:10.1186/s12967-020-02220-3)
Supplement: Supplementary file 7 — Additional file 7. List of 2.654 differentially expressed genes identified by RNA-Seq (p < 0.05) in the ileal mucosa of Crohn’s disease patients compared to the controls sorted by log2 fold change. [file 12967_2020_2220_MOESM7_ESM.pdf]

Additional file 7. List of 2.654 differentially expressed genes identified by RNA-Seq (P < 0.05) in the ileal mucosa of Crohn's disease patients compared to the controls sorted by Log2 fold change.

Gene	Locus	Log2 Fold Change	P-value
SLC13A1	7q31.32	-6.88	0.00128
GUCA2B	1p34-p33	-6.59	0.00033
FABP6	5q33.3-q34	-6.51	0.00272
G6PC	17q21	-6.43	0.00104
TMIGD1	17q11.2	-6.16	0.00435
SLC10A2	13q33	-6.06	0.00764
CDHR1	10q23.1	-6.00	0.00004
CPO	2q33.3	-5.78	0.00144
SLC17A8	12q23.1	-5.77	0.00064
CUBN	10p12.31	-5.74	0.00010
HSD3B1	1p13.1	-5.68	0.00151
GUCA2A	1p35-p34	-5.37	0.00522
SLC5A12	11p14.2	-5.35	0.00549
FADS6	17q25.1	-5.33	0.00027
SOAT2	12q13.13	-5.31	0.00004
FAM151A	NA	-5.20	0.00006
SLC28A2	15q15	-5.11	0.00840
FRMD1	6q27	-5.08	0.00018
PNLIPRP2	10q25.3	-5.05	0.01608
APOA1	11q23-q24	-5.03	0.01107
CYP3A4	7q21.1	-5.00	0.01726
SLC28A1	15q25.3	-4.94	0.01509
APOC3	11q23.3	-4.86	0.01208
CDKN2B-AS1	9p21.3	-4.83	0.00009
CYP4F2	19p13.12	-4.79	0.02053
KCNH6	17q23.3	-4.78	0.00005
PKLR	1q21	-4.70	0.00163

MS4A10	11q12.2	-4.69	0.02913
NTS	12q21	-4.58	0.00336
TMEM252	9q21.11	-4.56	0.00019
MEP1B	18q12.2-q12.3	-4.53	0.01485
PLB1	2p23.2	-4.49	0.00010
CNTFR	9p13	-4.48	0.00119
SLC6A19	5p15.33	-4.42	0.01886
APOA4	11q23	-4.40	0.04083
SLC7A9	19q13.1	-4.38	0.01452
SLC5A11	16p12.1	-4.35	0.00050
SLC6A4	17q11.2	-4.35	0.00769
ZG16	16p11.2	-4.34	0.02448
NAALADL1	11q12	-4.34	0.00006
CYP4F11	19p13.1	-4.31	0.01432
KCNJ13	2q37	-4.28	0.01719
SLC3A1	2p16.3	-4.27	0.02777
APOB	2p24-p23	-4.25	0.02309
CEACAM20	19q13.31	-4.24	0.01177
FMO1	1q24.3	-4.18	0.00012
DPEP1	16q24.3	-4.18	0.00164
PRKG2	4q13.1-q21.1	-4.17	0.00067
ITLN2	1q22-q23	-4.14	0.02818
EDN2	1p34	-4.08	0.02095
CYP2B6	19q13.2	-4.03	0.00990
PYY	17q21.1	-3.99	0.00338
HRASLS2	11q12.3	-3.95	0.03081
SULT2A1	19q13.3	-3.95	0.04843
SLC15A1	13q32.3	-3.89	0.04411
SUSD2	22q11-q12	-3.83	0.00023
XPNPEP2	Xq25	-3.81	0.01010
LINC00955	4p16.3	-3.79	0.02414
DHDH	19q13.3	-3.74	0.00368
B4GALNT2	17q21.32	-3.72	0.00827
SLC23A1	5q31.2	-3.65	0.00124

CYP2J2	1p31.3-p31.2	-3.63	0.04269
AQP7	9p13	-3.62	0.00618
CAPN13	2p22-p21	-3.62	0.03296
SFRP5	10q24.1	-3.62	0.02315
TMEM229A	7q31.32	-3.60	0.00337
AADAC	3q25.1	-3.60	0.03770
MEP1A	6p12-p11	-3.57	0.02514
SMLR1	6q23.1	-3.48	0.04804
ENPEP	4q25	-3.47	0.00578
MGAM	7q34	-3.47	0.00086
LEAP2	5q31.1	-3.46	0.00206
IGSF9	1q22-q23	-3.46	0.00621
KCNG1	20q13	-3.45	0.00000
NAT8B	2p13.1	-3.44	0.00316
GABRA2	4p12	-3.43	0.00756
PRODH	22q11.21	-3.42	0.00632
PDZK1	1q21	-3.39	0.01193
SLC23A3	2q35	-3.39	0.00007
SLC34A3	9q34	-3.39	0.00029
GGT8P	2p11.1	-3.30	0.00086
OTOP3	17q25.1	-3.28	0.04288
CA7	16q22.1	-3.27	0.01085
SSUH2	3p26.1	-3.26	0.01045
GDPD2	Xq13.1	-3.26	0.00590
ZNF488	10q11.22	-3.25	0.00837
NPY6R	5q31.2	-3.24	0.02142
CYP2D6	22q13.1	-3.24	0.00023
OIT3	10q22.1	-3.20	0.01102
UGT1A10	2q37	-3.18	0.03101
SLC5A9	1p33	-3.18	0.01064
B3GALT5	21q22.3	-3.14	0.02906
PTPRR	12q15	-3.12	0.00594
SLC5A4	22q12.3	-3.12	0.00064
PKIB	6q22.31	-3.11	0.00155

DAB1	1p32-p31	-3.06	0.01207
FAM21B	10q11.22	-3.06	0.04966
TBX10	11q13.2	-3.05	0.03930
ALDOC	17cen-q12	-3.04	0.00000
SCIN	7p21.3	-3.03	0.00883
LINC00511	17q24.3	-2.99	0.00739
SULT1A2	16p12.1	-2.99	0.03581
C6ORF222	NA	-2.98	0.01037
ABCB1	7q21.12	-2.98	0.00433
PON3	7q21.3	-2.98	0.00084
CNGA1	4p12	-2.97	0.00220
CCR9	3p21.3	-2.97	0.01715
ABCG2	4q22	-2.95	0.01139
GCNT4	5q12	-2.95	0.00024
BEST4	1p33-p32.3	-2.92	0.00497
HAPLN4	19p13.1	-2.91	0.04260
TRPM6	9q21.13	-2.91	0.00067
NEU4	2q37.3	-2.91	0.00213
SLC1A7	1p32.3	-2.90	0.00382
NPY4R	10q11.2	-2.90	0.02615
TREH	11q23.3	-2.90	0.03817
CR2	1q32	-2.89	0.00524
CHRNA7	15q14	-2.85	0.00343
GAL3ST1	22q12.2	-2.84	0.00313
MIR192	11q13.1	-2.84	0.01415
MMEL1	1p36	-2.83	0.00011
MRO	18q21	-2.83	0.00397
ABO	9q34.2	-2.82	0.01175
SOWAHB	4q21.1	-2.81	0.03896
MYOM3	1p36.11	-2.81	0.00062
CLEC4F	2p13.3	-2.80	0.00169
DNASE1	16p13.3	-2.80	0.00627
CBS	21q22.3	-2.76	0.00004
REEP6	19p13.3	-2.76	0.03510

ABCC2	10q24	-2.76	0.00547
SUGCT	NA	-2.73	0.00044
GPRIN2	10q11.22	-2.72	0.04820
CEACAM18	19q13.41	-2.69	0.04244
PDZD7	10q24.31	-2.66	0.01403
SLC6A8	Xq28	-2.65	0.00043
SLC39A4	8q24.3	-2.65	0.00423
TRHDE	12q15-q21	-2.65	0.01200
NEURL3	2q11.2	-2.64	0.00004
KLKB1	4q35	-2.63	0.00235
SLITRK6	13q31.1	-2.63	0.02599
KHK	2p23.3	-2.59	0.01946
CHAD	17q21.33	-2.59	0.00095
LOC100130899	22q13.1	-2.59	0.04546
PAQR5	15q23	-2.56	0.00000
PHLPP2	16q22.2	-2.54	0.00072
INPP5J	22q12.2	-2.50	0.04199
LGALS2	22q13.1	-2.50	0.00487
HAGLR	NA	-2.50	0.00522
SPIB	19q13.3-q13.4	-2.49	0.00012
HOXB6	17q21.3	-2.48	0.03940
ENTPD5	14q24	-2.46	0.00272
CDKN2B	9p21	-2.44	0.00459
TRIM10	6p21.3	-2.44	0.04980
C2ORF88	NA	-2.44	0.00024
AGAP11	10q23.2	-2.43	0.00187
SULT2B1	19q13.3	-2.43	0.03131
STBD1	4q21.1	-2.43	0.00074
AKR7L	1p35-p36.1;1p36.13	-2.42	0.03866
FGFR3	4p16.3	-2.42	0.00797
NEUROD1	2q32	-2.42	0.02623
ACE	17q23.3	-2.41	0.00324
CHDH	3p21.1	-2.40	0.00199
HOXB-AS3	17q21.32	-2.39	0.01596

SLC51B	15q22.31	-2.38	0.04962
LOC283177	11q25	-2.38	0.01737
TRPV3	17p13.2	-2.37	0.00006
PHYHIPL	10q11	-2.36	0.00730
CCDC152	5p12	-2.36	0.00811
HTR4	5q31-q33	-2.35	0.03302
ESRRG	1q41	-2.34	0.03297
ASPA	17p13.3	-2.33	0.02236
SLC1A1	9p24	-2.32	0.00500
ANPEP	15q25-q26	-2.30	0.04186
TMEM220	17p13.1	-2.30	0.00265
PRLR	5p13.2	-2.29	0.01039
MAOA	Xp11.3	-2.28	0.01054
SELENOP	NA	-2.28	0.01922
MELTF	NA	-2.26	0.00314
DPP4	2q24.3	-2.26	0.00031
OVOL1	11q13	-2.26	0.04212
FGF9	13q11-q12	-2.25	0.01816
TTC22	1p32.3	-2.24	0.00153
SLC52A3	20p13	-2.23	0.01170
TDP2	6p22.3-p22.1	-2.22	0.00018
CHST6	16q22	-2.21	0.00022
P2RY1	3q25.2	-2.19	0.00158
AOC1	7q36.1	-2.18	0.00572
ADIRF	10q23.2	-2.18	0.04320
CLIC5	6p12.3	-2.18	0.00468
DHRS11	17q12	-2.17	0.04951
EPB41L4B	9q31-q32	-2.17	0.04750
FMO5	1q21.1	-2.16	0.02185
F10	13q34	-2.16	0.00068
ACOX2	3p14.3	-2.16	0.00121
CES2	16q22.1	-2.16	0.01531
APOM	6p21.33	-2.15	0.00115
RUNDC3B	7q21.12	-2.15	0.00054

LAMA1	18p11.3	-2.15	0.00148
GDA	9q21.13	-2.14	0.02520
MAOB	Xp11.23	-2.14	0.01185
CRIP1	14q32.33	-2.13	0.00383
PLCH2	1p36.32	-2.13	0.00057
CYP4F3	19p13.2	-2.13	0.00022
NAGS	17q21.31	-2.12	0.00509
ELL3	15q15.3	-2.12	0.02019
IL17RB	3p21.1	-2.12	0.00017
KCP	7q32.1	-2.11	0.00058
CDK20	9q22.1	-2.10	0.00053
HOXA5	7p15.2	-2.10	0.00618
NCKAP5	2q21.2	-2.10	0.00021
GRAMD1C	3q13.31	-2.09	0.00160
IGSF3	1p13	-2.09	0.00176
TMEM37	2q14.2	-2.09	0.00954
PEPD	19q13.11	-2.09	0.00033
HTR1D	1p36.3-p34.3	-2.08	0.03579
SMPD3	16q22.1	-2.08	0.01694
NELL2	12q12	-2.08	0.00982
FLVCR1	1q32.3	-2.07	0.00135
AQP3	9p13	-2.07	0.00034
NIPAL1	4p12	-2.07	0.02469
NLRP6	11p15	-2.07	0.02081
PTPRD	9p23-p24.3	-2.05	0.00420
SLC9A3	5p15.3	-2.05	0.04726
THSD4	15q23	-2.05	0.00462
WSCD1	17p13.2	-2.04	0.01740
PFKFB4	3p22-p21	-2.04	0.00005
FCER1A	1q23	-2.04	0.01114
SLC14A2	18q12.1-q21.1	-2.04	0.03238
COL17A1	10q24.3	-2.04	0.04792
BTNL8	5q35.3	-2.04	0.04896
SEMA3G	3p21.1	-2.02	0.00106

MALL	2q13	-2.02	0.01566
LIPE	19q13.2	-2.01	0.00005
USP2	11q23.3	-2.00	0.03987
GNG4	1q42.3	-2.00	0.00017
LOC100129046	1p22.1	-1.99	0.01979
BEND7	10p13	-1.99	0.03484
OAT	10q26	-1.98	0.01219
AQP11	11q14.1	-1.97	0.02752
CD160	1q21.1	-1.96	0.00012
GALNT14	2p23.1	-1.96	0.00100
SLC26A2	5q31-q34	-1.95	0.00059
MME	3q25.2	-1.95	0.02042
TMEM92	17q21.33	-1.94	0.04072
EPPK1	8q24.3	-1.94	0.02344
ABHD6	3p14.3	-1.94	0.00280
ABCA8	17q24	-1.94	0.02508
PTGR1	9q31.3	-1.93	0.01394
CA3	8q21.2	-1.93	0.03511
MST1L	1p36.13	-1.92	0.01981
CHN2	7p15.3	-1.92	0.01275
LINC01123	NA	-1.91	0.01531
EFNA1	1q21-q22	-1.90	0.00153
OSR2	8q22.2	-1.89	0.00193
MGAT3	22q13.1	-1.88	0.00002
OSGIN1	16q23.3	-1.88	0.01810
LHFPL3-AS2	NA	-1.85	0.03440
EGLN3	14q13.1	-1.85	0.02457
ACOT11	1p32.3	-1.85	0.03634
WNK2	9q22.3	-1.84	0.03320
PRKCZ	1p36.33-p36.2	-1.84	0.00217
EPS8L2	11p15.5	-1.84	0.03058
SLC22A4	5q31.1	-1.84	0.00092
MMP28	17q21.1	-1.83	0.01065
SLC36A1	5q33.1	-1.83	0.00048

SLC27A2	15q21.2	-1.82	0.02746
TPH1	11p15.3-p14	-1.82	0.03175
P2RY2	11q13.5-q14.1	-1.82	0.00025
ACOT4	14q24.3	-1.81	0.00753
PCSK5	9q21.3	-1.81	0.01098
PFN2	3q25.1	-1.81	0.00018
GRAMD1B	11q24.1	-1.80	0.00428
CDC42BPG	11q13.1	-1.80	0.02089
LOC100130705	7q32.1	-1.79	0.00170
CNTNAP2	7q35	-1.78	0.00121
HCN3	1q22	-1.78	0.00205
FMO4	1q24.3	-1.78	0.00768
RGMB-AS1	5q21.1	-1.78	0.00042
MICALCL	11p15.3	-1.78	0.00308
CLCN2	3q27-q28	-1.78	0.00586
CD8B	2p12	-1.77	0.00022
MST1P2	1p36.2	-1.77	0.01068
SLC25A34	1p36.21	-1.77	0.01042
GATM	15q21.1	-1.76	0.02399
KDM8	16p12.1	-1.76	0.00192
RETSAT	2p11.2	-1.76	0.00082
FRK	6q21-q22.3	-1.75	0.03535
KIAA1161	9p13.3	-1.74	0.04245
CD1C	1q22-q23	-1.74	0.03149
SLC29A4	7p22.1	-1.74	0.00356
SLC22A5	5q23.3	-1.73	0.01013
AATK	17q25.3	-1.72	0.00772
CEBPA-AS1	19q13.11	-1.72	0.03491
BPHL	6p25	-1.72	0.00350
NAAA	4q21.1	-1.71	0.00002
GPR174	Xq21.1	-1.71	0.01164
CYP2U1	4q25	-1.70	0.00103
LINC00654	20p12.3	-1.70	0.00166
C1ORF115	NA	-1.69	0.00311

ZSWIM5	1p34.1	-1.68	0.01140
PAPSS2	10q24	-1.68	0.00145
SLC19A3	2q37	-1.67	0.00068
CDA	1p36.2-p35	-1.67	0.01009
ZNF57	19p13.3	-1.66	0.00389
HOOK1	1p32.1	-1.66	0.02701
BAIAP3	16p13.3	-1.65	0.00081
KIAA1211	4q12	-1.65	0.02752
DFNA5	7p15	-1.65	0.00957
PRAG1	NA	-1.64	0.00118
PROB1	5q31.2	-1.64	0.00243
KCNK5	6p21	-1.64	0.03878
DEPDC7	11p13	-1.64	0.02700
TPRN	9q34.3	-1.64	0.04224
SLC30A4	15q21.1	-1.63	0.00181
SLC35G1	10q23.33	-1.63	0.04487
SEMA3B	3p21.3	-1.63	0.02775
PIGZ	3q29	-1.63	0.00536
DHRS7	14q23.1	-1.62	0.00034
LGR5	12q22-q23	-1.62	0.01293
SLC46A3	13q12.3	-1.62	0.00200
ACSF2	17q21.33	-1.61	0.00026
GNA11	19p13.3	-1.61	0.01652
RHOBTB2	8p21.3	-1.60	0.00367
DNASE1L3	3p14.3	-1.60	0.01140
LINC00526	18p11.31	-1.60	0.00330
GCHFR	15q15	-1.60	0.04190
PTPRF	1p34	-1.60	0.03178
SLC14A1	18q11-q12	-1.60	0.00062
SYT7	11q12-q13.1	-1.60	0.00719
RAG1	11p13	-1.59	0.03886
SEMA4G	10q24.31	-1.59	0.04433
RNF152	18q21.33	-1.59	0.00665
NHSL1	6q23.3	-1.58	0.03024

MARC2	1q41	-1.57	0.00470
TIAM2	6q25.2	-1.57	0.01037
TRPV1	17p13.2	-1.57	0.00080
LINC00673	NA	-1.57	0.00530
SLC9A3R1	17q25.1	-1.56	0.01733
LINC01268	NA	-1.56	0.02825
CDC14A	1p21	-1.56	0.00157
GGT1	22q11.23	-1.56	0.01376
TMEM25	11q23.3	-1.56	0.01521
LOC646471	1p36.11	-1.55	0.01498
TKFC	NA	-1.55	0.03481
TCN2	22q12.2	-1.55	0.00099
PC	11q13.4-q13.5	-1.55	0.00005
COL4A3	2q36-q37	-1.54	0.01291
ZNF439	19p13.2	-1.54	0.00026
ASB13	10p15.1	-1.54	0.00222
RDH5	12q13-q14	-1.54	0.00556
MAF	16q22-q23	-1.53	0.00128
SATB2	2q33	-1.53	0.00413
LRP4	11p11.2	-1.53	0.00961
KIAA1456	8p22	-1.52	0.00238
GPR55	2q37	-1.52	0.00416
GPR18	13q32	-1.52	0.00384
XYLB	3p22-p21.3	-1.52	0.00471
VSIG10	12q24.23	-1.52	0.01888
IFNLR1	1p36.11	-1.51	0.00343
MPP6	7p15	-1.50	0.00097
SMPDL3A	6q22.31	-1.50	0.00031
PDXP	22q12.3	-1.50	0.00802
RORC	1q21	-1.49	0.00402
MIR210HG	11p15.5	-1.48	0.01894
BMP8B	1p35-p32	-1.48	0.00312
ADAM22	7q21	-1.48	0.00178
RHOU	1q42.11-q42.3	-1.47	0.00261

KAZALD1	10q24.31	-1.46	0.01927
C9ORF40	NA	-1.46	0.01934
ANK3	10q21	-1.46	0.02685
SLC25A15	13q14	-1.46	0.01648
RMDN2	2p22.2	-1.45	0.00219
PXMP2	12q24.33	-1.45	0.04701
TBX3	12q24.21	-1.45	0.00026
RGS13	1q31.2	-1.45	0.00199
RRAS2	11p15.2	-1.44	0.00270
CYB5A	18q23	-1.44	0.00624
ABLIM2	4p16.1	-1.44	0.00053
TNFSF14	19p13.3	-1.44	0.01083
EXOC6B	2p13.2	-1.44	0.00125
LINC00926	15q21.3	-1.44	0.03668
SULT1A1	16p12.1	-1.44	0.01416
SHMT1	17p11.2	-1.43	0.00573
TSPAN12	7q31.31	-1.43	0.00726
ACO2	22q13.2	-1.43	0.00421
STRADB	2q33.1	-1.43	0.00813
GOLGA2P5	12q23.1	-1.43	0.00213
NEURL1B	5q35.1	-1.43	0.00328
PPL	16p13.3	-1.42	0.01080
HEBP1	12p13.1	-1.41	0.01221
PPARA	22q13.31	-1.40	0.00160
PAX8	2q13	-1.40	0.00161
LPIN3	20q12	-1.40	0.01419
ADAMTS17	15q24	-1.40	0.00071
SLC37A4	11q23.3	-1.40	0.01609
CBR1	21q22.13	-1.40	0.04132
MATN2	8q22	-1.40	0.00819
ABCB4	7q21.1	-1.40	0.00304
GGN	19q13.2	-1.40	0.03080
BCO2	11q23.1	-1.39	0.00350
TMED6	16q22.1	-1.39	0.01471

FLVCR2	14q24.3	-1.39	0.00086
MYH3	17p13.1	-1.38	0.00128
PTPN3	9q31	-1.37	0.02467
FAM161B	14q24.3	-1.37	0.00314
LINC00294	11p13	-1.37	0.00059
CYBRD1	2q31.1	-1.37	0.02510
LOC645513	4q26	-1.36	0.00024
PDK2	17q21.33	-1.36	0.00084
PRSS36	16p11.2	-1.36	0.00340
WWC1	5q34	-1.36	0.00322
C1ORF220	NA	-1.36	0.01499
NR5A2	1q32.1	-1.36	0.00956
CYP2S1	19q13.1	-1.35	0.01391
FAM213A	10q23.1	-1.35	0.00094
ANO5	11p14.3	-1.35	0.01281
AGBL2	11p11.2	-1.35	0.04258
NAPEPLD	7q22.1	-1.35	0.00983
AHRR	5p15.3	-1.35	0.00195
DOK4	16q21	-1.35	0.02149
TRIM3	11p15.5	-1.34	0.00075
MATK	19p13.3	-1.34	0.00171
HOOK2	19p13.2	-1.34	0.00753
USP37	2q35	-1.34	0.00814
ABCD1	Xq28	-1.34	0.00044
SEMA6C	1q21.2	-1.33	0.00568
THRB	3p24.2	-1.33	0.01770
SLC2A5	1p36.2	-1.33	0.01021
GOLIM4	3q26.2	-1.33	0.01109
RALGPS1	9q33.3	-1.33	0.03404
DAPK2	15q22.31	-1.33	0.00650
ARG2	14q24.1	-1.32	0.01196
SETD9	5q11.2	-1.32	0.00221
TMEM198	2q35	-1.32	0.00627
CPNE2	16q13	-1.32	0.00209

PLOD2	3q24	-1.32	0.02255
SERPINF2	17p13	-1.32	0.00560
GPAT3	NA	-1.32	0.02300
CARMIL3	NA	-1.32	0.00156
MPP5	14q23.3	-1.32	0.00202
NS3BP	11p15.5	-1.31	0.00099
FRRS1	1p21.2	-1.31	0.00714
SLC7A7	14q11.2	-1.31	0.00134
SSPO	7q36.1	-1.31	0.00193
MARCH8	10q11.21	-1.31	0.02750
TLR3	4q35	-1.31	0.01086
FASLG	1q23	-1.30	0.01619
BCAS4	20q13.13	-1.30	0.01249
ATP1A1	1p21	-1.30	0.01506
FRMD3	9q21.32	-1.30	0.01042
CYP2D7	NA	-1.30	0.01238
FAM109A	12q24.12	-1.30	0.02150
ADCY9	16p13.3	-1.30	0.00128
ULK3	15q24.1	-1.29	0.00135
AQP1	7p14	-1.29	0.00411
CLUHP3	16p11.2	-1.28	0.01756
NBPF1	1p36.13	-1.28	0.02506
TCP11L1	11p13	-1.28	0.00124
DDO	6q21	-1.27	0.00435
GDPD1	17q22	-1.27	0.01048
CIB2	15q24	-1.27	0.04769
SLC25A23	19p13.3	-1.27	0.00346
FAT2	5q33.1	-1.26	0.00291
SERPINA1	14q32.1	-1.26	0.00110
APPL2	12q24.1	-1.26	0.00165
PAX8-AS1	NA	-1.25	0.02671
ABCA10	17q24	-1.25	0.04852
SLC22A23	6p25.2	-1.25	0.03085
TMEM116	12q24.13	-1.25	0.00076

NMUR1	2q37.1	-1.24	0.00676
PRKAB2	1q21.1	-1.24	0.00018
NDRG1	8q24.3	-1.23	0.00003
ADAMTSL5	19p13.3	-1.23	0.01185
CIDEB	14q12	-1.23	0.00825
OSBPL6	2q32.1	-1.23	0.01482
SEPHS2	16p11.2	-1.23	0.00208
SAT2	17p13.1	-1.23	0.00772
RYR1	19q13.1	-1.23	0.01214
AGAP8	10q11.23	-1.23	0.04415
BMF	15q14	-1.22	0.00183
ABCB9	12q24	-1.22	0.01663
KLC4	6p21.1	-1.22	0.01253
PLA2R1	2q23-q24	-1.22	0.00319
TTC38	22q13	-1.22	0.02536
FSD1L	9q31	-1.22	0.00243
ZBTB7B	1q21.3	-1.22	0.00222
CAMK2N1	1p36.12	-1.22	0.04697
CERKL	2q31.3	-1.21	0.00549
PEX26	22q11.21	-1.21	0.00439
BCAR3	1p22.1	-1.21	0.00239
SLC26A1	4p16.3	-1.21	0.00932
IFIT1	10q23.31	-1.21	0.04005
DHRS4-AS1	14q11.2	-1.21	0.00140
ACOT1	14q24.3	-1.21	0.03888
VILL	3p21.3	-1.21	0.02731
PCDH1	5q31.3	-1.20	0.00823
TMEM56	1p21.3	-1.20	0.01569
ZNF91	19p12	-1.20	0.00622
PHYH	10p13	-1.20	0.01980
SPATA24	5q31.2	-1.19	0.00406
PPARGC1A	4p15.1	-1.19	0.00542
CASZ1	1p36.22	-1.19	0.01878
CYP27A1	2q33-qter	-1.19	0.00717

ABAT	16p13.2	-1.19	0.02977
QPRT	16p11.2	-1.18	0.00041
DERA	12p12.3	-1.18	0.00207
AMOT	Xq23	-1.18	0.03059
MGAT4A	2q12	-1.18	0.00694
ACBD4	17q21.31	-1.17	0.00520
NUAK2	1q32.1	-1.17	0.00036
LMTK3	19q13.33	-1.17	0.00906
TMEM74B	20p13	-1.17	0.04190
TMIGD2	19p13.3	-1.17	0.02321
NHEJ1	2q35	-1.17	0.00195
ZNF540	19q13.12	-1.17	0.00108
ZZEF1	17p13.2	-1.16	0.00237
RMDN3	15q15.1	-1.16	0.00377
SCN4B	11q23.3	-1.16	0.03624
ZDHHC23	3q13.31	-1.16	0.00096
VWA7	6p21.33	-1.16	0.00813
PGRMC1	Xq22-q24	-1.16	0.00072
VAV3	1p13.3	-1.16	0.00788
CCDC85C	14q32.31	-1.16	0.00373
CTNND1	11q11	-1.16	0.00744
MSRA	8p23.1	-1.15	0.00981
MCM8	20p12.3	-1.15	0.00026
NEURL2	20q13.12	-1.15	0.00823
TMEM120A	7q11.23	-1.15	0.02069
DPF3	14q24.2	-1.15	0.00978
ANKEF1	20p12.2	-1.15	0.00234
LOC646214	15q11.2	-1.15	0.02336
ADPRHL1	13q34	-1.15	0.02377
NSMF	9q34.3	-1.15	0.00298
ETFDH	4q32-q35	-1.14	0.01961
DYRK2	12q15	-1.14	0.01125
CDCA3	12p13	-1.14	0.04983
DHRS4	14q11.2	-1.14	0.03465

RAB3IP	12q15	-1.14	0.01160
DOLPP1	9q34.1	-1.13	0.01025
TSNARE1	8q24.3	-1.13	0.03202
ACADM	1p31	-1.13	0.00288
ACSS1	20p11.23-p11.21	-1.13	0.00078
ATP8A1	4p13	-1.13	0.00872
MTMR4	17q22-q23	-1.13	0.00013
SMG1P7	NA	-1.13	0.01612
ATG4D	19p13.2	-1.12	0.00225
CNKSR3	6q25.2	-1.12	0.00339
TMEM41A	3q27.2	-1.12	0.02978
STYK1	12p13.2	-1.12	0.02487
MIR600HG	9q33.3	-1.12	0.00201
GRAMD2B	NA	-1.11	0.01773
TAPT1-AS1	4p15.32	-1.11	0.01020
AVIL	12q14.1	-1.11	0.00430
PLAG1	8q12	-1.11	0.00329
NUDT16P1	3q22.1	-1.11	0.00222
PANK3	5q34	-1.11	0.02304
AGAP1	2q37	-1.11	0.00124
TSHZ1	18q22.3	-1.11	0.00146
FRG1BP	NA	-1.11	0.00257
ZNF436-AS1	NA	-1.10	0.02247
PPP2R3A	3q22.1	-1.10	0.00605
ABCD3	1p21.3	-1.10	0.01162
SFXN1	NA	-1.10	0.00021
ADCY6	12q12-q13	-1.10	0.00990
LAMB3	1q32	-1.10	0.01183
RITA1	NA	-1.10	0.00799
CASP6	4q25	-1.10	0.03865
HSD17B11	4q22.1	-1.09	0.03253
MIRLET7DHG	9q22.32	-1.09	0.00215
TMEM98	17q11.2	-1.09	0.01361
TNRC6C-AS1	17q25.3	-1.09	0.02074

SORL1	11q23.2-q24.2	-1.09	0.00797
LINC00857	10q22.3	-1.09	0.01005
LINC00324	17p13.1	-1.09	0.00174
MGAT4B	5q35	-1.08	0.04159
OCIAD2	4p11	-1.08	0.04886
C6ORF136	NA	-1.08	0.02814
ZNF717	3p12.3	-1.08	0.00684
CAMK4	5q21.3	-1.08	0.02891
TNK1	17p13.1	-1.08	0.02704
GCNT2	6p24.2	-1.08	0.04439
ZNF607	19q13.1	-1.07	0.00044
HRH1	3p25	-1.07	0.00732
LNX2	13q12.2	-1.07	0.01901
ABTB2	11p13	-1.07	0.03067
CMTM4	16q21-q22.1	-1.07	0.03034
RIMBP2	12q24.33	-1.07	0.01644
ACOX1	17q25.1	-1.07	0.02600
CISD1	10q21.1	-1.07	0.02325
CRAT	9q34.1	-1.07	0.01604
CNDP2	18q22.3	-1.07	0.00269
PLEKHA7	11p15.1	-1.07	0.02160
GCSHP3	NA	-1.07	0.01842
CACNA1A	19p13	-1.06	0.01506
AKAP1	17q22	-1.06	0.02942
LOC220729	3q29	-1.06	0.01102
ACHE	7q22	-1.06	0.02386
ADCY10P1	6p21.1	-1.06	0.01608
SCAMP5	15q24.2	-1.06	0.00123
RNF157	17q25.1	-1.06	0.00054
SECTM1	17q25	-1.05	0.01651
NDFIP2	13q31.1	-1.05	0.01145
GFER	16p13.3-p13.12	-1.05	0.03567
CLCN5	Xp11.23-p11.22	-1.05	0.00464
ZNF175	19q13.4	-1.05	0.00161

TBC1D32	6q22.31	-1.05	0.01555
FAHD1	16p13.3	-1.05	0.03942
GLRX	5q14	-1.04	0.00108
DBP	19q13.3	-1.04	0.01028
TNFRSF14-AS1	NA	-1.04	0.02464
BRI3BP	12q24.31	-1.04	0.00624
PPIP5K1	15q15.3	-1.04	0.03183
FN3K	17q25.3	-1.04	0.02504
DISP1	1q41	-1.04	0.00419
SIGLEC12	19q13.4	-1.04	0.03901
ALDH18A1	10q24.3	-1.04	0.00380
LOC613037	16p11.2	-1.04	0.01181
SH3D21	1p34.3	-1.03	0.03935
CDK8	13q12	-1.03	0.01083
GPD1L	3p22.3	-1.03	0.01039
PDLIM2	8p21.3	-1.03	0.00314
MPI	15q22-qter	-1.03	0.00486
MSMO1	4q32-q34	-1.03	0.04543
PEBP1	12q24.23	-1.03	0.02981
CACNB2	10p12	-1.03	0.02556
PHF7	3p21.1	-1.02	0.00088
PRSS53	16p11.2	-1.02	0.00868
ACAA1	3p22.2	-1.02	0.00571
ING2	4q35.1	-1.02	0.03434
SLC35D1	1p32-p31	-1.02	0.00411
ZNF525	19q13.42	-1.02	0.00411
STAU2	8q21.11	-1.02	0.00080
EMB	5q11.1	-1.01	0.01119
C2CD2L	11q23.3	-1.01	0.00201
BMP8A	1p34.3	-1.01	0.03284
TESK2	1p32	-1.01	0.00266
DNMBP	10q24.2	-1.01	0.03316
RNF207	1p36.31	-1.01	0.04829

LOC202181	5q35.3	-1.01	0.00907
BMX	Xp22.2	-1.01	0.00586
PAFAH2	1p36	-1.00	0.02075
MIER3	5q11.2	-1.00	0.00836
LPAR5	12p13.31	-1.00	0.01519
NAA40	11q13.1	-1.00	0.00038
UCKL1-AS1	20q13.33	-1.00	0.01419
MYH15	3q13.13	-1.00	0.04554
ZNF589	3p21	-1.00	0.01860
ERCC5	13q33	-1.00	0.02378
CRACR2A	NA	-1.00	0.02385
RASA4CP	7p13	-1.00	0.04086
SHROOM1	5q31.1	-1.00	0.01212
CDC42EP4	17q24-q25	-0.99	0.02763
OAS1	12q24.2	-0.99	0.00387
USP18	22q11.21	-0.99	0.02440
KLRB1	12p13	-0.99	0.00519
FZD7	2q33	-0.99	0.02153
EML6	2p16.1	-0.99	0.00925
MOCOS	18q12	-0.99	0.00432
IFT20	17q11.2	-0.99	0.00131
SERINC4	15q15.3	-0.99	0.03313
PMM1	22q13.2	-0.99	0.02304
PLD4	14q32.33	-0.98	0.03340
ZNF813	19q13.42	-0.98	0.03148
PHKA2	Xp22.2-p22.1	-0.98	0.00139
ACRBP	12p13.31	-0.98	0.00284
ALX3	1p13.3	-0.98	0.03220
MFSD9	2q12.1	-0.98	0.00692
HSPA1B	6p21.3	-0.98	0.03915
PGBD2	1q44	-0.98	0.00191
FAM3C	7q31	-0.98	0.01310
ECHDC2	1p32.3	-0.98	0.04618
LOC646762	7p14.3	-0.98	0.00475

ASTE1	3q22.1	-0.97	0.00215
LIPA	10q23.2-q23.3	-0.97	0.02412
AFDN	NA	-0.97	0.00297
DTX1	12q24.13	-0.97	0.04263
PMP22	17p12	-0.97	0.02658
ZNF821	16q22.2	-0.97	0.00978
LSMEM1	7q31.1	-0.97	0.01685
CD244	1q23.3	-0.97	0.01125
SCAPER	15q24	-0.96	0.00073
MYOT	5q31	-0.96	0.03120
HIST1H2AC	6p22.1	-0.96	0.00530
ZNF141	4p16.3	-0.96	0.04338
ZNF443	19p13.2	-0.96	0.00113
MAPK6	15q21	-0.95	0.00756
GPR160	3q26.2-q27	-0.95	0.04072
CYB561D1	1p13.3	-0.95	0.00085
TMEM161B-AS1	NA	-0.95	0.01231
MAGI3	1p12-p11.2	-0.95	0.03984
RGMB	5q15	-0.95	0.02243
DCST2	1q22	-0.95	0.03839
HLA-J	6p21.31	-0.95	0.04820
TCP11L2	12q23.3	-0.94	0.01316
CYP4V2	4q35.2	-0.94	0.01000
SLC25A53	Xq22.2	-0.94	0.00463
DDI2	1p36.21	-0.94	0.01303
ABHD15	17q11.2	-0.94	0.00162
SNX24	5q23.2	-0.94	0.00664
MAPK13	6p21.31	-0.94	0.03668
MIGA2	NA	-0.94	0.00523
LOC728613	5p15.33	-0.94	0.01515
TIGD1	2q37.1	-0.94	0.01881
COX10-AS1	NA	-0.93	0.00384
PEX6	6p21.1	-0.93	0.01836

TOR1AIP2	1q25.2	-0.93	0.04271
ENPP1	6q22-q23	-0.93	0.01672
SAP25	7q22.1	-0.93	0.01938
GNRH1	8p21-p11.2	-0.93	0.00161
LUZP1	1p36	-0.93	0.00463
IGF2BP2	3q27.2	-0.93	0.01318
TMEM150B	19q13.42	-0.93	0.03579
NUDT16	3q22.1	-0.93	0.00148
ZNF844	19p13.2	-0.93	0.00773
ZNF782	9q22.33	-0.93	0.00544
PPP2R5D	6p21.1	-0.93	0.00380
HADHB	2p23	-0.93	0.01587
AIMP2	7p22	-0.92	0.00232
EPB41L3	18p11.32	-0.92	0.00185
AP1AR	4q25	-0.92	0.00514
FAM13A	4q22.1	-0.92	0.03517
NIPA1	15q11.2	-0.92	0.00225
PLEKHA8	7p21-p11.2	-0.92	0.00064
ZNF827	4q31.22	-0.92	0.00905
AKAP9	7q21-q22	-0.92	0.00416
CDK5R1	17q11.2	-0.92	0.02534
PAQR3	4q21.21	-0.92	0.03661
VSIG10L	19q13.41	-0.92	0.02578
SFXN3	10q24.31	-0.92	0.00580
ASB16	17q21.31	-0.91	0.01093
WHRN	NA	-0.91	0.01390
STPG1	1p36.11	-0.91	0.03264
ACVR2A	2q22.3	-0.91	0.02844
C15ORF62	NA	-0.91	0.00078
CD40LG	Xq26	-0.91	0.01783
UCK2	1q23	-0.91	0.00123
NEB	2q22	-0.91	0.03941
DNAJA4	15q25.1	-0.91	0.00605
EIF4EBP2	10q21-q22	-0.91	0.00141

ZNF696	8q24.3	-0.90	0.00280
MAGI1	3p14.1	-0.90	0.01351
C15ORF52	NA	-0.90	0.04817
CD2AP	6p12	-0.90	0.04723
CCNJL	5q33.3	-0.90	0.02745
NLN	5q12.3	-0.90	0.00740
NIPAL2	8q22.2	-0.90	0.03931
ZNF395	8p21.1	-0.90	0.00297
DCAF11	14q11.2	-0.90	0.00765
CDK11A	1p36.33	-0.90	0.03149
ZNF284	19q13.31	-0.89	0.03183
SDHAP1	3q29	-0.89	0.00583
GLS	2q32-q34	-0.89	0.00617
YJEFN3	19p13.11	-0.89	0.02803
LPCAT3	12p13	-0.89	0.02741
ENGASE	17q25.3	-0.89	0.02036
TMED4	7p13	-0.89	0.00941
CNNM4	2q11	-0.89	0.01040
RSRP1	NA	-0.89	0.01183
SOX13	1q32	-0.89	0.03177
PROCA1	17q11.2	-0.89	0.01950
SCP2	1p32	-0.89	0.04698
CFAP44	NA	-0.89	0.00577
RAB11FIP4	17q11.2	-0.89	0.01194
USP30	12q24.11	-0.89	0.00426
ID2	2p25	-0.88	0.00065
MOB3B	9p21.2	-0.88	0.03457
CD96	3q13.13-q13.2	-0.88	0.04678
SLC16A5	17q25.1	-0.88	0.01597
GPR82	Xp11.4	-0.88	0.04697
LIN7B	19q13.3	-0.88	0.02686
INPP4B	4q31.21	-0.88	0.00399
BDH2	4q24	-0.88	0.04510
ACVR1B	12q13	-0.88	0.01736

ADAM1A	12q24.13	-0.88	0.02128
PDP2	16q22.1	-0.87	0.00192
KIFC3	16q13-q21	-0.87	0.01266
ZDHHC9	Xq26.1	-0.87	0.00399
MZF1-AS1	NA	-0.87	0.01428
ZNF701	19q13.41	-0.87	0.00643
NUDT9	4q22.1	-0.87	0.00645
ZNF320	19q13.41	-0.87	0.00596
PDK3	Xp22.11	-0.87	0.00348
ZNF112	NA	-0.87	0.01326
SIDT2	11q23.3	-0.87	0.00998
CTAGE5	14q13.3	-0.87	0.03686
ANO10	3p22.1	-0.87	0.00916
TGFA	2p13	-0.87	0.03077
HMOX1	22q13.1	-0.87	0.01106
GPATCH11	2p22.2	-0.87	0.01422
CAMK2G	10q22	-0.87	0.00779
DNA2	10q21.3-q22.1	-0.87	0.02787
PPARGC1B	5q32	-0.87	0.02453
RMND5A	2p11.2	-0.86	0.00737
DDX11	12p11	-0.86	0.01463
SEMA5A	5p15.2	-0.86	0.01045
SLC39A14	8p21.3	-0.86	0.00503
FNBP1L	1p22.1	-0.86	0.04165
SGK494	17q11.2	-0.86	0.00719
ZSWIM3	20q13.12	-0.86	0.00298
RMND1	6q25.1	-0.86	0.01830
NEDD4	15q	-0.86	0.01572
SRP14-AS1	15q15.1	-0.86	0.03522
TPRG1L	1p36.32	-0.86	0.02928
ERBIN	NA	-0.86	0.02023
KL	13q12	-0.85	0.02524
HECTD3	1p34.1	-0.85	0.01932
RAB11FIP3	16p13.3	-0.85	0.01113

SC5D	11q23.3	-0.85	0.01239
RAB11FIP1	8p11.22	-0.85	0.03170
AIG1	6q24.2	-0.85	0.02715
SHPK	17p13	-0.85	0.03593
PXMP4	20q11.22	-0.85	0.04411
PDCD4	10q24	-0.85	0.01709
ZNF664	12q24.31	-0.85	0.01564
ZNF137P	19q13.4	-0.85	0.01025
TRIM32	9q33.1	-0.85	0.00401
MAP3K9	14q24.2	-0.85	0.00652
RAB37	17q25.1	-0.84	0.02191
NUDT13	10q22.1	-0.84	0.02354
RAD52	12p13-p12.2	-0.84	0.00135
SPATA33	16q24.3	-0.84	0.01560
SUCLA2	13q12.2-q13.3	-0.84	0.00536
MAPKBP1	15q15.1	-0.84	0.01525
ITGA3	17q21.33	-0.84	0.04071
CROT	7q21.1	-0.84	0.03750
ACBD5	10p12.1	-0.84	0.00981
SPATA5L1	15q21.1	-0.84	0.00598
VAV2	9q34.1	-0.84	0.00282
ZNF600	19q13.41	-0.84	0.01054
FLNB	3p14.3	-0.84	0.03367
TCEANC	Xp22.2	-0.83	0.01684
C5ORF63	NA	-0.83	0.04002
SAP30L	5q33.2	-0.83	0.00121
GNG12	1p31.3	-0.83	0.04217
ZFYVE19	15q15.1	-0.83	0.01312
FITM2	20q13.12	-0.83	0.00436
LOC100506100	NA	-0.83	0.00939
NOL4L	NA	-0.83	0.01222
TRMT61B	2p23.2	-0.83	0.00856
MXI1	10q24-q25	-0.83	0.02161
ZNF836	19q13.41	-0.82	0.00375

ARHGAP35	19q13.3	-0.82	0.00250
CACNA2D4	12p13.33	-0.82	0.02625
ANKH	5p15.1	-0.82	0.01053
CMPK2	2p25.2	-0.82	0.03476
ZFP14	19q13.12	-0.82	0.00205
VDR	12q13.11	-0.82	0.02289
ZNF232	17p13.2	-0.82	0.02744
LRRN1	3p26.2	-0.82	0.03194
YBEY	21q22.3	-0.82	0.04230
TSSK3	1p35-p34	-0.82	0.03824
RPS6KA5	14q31-q32.1	-0.82	0.03964
CATSPERG	19q13.1	-0.82	0.00205
FOXO4	Xq13.1	-0.82	0.02034
DGKQ	4p16.3	-0.82	0.02152
ZFP3	17p13.2	-0.82	0.00844
GAREM1	NA	-0.81	0.00852
FGD6	12q22	-0.81	0.02800
B4GALT4	3q13.3	-0.81	0.01880
PLXNA2	1q32.2	-0.81	0.01946
OGG1	3p26.2	-0.81	0.00532
LGALSL	2p14	-0.81	0.02963
LOC286437	Xq22.2	-0.81	0.00886
PPP1R3E	14q11.2	-0.81	0.00241
LRRC28	15q26.3	-0.81	0.02319
ZNF169	9q22.32	-0.81	0.02843
LMBRD2	5p13.2	-0.80	0.02478
ITGAE	17p13	-0.80	0.01111
TRIM52	5q35.3	-0.80	0.03648
SH3D19	4q31.3	-0.80	0.03423
LOC100129917	4p16.3	-0.80	0.01387
VEGFA	6p12	-0.80	0.02022
ZNF818P	19q13.42	-0.80	0.00783
ZFYVE28	4p16.3	-0.80	0.00559
NIT1	1q21-q22	-0.80	0.00332

FAM234B	NA	-0.80	0.00714
ZADH2	18q22.3	-0.80	0.00395
POR	7q11.2	-0.80	0.01607
KIF16B	20p11.23	-0.80	0.03444
PLPP6	NA	-0.80	0.00848
URGCP	7p13	-0.80	0.00911
ZNF768	16p11.2	-0.80	0.00483
ZNF468	19q13.41	-0.80	0.00974
SPRYD7	13q14	-0.80	0.02542
SLC25A43	Xq24	-0.79	0.00431
DTX4	11q12.1	-0.79	0.04485
PLEKHA1	10q26.13	-0.79	0.00894
TSPAN3	15q24.3	-0.79	0.04770
FAHD2A	2q11.2	-0.79	0.01165
CENPJ	13q12.12	-0.79	0.00250
TIRAP	11q24.2	-0.79	0.01071
ATP5SL	19q13.2	-0.79	0.01256
ZC3H12D	6q25.1	-0.79	0.02070
ZNF223	19q13.2	-0.79	0.04392
DHFR	5q11.2-q13.2	-0.79	0.01052
SMAD3	15q22.33	-0.79	0.02088
LRRC8D	1p22.2	-0.79	0.00910
ACOT8	20q13.12	-0.79	0.01350
LYSMD4	15q26.3	-0.79	0.04249
DEPDC1B	5q12.1	-0.79	0.04472
PAPD5	16q12.1	-0.78	0.00321
ZSCAN16	6p22.1	-0.78	0.01117
ALDH5A1	6p22	-0.78	0.03685
VWA5A	11q24.1	-0.78	0.00736
PLIN3	19p13.3	-0.78	0.01532
GSTM3	1p13.3	-0.78	0.03774
SPHK2	19q13.2	-0.78	0.01727
LARGE1	NA	-0.78	0.03652
SAMD10	20q13.33	-0.78	0.01348

SDHAP3	5p15.33	-0.78	0.04465
SESN3	11q21	-0.78	0.04635
C12ORF75	NA	-0.78	0.02320
AUH	9q22.31	-0.77	0.01684
KIAA2013	1p36.22	-0.77	0.02933
FBXO48	2p13.3	-0.77	0.01307
DHRS1	14q12	-0.77	0.03987
PRPF39	14q21.2	-0.77	0.00484
ZNF816	19q13.41	-0.77	0.00863
SRC	20q12-q13	-0.77	0.00959
SAR1B	5q31.1	-0.77	0.02533
SNRNP48	6p24.3	-0.77	0.00554
RAB40C	16p13.3	-0.77	0.02326
PPFIBP2	11p15.4	-0.77	0.00512
AKTIP	16q12.2	-0.77	0.00265
GBA2	9p13.3	-0.77	0.01808
ROGDI	16p13.3	-0.77	0.02393
GLOD4	17p13.3	-0.77	0.01943
PRR5L	11p13-p12	-0.76	0.02636
ORC6	16q12	-0.76	0.04074
RNF121	11q13.4	-0.76	0.00800
TMEM117	12q12	-0.76	0.03239
PXYLP1	NA	-0.76	0.02170
L2HGDH	14q21.3	-0.76	0.03850
DENND1C	19p13.3	-0.76	0.00985
SPAG5	17q11.2	-0.76	0.02036
PLA2G12A	4q25	-0.76	0.01957
RGP1	9pter-p22.1	-0.75	0.02940
BEX2	Xq22	-0.75	0.04737
TTC21A	3p22.2	-0.75	0.02170
IPMK	10q21.1	-0.75	0.02882
CRADD	12q21.33-q23.1	-0.75	0.02930
DPH3	3p25.1	-0.75	0.00701
BRPF3	6p21	-0.75	0.01125

PIGH	14q24.1	-0.75	0.00784
STRIP1	1p13.3	-0.75	0.00536
ZNF213	16p13.3	-0.75	0.00659
KBTBD8	3p14	-0.75	0.00316
ERCC6	10q11.23	-0.75	0.01095
PIGS	17p13.2	-0.74	0.00338
MAMDC4	9q34.3	-0.74	0.01733
LOC730101	6p12.2	-0.74	0.01289
CFDP1	16q22.2-q22.3	-0.74	0.03493
ANKRD23	2q11.2	-0.74	0.01219
EEPD1	7p14.2	-0.74	0.03961
RIOK3	18q11.2	-0.74	0.04542
SNAPC3	9p22.3	-0.74	0.00768
ANKMY2	7p21	-0.74	0.01465
CEP152	15q21.1	-0.74	0.01781
DLL4	15q14	-0.74	0.02405
OXNAD1	3p25-p24	-0.74	0.01915
TLDC2	20q11.23	-0.74	0.02828
RSPH3	6q25.3	-0.74	0.00756
DHCR7	11q13.4	-0.73	0.04149
SEPSECS	4p15.2	-0.73	0.03558
KAT14	NA	-0.73	0.00700
PRTFDC1	10p12.1	-0.73	0.01027
SLC25A51	9p13.3-p12	-0.73	0.01432
BLNK	10q23.2-q23.33	-0.73	0.04229
ZNF124	1q44	-0.73	0.03707
ACOT2	14q24.3	-0.73	0.03897
ZHX3	20q12	-0.73	0.01534
NBAS	2p24	-0.72	0.00741
NCKIPSD	3p21	-0.72	0.01531
GPAM	10q25.2	-0.72	0.01773
SARM1	17q11	-0.72	0.01621
CAT	11p13	-0.72	0.03419
DGKD	2q37.1	-0.72	0.03193

ZNF420	19q13.12	-0.72	0.01591
TBC1D13	9q34.11	-0.72	0.00232
ZDHHC21	9p22.3	-0.72	0.02044
ZNF81	Xp11.23	-0.72	0.02822
PPID	4q31.3	-0.71	0.01198
POLE	12q24.3	-0.71	0.00241
CEACAM19	19q13.31	-0.71	0.01621
MRI1	19p13.2	-0.71	0.02465
HAGH	16p13.3	-0.71	0.02414
ALAD	9q33.1	-0.71	0.02261
SMYD4	17p13.3	-0.71	0.00643
MYO19	17q12	-0.71	0.00785
GUSBP11	22q11.23	-0.71	0.01240
COQ9	16q21	-0.71	0.04200
ZNF613	19q13.41	-0.71	0.01069
ZNF799	19p13.2	-0.71	0.01366
MLX	17q21.1	-0.71	0.00960
ZNF620	3p22.1	-0.71	0.01844
ZNF473	19q13.33	-0.70	0.00332
COX19	7p22.3	-0.70	0.03608
COQ6	14q24.3	-0.70	0.01512
DENND1B	1q31.3	-0.70	0.03896
ZNF507	19q13.11	-0.70	0.00746
HINFP	11q23.3	-0.70	0.00345
FASTKD3	5p15.31	-0.70	0.02003
IRF5	7q32	-0.70	0.04919
SMAD7	18q21.1	-0.70	0.03599
FAM213B	1p36.32	-0.70	0.03022
GMCL1	2p13.3	-0.70	0.04523
SRD5A1	5p15	-0.70	0.03640
MTRF1L	6q25-q26	-0.69	0.00737
TIGD2	4q22.1	-0.69	0.04074
PAXBP1-AS1	NA	-0.69	0.03163
ZNF253	19p13.11	-0.69	0.00680

TARP	7p15-p14	-0.69	0.04044
PPP1R26	9q34.3	-0.69	0.01179
LBX2-AS1	2p13.1	-0.69	0.02808
LSM11	5q33.3	-0.69	0.04286
OFD1	Xp22	-0.69	0.04187
DENND2D	1p13.3	-0.69	0.01222
TFAP4	16p13	-0.69	0.02657
TRIM47	17q25	-0.69	0.00757
KRI1	19p13.2	-0.69	0.02393
PAOX	10q26.3	-0.69	0.03962
BLCAP	20q11.23	-0.69	0.01257
BRICD5	16p13.3	-0.69	0.01579
CCDC127	5p15.33	-0.69	0.00623
ZNF564	19p13.2	-0.69	0.02525
TPT1-AS1	13q14.13	-0.68	0.02576
RPS6KA1	1p	-0.68	0.03986
BTBD9	6p21	-0.68	0.00691
BHLHB9	Xq23	-0.68	0.03408
GSG2	17p13	-0.68	0.04707
ZFP62	5q35.3	-0.68	0.01522
SAMD1	19p13.12	-0.68	0.00908
DDHD2	8p11.23	-0.68	0.02911
TNFRSF25	1p36.2	-0.68	0.00599
ALOX12	17p13.1	-0.68	0.04335
ZNF292	6q14.3	-0.68	0.03653
RTL10	NA	-0.67	0.00792
ZDHHC6	10q25.2	-0.67	0.00954
SLC25A42	19p13.11	-0.67	0.01409
CMTR2	16q22.2	-0.67	0.02576
ACOT13	6p22.3	-0.67	0.01164
DYNLL2	17q22	-0.67	0.02879
ZNF596	8p23.3	-0.67	0.01558
NT5DC1	6q22.1	-0.67	0.02155
NIPAL3	1p36.12-p35.1	-0.67	0.01080

RABL2A	2q13	-0.67	0.01438
SLC35E2B	1p36.33	-0.67	0.03250
KYAT1	NA	-0.67	0.00198
PHRF1	11p15.5	-0.67	0.00948
ANKRD46	8q22.2	-0.67	0.01838
CACNA2D2	3p21.3	-0.67	0.04689
LHX4-AS1	NA	-0.67	0.02026
SGPP2	2q36.1	-0.67	0.03516
TOB1	17q21	-0.66	0.03472
KBTBD7	13q14.11	-0.66	0.02571
ZNF616	19q13.41	-0.66	0.01009
SFI1	22q12.2	-0.66	0.02629
C11ORF24	NA	-0.66	0.03650
DENND5B	12p11.21	-0.66	0.04501
ZNF181	19q13.11	-0.66	0.01217
SFXN5	NA	-0.66	0.00900
ZNF577	19q13.41	-0.66	0.02300
WDR60	7q36.3	-0.65	0.02031
PLPBP	NA	-0.65	0.00691
KLHL8	4q22.1	-0.65	0.03610
CCDC93	2q14.1	-0.65	0.03300
LINC00667	18p11.31	-0.65	0.01691
PEX14	1p36.22	-0.65	0.01624
IGSF8	1q23.1	-0.65	0.01254
NAGPA	16p13.3	-0.65	0.01970
ZNF526	19q13.2	-0.65	0.00998
GALT	9p13	-0.65	0.01441
CD82	11p11.2	-0.65	0.03955
INSR	19p13.3-p13.2	-0.65	0.01600
NEK8	17q11.1	-0.65	0.04766
GATS	7q22.1	-0.65	0.03728
CEP192	18p11.21	-0.65	0.00518
ZNF480	19q13.41	-0.65	0.02389
PCYT1A	3q29	-0.64	0.00835

FGFR1OP	6q27	-0.64	0.01589
ING1	13q34	-0.64	0.03564
DGKA	12q13.3	-0.64	0.04236
ZCCHC14	16q24.2	-0.64	0.01651
MAIP1	NA	-0.64	0.04175
GPALPP1	13q14.12	-0.64	0.02392
SLC9A6	Xq26.3	-0.64	0.01377
RIPK3	14q11.2	-0.64	0.01514
SURF6	9q34.2	-0.64	0.03224
THAP2	12q21.1	-0.63	0.00815
ZNF862	7q36.1	-0.63	0.03529
ZFYVE27	10q24.2	-0.63	0.01168
TTC19	17p12	-0.63	0.03490
CELF6	15q24	-0.63	0.01274
COX10	17p12	-0.63	0.02533
ZNF30	19q13.11	-0.63	0.03324
CLCN6	1p36	-0.63	0.01631
PNPLA2	11p15.5	-0.62	0.03584
ZSWIM1	20q13.12	-0.62	0.02454
GCLM	1p22.1	-0.62	0.04735
PHYKPL	5q35.3	-0.62	0.01832
ZNF606	19q13.4	-0.62	0.02702
ZNF619	3p22.1	-0.62	0.04178
FLCN	17p11.2	-0.62	0.02067
FASTKD1	2q31	-0.62	0.03955
CLOCK	4q12	-0.62	0.02732
BAHD1	15q15.1	-0.62	0.02157
SLC26A6	3p21.3	-0.62	0.01067
C9ORF64	NA	-0.62	0.03205
NFS1	20q11.22	-0.61	0.02443
SH3BP5L	1q44	-0.61	0.02052
ZNF785	16p11.2	-0.61	0.02074
TRAK1	3p22.1	-0.61	0.04490
LYSMD1	1q21.3	-0.61	0.01358

PEX12	17q12	-0.61	0.02579
PGAP2	11p15.5	-0.61	0.02703
ZFP41	8q24.3	-0.61	0.01639
TUBGCP4	15q15	-0.60	0.02745
TMEM79	1q22	-0.60	0.02048
RFC5	12q24.23	-0.60	0.00638
RAD9A	11q13.1-q13.2	-0.60	0.00577
CEP76	18p11.21	-0.60	0.03535
MYH7B	20q11.22	-0.60	0.02883
ZNF594	17p13	-0.60	0.03618
CIPC	NA	-0.60	0.03423
C14ORF1	NA	-0.60	0.04993
SIRT7	17q25	-0.60	0.04424
UBA6-AS1	4q13.2	-0.60	0.04115
ACSF3	16q24.3	-0.60	0.02826
TAF5	10q24-q25.2	-0.60	0.01710
ZNF780A	19q13.2	-0.60	0.03573
LMNTD2	NA	-0.60	0.04502
CBFA2T2	20q11	-0.60	0.02880
TOP3B	22q11.22	-0.60	0.02146
ZNF211	19q13.4	-0.59	0.04612
LRRC40	1p31.1	-0.59	0.04648
STRBP	9q33.3	-0.59	0.04474
ZKSCAN4	6p21	-0.59	0.03397
TLDC1	16q24.1	-0.59	0.02900
ZNF763	19p13.2	-0.59	0.04399
BORA	13q22.1	-0.59	0.02635
TMEM80	11p15.5	-0.59	0.01607
FZD6	8q22.3-q23.1	0.59	0.02296
CASC4	15q15.3	0.59	0.04560
JAML	NA	0.59	0.02839
APBB2	4p13	0.59	0.02318
ADNP2	18q23	0.59	0.03245
REXO2	11q23.2	0.59	0.04613

GNL3	3p21.1	0.59	0.02474
DNAL1	14q24.3	0.60	0.00661
MKL1	22q13	0.60	0.03472
BTF3L4	1p32.3	0.60	0.01925
C18ORF54	NA	0.60	0.03865
ZNF542P	NA	0.60	0.03670
YARS	1p35.1	0.60	0.03284
IQCK	16p12.3	0.60	0.01534
PNMA1	14q24.3	0.60	0.03955
SOX4	6p22.3	0.60	0.01014
SLC9A5	16q22.1	0.60	0.03104
LINC00998	7q31.1	0.61	0.01093
SLC39A13	11p11.2	0.61	0.04278
ICA1L	2q33.2	0.61	0.04214
STK40	1p34.3	0.61	0.02568
SLC25A25	9q34.11	0.61	0.04001
OSGIN2	8q21	0.61	0.03629
RNF215	22q12.2	0.61	0.02489
PRKD3	2p21	0.61	0.03275
LPAR6	13q14	0.61	0.01468
CNTLN	9p22.2	0.61	0.03464
CBFB	16q22.1	0.61	0.02376
MARK4	19q13.3	0.62	0.04521
TMEM123	11q22.1	0.62	0.03766
IPW	15q11-q12	0.62	0.03404
TANGO6	16q22.1	0.62	0.01010
KCTD1	18q11.2	0.63	0.04938
S1PR2	19p13.2	0.63	0.03670
NBDY	NA	0.63	0.03189
CTNNAL1	9q31.2	0.63	0.02881
AMZ2P1	17q24.1	0.63	0.02747
CYGB	17q25	0.63	0.01381
FGD5	3p25.1	0.63	0.02482
MAGEF1	3q13	0.63	0.03579

RASAL2	1q24	0.64	0.03603
SLC18B1	6q22.3-q23.3	0.64	0.01496
GPM6B	Xp22.2	0.64	0.04361
DNAJC1	10p12.31	0.64	0.01678
MESDC1	15q13	0.64	0.04320
PDE2A	11q13.4	0.64	0.04132
TMEM65	8q24.13	0.64	0.03117
C1ORF56	NA	0.64	0.04810
COMMD7	20q11.21	0.64	0.04447
ARPP19	15q21.2	0.65	0.02472
FAM86B3P	8p23.1	0.65	0.02328
BCAP29	7q22.3	0.65	0.01354
RHBDD1	2q36.3	0.65	0.02834
STAB1	3p21.1	0.65	0.04085
ANKDD1A	15q22.31	0.65	0.03316
STAC3	12q13.3	0.65	0.04691
TMED9	5q35.3	0.65	0.04454
ARHGEF40	14q11.2	0.65	0.04459
SLC7A1	13q12.3	0.65	0.04397
RHBDF1	16p13.3	0.65	0.01362
ADAM9	8p11.22	0.65	0.04602
TMEM165	4q12	0.66	0.02945
MFSD14A	NA	0.66	0.02403
JAZF1	7p15.2-p15.1	0.66	0.03608
FAM214B	9p13.3	0.66	0.01076
VOPP1	7p11.2	0.66	0.03456
AHI1	6q23.3	0.66	0.01058
CERS5	12q13.12	0.66	0.02873
ZDHHC2	8p22	0.66	0.04854
JAM3	11q25	0.66	0.04879
SNTB2	16q22.1	0.66	0.04660
B3GNTL1	17q25.3	0.66	0.01201
EXTL3	8p21	0.67	0.01624
UAP1L1	9q34.3	0.67	0.02297

BACE1	11q23.2-q23.3	0.67	0.03486
RIN3	14q32.12	0.67	0.00754
UTP11	NA	0.67	0.01958
EPB41L4A	5q21.3	0.67	0.03904
RBPJ	4p15.2	0.67	0.01792
TLE4	9q21.31	0.67	0.00874
ATG7	3p25.3	0.67	0.01836
TMEM67	8q22.1	0.68	0.04215
PDIA4	7q35	0.68	0.02957
PLK2	5q12.1-q13.2	0.68	0.03314
CASD1	7q21.3	0.68	0.03499
LOC90784	2p11.2	0.68	0.04437
WFS1	4p16.1	0.68	0.04753
POLB	8p11.2	0.69	0.02137
CADPS2	7q31.3	0.69	0.01985
QPCTL	19q13.32	0.69	0.04827
SGCE	7q21.3	0.69	0.02184
UNC13D	17q25.1	0.69	0.03161
TMEM263	NA	0.69	0.01424
UTRN	6q24	0.69	0.02483
SPATA7	14q31.3	0.69	0.03517
MEF2D	1q12-q23	0.69	0.01635
PLXDC2	10p12.31	0.69	0.04992
FAM102B	1p13.3	0.70	0.01743
KDELC1	13q33	0.70	0.04055
TCAF2	NA	0.70	0.03097
SGCB	4q12	0.70	0.02393
MPZ	1q23.3	0.70	0.01293
GAA	17q25.2-q25.3	0.70	0.02000
RRN3P2	16p11.2	0.70	0.00357
BYSL	6p21.1	0.70	0.01026
EVA1C	21q22.11	0.70	0.04787
PTGER4	5p13.1	0.70	0.01758
C1ORF216	NA	0.70	0.02337

RNF130	5q35.3	0.70	0.02591
FKBP14	7p14.3	0.70	0.03308
PNO1	2p14	0.70	0.02142
TXNDC15	5q31.1	0.71	0.04318
SPATS2	12q13.12	0.71	0.00862
SLC1A5	19q13.3	0.71	0.03944
PPRC1	10q24.32	0.71	0.02349
APOBEC3F	22q13.1	0.71	0.04934
ZNF628	19q13.42	0.71	0.02781
DTWD1	15q21.2	0.71	0.02951
MANBA	4q22-q25	0.72	0.01784
CNRIP1	2p14	0.72	0.01879
BAG3	10q25.2-q26.2	0.72	0.01057
COTL1	16q24.1	0.72	0.02648
TPBG	6q14-q15	0.72	0.01355
C12ORF45	NA	0.72	0.04860
RNF180	5q12.3	0.72	0.00652
RHOBTB3	5q15	0.72	0.03491
TM6SF1	15q24-q26	0.72	0.03194
TULP3	12p13.3	0.72	0.03391
TSEN15	1q25	0.73	0.01619
ARMCX2	Xq21.33-q22.2	0.73	0.02492
GYG1	3q24-q25.1	0.73	0.02295
LINC-PINT	NA	0.73	0.03930
FAHD2B	2q11.2	0.73	0.02852
GPSM1	9q34.3	0.73	0.04262
CSNK1E	22q13.1	0.73	0.01274
HABP4	9q22.3-q31	0.73	0.01838
TCTN1	12q24.11	0.74	0.02114
DNMT3B	20q11.2	0.74	0.00988
MAPK10	4q22.1-q23	0.74	0.04163
SPTSSA	14q13.1	0.74	0.00963
C9ORF3	NA	0.74	0.00406
SH3PXD2A	10q24.33	0.74	0.03943

ERC1	12p13.3	0.75	0.02687
CREG1	1q24	0.75	0.03088
GTF2IRD1	7q11.23	0.75	0.02168
ALCAM	3q13.1	0.75	0.03509
LPCAT1	5p15.33	0.75	0.01605
KDELC2	11q22.3	0.75	0.02320
TBC1D19	4p15.2	0.75	0.00768
LRP11	6q25.1	0.75	0.02457
RNASE1	14q11.2	0.75	0.02542
LRRC8B	1p22.2	0.75	0.02557
FRY	13q13.1	0.75	0.02369
CRTC3	15q26.1	0.75	0.04100
TMEM184B	22q12	0.75	0.01259
RASSF3	12q14.2	0.75	0.00313
RAB13	1q21.2	0.75	0.01591
WLS	1p31.3	0.76	0.02335
HCST	19q13.1	0.76	0.04189
MAP9	4q32.1	0.76	0.03159
DLG4	17p13.1	0.77	0.02312
KANK3	19p13.2	0.77	0.03888
SLC22A17	14q11.2	0.77	0.01626
ST6GALNAC6	9q34.11	0.77	0.01002
COL23A1	5q35.3	0.77	0.03614
SLC25A32	8q22.3	0.77	0.01058
ALOX5AP	13q12	0.77	0.04789
RAP2B	3q25.2	0.77	0.00345
ABLIM3	5q32	0.77	0.02643
PML	15q22	0.77	0.01650
TNFRSF1B	1p36.22	0.78	0.04409
AAED1	9q22.32	0.78	0.01172
SIX5	19q13.32	0.78	0.02695
NETO2	16q11	0.78	0.02345
KLHL5	4p14	0.78	0.04489
MIER2	19p13.3	0.78	0.00250

TBKBP1	17q21.32	0.78	0.00868
CUEDC1	17q23.2	0.78	0.00116
TNFSF12	17p13	0.78	0.02742
KLF11	2p25	0.78	0.00876
BID	22q11.1	0.79	0.01932
TGFBR2	3p22	0.79	0.02732
NBN	8q21	0.79	0.04067
LGALS9	17q11.2	0.79	0.01150
EBF4	20p13	0.79	0.01082
FGL2	7q11.23	0.79	0.02923
GOLT1B	12p12.1	0.79	0.00813
CCDC8	19q13.32	0.79	0.03918
THBS3	1q21	0.79	0.01399
IL6ST	5q11.2	0.79	0.02252
CHST12	7p22	0.79	0.02688
IL15RA	10p15.1	0.79	0.02675
MOB1B	4q13.3	0.79	0.01148
TGIF1	18p11.3	0.79	0.00348
MTMR6	13q12	0.79	0.01448
TRPC6	11q22.1	0.79	0.02840
SEZ6L2	16p11.2	0.79	0.01045
TCF7L1	2p11.2	0.79	0.01666
DSTN	20p12.1	0.80	0.02487
UBE2D1	10q21.1	0.80	0.03797
CD33	19q13.3	0.80	0.02019
STK38L	12p11.23	0.80	0.04564
HSPA4L	4q28	0.80	0.02984
MARCKS	6q22.2	0.80	0.02532
NHS	Xp22.13	0.80	0.01782
LINC00641	14q11.2	0.80	0.04210
COL9A3	20q13.3	0.80	0.04693
CD63	12q12-q13	0.80	0.01576
PSAP	10q21-q22	0.80	0.03962
ESR2	14q23.2	0.80	0.04062

DOK2	8p21.3	0.80	0.02331
OLFML1	11p15.4	0.80	0.02973
SH2B3	12q24	0.81	0.03617
TRIB2	2p24.3	0.81	0.01162
ZNF275	Xq28	0.81	0.00692
CTSF	11q13	0.81	0.03254
ZC2HC1A	8q21.12	0.81	0.00700
MAGED1	Xp11.23	0.81	0.00404
UBE2L6	11q12	0.81	0.03510
PTPRS	19p13.3	0.81	0.00313
SKA2	17q22	0.81	0.01926
ITGAV	2q31-q32	0.81	0.04296
DPY19L1	7p14.3-p14.2	0.81	0.01610
TMEM55A	8q21.3	0.81	0.03410
RARG	12q13	0.81	0.03373
SELENON	NA	0.82	0.00298
C15ORF39	NA	0.82	0.01453
NCDN	1p34.3	0.82	0.02446
ZNF449	Xq26.3	0.82	0.01854
LRP3	19q13.11	0.82	0.00904
TACC1	8p11.22	0.82	0.02510
CSPG4	15q24.2	0.82	0.04640
TMEM268	NA	0.82	0.00631
LDHB	12p12.2-p12.1	0.82	0.03442
RSU1	10p13	0.82	0.00918
NFIX	19p13.3	0.82	0.03929
IFT22	NA	0.82	0.00925
MS4A6A	11q12.1	0.83	0.02948
PKN1	19p13.12	0.83	0.01957
MBNL2	13q32.1	0.83	0.00493
TSC22D2	3q25.1	0.83	0.00598
LAMB2	3p21	0.83	0.02921
TCIRG1	11q13.2	0.83	0.02148
MDFIC	7q31.1-q31.2	0.83	0.03045

KLF9	9q13	0.83	0.04013
TMEM71	8q24.22	0.84	0.02192
PACS1	11q13.1-q13.2	0.84	0.02267
EFS	14q11.2-q12	0.84	0.04348
ADPRH	3q13.31-q13.33	0.84	0.03845
HOMER3	19p13.11	0.84	0.02866
TNFRSF12A	16p13.3	0.84	0.03420
TEAD2	19q13.3	0.84	0.01820
PTBP2	1p21.3	0.84	0.00483
GRK5	10q26.11	0.84	0.00231
SHROOM2	Xp22.3	0.84	0.02461
HK1	10q22	0.84	0.03391
ST5	11p15	0.84	0.00371
PDGFRA	4q12	0.84	0.02556
PLVAP	19p13.2	0.84	0.02334
ZNF667-AS1	19q13.43	0.84	0.02208
SPCS3	4q34.2	0.84	0.02745
PBX3	9q33.3	0.85	0.01891
RRP12	10q24.1	0.85	0.02247
RILPL1	12q24.31	0.85	0.03741
CDK17	12q23.1	0.85	0.02314
FZD8	10p11.21	0.85	0.00439
CARD6	5p13.1	0.85	0.00351
WIPI1	17q24.2	0.85	0.01949
FLT1	13q12	0.85	0.04910
DPY19L2	12q14.2	0.85	0.01394
ARHGAP20	11q23.1	0.85	0.00921
TMEM237	2q33.2	0.85	0.01805
PAM	5q14-q21	0.86	0.01677
CMTM3	16q21	0.86	0.00606
STK26	NA	0.86	0.00530
TAL1	1p32	0.86	0.00178
USP13	3q26.2-q26.3	0.86	0.02243
ACOT9	Xp22.11	0.86	0.00462

SMTN	22q12.2	0.86	0.01861
RUNX1T1	8q22	0.86	0.00879
CD300A	17q25.1	0.87	0.02223
CHRD	3q27	0.87	0.04004
ZMAT3	3q26.32	0.87	0.02668
MLF1	3q25.1	0.87	0.00552
IMPDH1	7q31.3-q32	0.87	0.03261
PYGM	11q12-q13.2	0.87	0.02763
PHTF1	1p13	0.87	0.00688
RTL8C	NA	0.88	0.00719
UBE2Q2	15q24.2	0.88	0.00685
BEX3	NA	0.88	0.01842
ZDHHC1	16q22.1	0.88	0.00428
CCDC69	5q33.1	0.88	0.03543
RUFY4	2q35	0.88	0.04440
ZFP36	19q13.1	0.88	0.04242
AJUBA	14q11.2	0.88	0.00527
TRPA1	8q13	0.88	0.02006
C9ORF72	NA	0.88	0.02447
CAV2	7q31.1	0.88	0.00923
YBX3	12p13.1	0.88	0.00653
BBS5	2q31.1	0.89	0.00458
MORN2	2p22.1	0.89	0.01857
TEAD1	11p15.2	0.89	0.01608
HSF4	16q21	0.89	0.00622
RGS2	1q31	0.89	0.02520
EPHB4	7q22	0.89	0.01190
MEF2C	5q14.3	0.89	0.01597
VMP1	17q23.1	0.89	0.00170
RNF122	8p12	0.89	0.01907
PPM1H	12q14.1	0.89	0.00465
FAM69A	1p22.1	0.89	0.00119
ADA	20q13.12	0.90	0.03443
DUBR	NA	0.90	0.01580

C7ORF50	NA	0.90	0.04208
TPST2	22q12.1	0.90	0.01498
LAP3	4p15.32	0.90	0.03681
SIL1	5q31	0.90	0.04376
JAK2	9p24	0.90	0.01327
SLC12A4	16q22.1	0.90	0.03394
P3H3	NA	0.90	0.01076
DCBLD2	3;3q12.1	0.90	0.00558
NT5DC2	3p21.1	0.90	0.04628
TSPYL5	8q22.1	0.90	0.00743
H1FX	3q21.3	0.90	0.03707
TMTC2	12q21.31	0.90	0.04989
ETS2	21q22.2	0.91	0.02471
KIAA0754	1p34.3	0.91	0.01702
CYTIP	2q11.2	0.91	0.04000
TMEM86A	11p15.1	0.91	0.03377
PARVB	22q13.2-q13.33	0.91	0.02860
IQSEC1	3p25.2	0.91	0.03815
ADAP2	17q11.2	0.91	0.01486
HDGFL3	NA	0.91	0.02525
LMO2	11p13	0.91	0.01799
MTMR9LP	1p35.1	0.91	0.00594
MXRA8	1p36.33	0.91	0.02116
ACVRL1	12q13.13	0.91	0.00091
PLEKHA4	19q13.33	0.91	0.01016
ITIH5	10p14	0.91	0.03978
R3HDM4	19p13.3	0.92	0.00990
DIXDC1	11q23.1	0.92	0.03955
ZNF532	18q21.32	0.92	0.04867
MAP7D3	Xq26.3	0.92	0.00717
ZNF438	10p11.23	0.92	0.01366
HCP5	6p21.3	0.92	0.03492
SPIRE1	18p11.21	0.92	0.01296
PHC2	1p34.3	0.92	0.00288

NFIA	1p31.3-p31.2	0.93	0.03178
CDC25B	20p13	0.93	0.01050
LCAT	16q22.1	0.93	0.00443
SIGLEC7	19q13.3	0.93	0.01590
ACVR1	2q23-q24	0.93	0.00320
JAM2	21q21.2	0.93	0.00657
CMTM7	3p22.3	0.93	0.02806
CRTAP	3p22.3	0.93	0.00192
NR2F1-AS1	5q15	0.93	0.01631
MMD	17q	0.94	0.00377
HLA-DPB1	6p21.3	0.94	0.01187
TLE1	9q21.32	0.94	0.00687
IFITM1	11p15.5	0.94	0.03119
CDC42EP3	2p21	0.94	0.01643
STX2	12q24.33	0.94	0.01434
TLR5	1q41-q42	0.94	0.01510
AFAP1L1	5q32	0.94	0.01544
ARRDC3	5q14.3	0.94	0.03947
CARD16	NA	0.94	0.03420
LAPTM5	1p34	0.94	0.04724
SERPINB8	18q22.1	0.94	0.00108
CTSC	11q14.2	0.94	0.02074
NES	1q23.1	0.94	0.00215
KANK2	19p13.2	0.95	0.04417
CLIP3	19q13.12	0.95	0.02564
IRS2	13q34	0.95	0.01214
SASH1	6q24.3	0.95	0.03050
SLC27A1	19p13.11	0.95	0.00160
MCRIP1	NA	0.95	0.02358
SCN8A	12q13	0.95	0.04585
PLD3	19q13.2	0.95	0.00390
TSPAN9	12p13.33-p13.32	0.95	0.03676
DPYSL2	8p22-p21	0.95	0.00711
CPVL	7p15.1	0.95	0.01388

ANXA3	4q21.21	0.95	0.00411
MAPKAPK2	1q32	0.95	0.00403
SERPINH1	11q13.5	0.95	0.04150
SLC16A14	2q36.3	0.95	0.00452
C1QTNF1	17q25.3	0.95	0.03588
ENC1	5q13	0.95	0.00175
NCALD	8q22.2	0.95	0.01363
RASGRP3	2p25.1-p24.1	0.95	0.04915
TGFBR3	1p33-p32	0.96	0.01249
CRIM1	2p21	0.96	0.02187
ZFPM2	8q23	0.96	0.03251
TGFB1	19q13.1	0.96	0.04591
FAM92A	NA	0.96	0.00196
HSD17B14	19q13.33	0.96	0.02004
PCBP3	21q22.3	0.96	0.04564
ASNS	7q21.3	0.96	0.03048
SLFN11	17q12	0.96	0.02646
EDNRB	13q22	0.96	0.02002
ZNF185	Xq28	0.97	0.01207
ITPRIPL2	16p12.3	0.97	0.00260
SFMBT2	10p14	0.97	0.02417
GLIPR2	9p13.3	0.97	0.02407
EIF2AK4	15q15.1	0.97	0.01135
DTNB	2p24	0.97	0.02666
LINC00963	NA	0.97	0.00032
KATNAL1	13q12.3	0.97	0.01297
CMTM6	3p22.3	0.97	0.01939
ADGRE5	NA	0.97	0.00806
TEK	9p21	0.97	0.00983
PRNP	20p13	0.97	0.01336
HDAC7	12q13.1	0.97	0.01135
AOAH	7p14-p12	0.97	0.02531
GALNT18	11p15.3	0.98	0.03224
FMNL2	2q23.3	0.98	0.03537

GLCCI1	7p21.3	0.98	0.00466
APOL3	22q13.1	0.98	0.04432
TFPI	2q32	0.98	0.00746
SAMD14	17q21.33	0.98	0.00551
DNASE2	19p13.2	0.98	0.00700
SH3BGRL3	1p36.11	0.98	0.02323
ANXA6	5q33.1	0.98	0.01600
STRIP2	7q32.1	0.98	0.03110
RAPGEF4	2q31-q32	0.98	0.02230
SLC22A15	1p13.1	0.98	0.01486
SLFN12	17q12	0.98	0.00370
CC2D2A	4p15.32	0.99	0.00174
KLHDC8B	3p21.31	0.99	0.00059
TGIF2	20q11.23	0.99	0.00358
UCP2	11q13	0.99	0.02087
EIF5A2	3q26.2	0.99	0.00134
GPR155	2q31.1	0.99	0.00651
ISG20	15q26	0.99	0.01847
SH3BP4	2q37.1-q37.2	0.99	0.00472
TDRD6	6p12.3	0.99	0.02341
SLC15A3	11q12.2	0.99	0.04859
ERLEC1	2p16.2	0.99	0.00694
SHROOM4	Xp11.22	0.99	0.02723
PRKAR2B	7q22	0.99	0.01409
ZEB1	10p11.2	0.99	0.01351
RASGRF2	5q13	0.99	0.00210
PLPPR2	NA	0.99	0.00566
HM13	20q11.21	0.99	0.02973
COL16A1	1p35-p34	0.99	0.00536
PIM1	6p21.2	0.99	0.00498
LMNA	1q22	0.99	0.00374
SMIM10	Xq26.3	1.00	0.00226
AXL	19q13.1	1.00	0.00309
NME4	16p13.3	1.00	0.01599

RHOQ	2p21	1.00	0.02006
SMARCA1	Xq25	1.00	0.01178
SRF	6p21.1	1.00	0.00436
OGFRL1	6q13	1.00	0.01217
ZNF423	16q12	1.00	0.01084
DNM3	1q24.3	1.00	0.00569
GM2A	5q33.1	1.00	0.03627
COX7A1	19q13.1	1.00	0.04853
SSR3	3q25.31	1.00	0.01736
PARVA	11p15.3	1.00	0.00265
VAMP5	2p11.2	1.00	0.04036
PLIN4	19p13.3	1.00	0.04607
LOX	5q23.2	1.00	0.03301
EXTL2	1p21	1.00	0.00157
ENTPD1	10q24	1.00	0.04023
CRYAB	11q22.3-q23.1	1.01	0.04508
PHKA1	Xq12-q13	1.01	0.02077
MEIS3P1	17p12	1.01	0.00581
PYCR1	17q25.3	1.01	0.02764
NFIC	19p13.3	1.01	0.00218
MYO1F	19p13.3-p13.2	1.01	0.03882
AEN	15q26.1	1.01	0.00635
SMAP2	1p35.3-p34.1	1.02	0.02319
SOBP	6q21	1.02	0.00338
AMIGO2	12q13.11	1.02	0.03821
PPT1	1p32	1.02	0.01098
TCEAL4	Xq22.2	1.02	0.00412
CEBPD	8p11.2-p11.1	1.02	0.03241
PMEPA1	20q13.31-q13.33	1.02	0.00278
AP1S2	Xp22.2	1.02	0.00753
ASAP1	8q24.1-q24.2	1.02	0.02892
TUSC3	8p22	1.02	0.00444
NUGGC	8p21.1	1.02	0.01989
CLEC11A	19q13.3	1.02	0.00338

IL18R1	2q12	1.02	0.01550
IFT81	12q24.13	1.02	0.00255
GDNF	5p13.1-p12	1.02	0.03719
MPZL2	11q24	1.02	0.00289
F2RL3	19p12	1.02	0.02896
C1ORF54	NA	1.03	0.00085
SNHG16	NA	1.03	0.03976
AKR1B1	7q35	1.03	0.01318
FKBP7	2q31.2	1.03	0.00081
CORO1C	12q24.1	1.03	0.00157
AIF1L	9q34.13-q34.3	1.03	0.01085
ABCC1	16p13.1	1.03	0.00174
SLC2A1	1p34.2	1.03	0.00974
SLC9A7	Xp11.3	1.03	0.03921
MAP4K4	2q11.2-q12	1.03	0.02342
CTSS	1q21	1.03	0.01345
FXYD5	19q13.12	1.03	0.04823
FOXF1	16q24	1.03	0.00242
FLJ23867	1q25.2	1.03	0.01830
MAMLD1	Xq28	1.04	0.00116
PRAM1	19p13.2	1.04	0.01128
DSE	6q22	1.04	0.03433
CAB39L	13q14.2	1.04	0.00086
SPACA6	NA	1.04	0.02905
MSANTD3	9q31.1	1.04	0.00927
JUNB	19p13.2	1.04	0.02188
NOTCH2NL	1q21.2	1.04	0.00409
C1QTNF3	5p13	1.04	0.04374
TCF4	18q21.1	1.05	0.02514
MARVELD1	10q24.2	1.05	0.00549
PLEKHO2	15q22.1	1.05	0.02758
NPR1	1q21-q22	1.05	0.01734
E2F5	8q21.2	1.05	0.01196
KIF26A	14q32.33	1.05	0.02657

CLEC2B	12p13-p12	1.05	0.02320
C1QC	1p36.11	1.05	0.02495
BATF2	11q13.1	1.05	0.04177
SRM	1p36-p22	1.05	0.03902
RILPL2	12q24.31	1.06	0.03357
PRKAR1A	17q23-q24	1.06	0.00588
KCTD10	12q24.11	1.06	0.00306
TBXAS1	7q34-q35	1.06	0.00383
RGS18	1q31.2	1.06	0.01147
TGFB3	14q24	1.06	0.04711
ROBO1	3p12	1.06	0.02414
OLFML2A	9q33.3	1.06	0.01139
KDR	4q11-q12	1.06	0.00750
TRIM16	17p11.2	1.06	0.02321
ISLR	15q23-q24	1.06	0.03067
HYAL2	3p21.3	1.06	0.01578
MSX2	5q35.2	1.06	0.01778
PHYHD1	9q34.11	1.06	0.00158
UPP1	7p12.3	1.07	0.01855
LRRC8C	1p22.2	1.07	0.01288
SLC38A5	Xp11.23	1.07	0.04225
VCL	10q22.2	1.07	0.00310
NLRC4	2p22-p21	1.07	0.02430
BTG2	1q32	1.07	0.01602
KCND3	1p13.3	1.07	0.04824
CXCL16	17p13	1.07	0.01961
SLC29A1	6p21.1	1.07	0.00208
ZBTB10	8q13-q21.1	1.07	0.01272
PACSIN3	11p12-p11.12	1.07	0.00988
APOL1	22q13.1	1.07	0.01079
CKAP4	12q23.3	1.07	0.00759
LRRC32	11q13.5-q14	1.07	0.00309
RBPMS2	15q22.31	1.07	0.01437
CCRL1	NA	1.07	0.00513

TNFSF8	9q33	1.07	0.02191
YPEL4	11q12.1	1.08	0.03986
SCARF2	22q11.21	1.08	0.00872
CAMSAP2	1q32.1	1.08	0.00782
FZD4	11q14.2	1.08	0.00592
OSCP1	1p34.3	1.08	0.00528
FBXL7	5p15.1	1.08	0.00514
RRAGD	6q15-q16	1.08	0.00746
CSGALNACT2	10q11.21	1.09	0.03041
LAYN	11q23.1	1.09	0.00464
MAFB	20q11.2-q13.1	1.09	0.04776
FAM229B	6q21	1.09	0.00480
GPRC5B	16p12	1.09	0.02496
SLC1A4	2p15-p13	1.09	0.01753
SERPINF1	17p13.3	1.09	0.02935
SPG20	13q13.3	1.09	0.00513
BACE2	21q22.3	1.09	0.02174
ARNTL2	12p12.2-p11.2	1.10	0.01040
SLC47A1	17p11.2	1.10	0.00597
NRP1	10p12	1.10	0.01049
TRIB1	8q24.13	1.10	0.00261
RNF149	2q11.2	1.10	0.00748
ST3GAL3	1p34.1	1.11	0.00069
LYN	8q13	1.11	0.00933
SPOCD1	1p35.2	1.11	0.02031
MMP14	14q11.2	1.11	0.03945
RPS6KA2	6q27	1.11	0.00233
CD276	15q23-q24	1.11	0.00352
PIM3	22q13	1.11	0.00645
HIP1	7q11.23	1.11	0.02720
RNF145	5q33.3	1.11	0.00044
PCAT19	NA	1.11	0.01563
NXPE3	3q12.3	1.11	0.00265
SLAMF8	1q23.2	1.11	0.04569

SH3RF3	2q13	1.12	0.00189
RRAD	16q22	1.12	0.02281
HSPA2	14q24.1	1.12	0.00796
SYDE1	19p13.12	1.12	0.00783
GPC1	2q35-q37	1.12	0.00071
GYG2	Xp22.3	1.12	0.00463
PLEKHO1	1q21.2	1.12	0.01346
WWC2	4q35.1	1.12	0.04119
HSPA13	21q11	1.12	0.00933
EMCN	4q24	1.12	0.00446
MAB21L2	4q31	1.12	0.02795
SLC9B2	4q24	1.13	0.01443
PPP1R14A	19q13.1	1.13	0.03135
GRIK3	1p34.3	1.13	0.03204
ADGRA2	NA	1.13	0.03916
SMIM3	5q33.1	1.13	0.00106
PTGS1	9q32-q33.3	1.13	0.02230
PPP1R12B	1q32.1	1.14	0.02873
TCEAL3	Xq22.2	1.14	0.00542
MDM2	12q14.3-q15	1.14	0.00927
DNAJC18	5q31.2	1.14	0.00205
MCL1	1q21	1.14	0.00988
ERN1	17q24.2	1.14	0.03798
SLC24A3	20p13	1.14	0.01271
TCEAL9	NA	1.14	0.00036
TRPS1	8q24.12	1.14	0.02380
GRAMD1A	19q13.13	1.15	0.02809
GMPR	6p23	1.15	0.00137
CTSB	8p22	1.15	0.01319
FZD3	8p21	1.15	0.00115
RIPOR3	NA	1.15	0.01588
BMP6	6p24-p23	1.15	0.00599
GYPC	2q14-q21	1.15	0.03269
CCND2	12p13	1.15	0.02133

CSRP1	1q32	1.15	0.00686
NOCT	NA	1.15	0.04858
BNC2	9p22.2	1.15	0.01031
NFATC4	14q11.2	1.15	0.00599
MAP3K20	NA	1.15	0.00684
PODXL	7q32-q33	1.15	0.00185
MEIS2	15q14	1.15	0.01244
NID2	14q22.1	1.15	0.04974
EPAS1	2p21-p16	1.15	0.00525
LRFN4	11q13.2	1.15	0.01532
NLGN2	17p13.1	1.16	0.01264
PALLD	4q32.3	1.16	0.00166
HAVCR2	5q33.3	1.16	0.02950
CDK2AP1	12q24.31	1.16	0.00099
ZSWIM4	19p13.13	1.16	0.00412
PLCD3	17q21.31	1.16	0.03947
PODNL1	19p13.12	1.16	0.02771
C1QTNF7	4p15.3	1.17	0.03493
RAB3D	19p13.2	1.17	0.01782
PGM2L1	11q13.4	1.17	0.00649
CFH	1q32	1.17	0.01100
RIPK2	8q21	1.17	0.01287
CLEC1A	12p13.2	1.17	0.00891
SRPX	Xp21.1	1.17	0.01138
PEA15	1q21.1	1.17	0.01769
ITGA8	10p13	1.17	0.04984
FES	15q26.1	1.18	0.00115
ARAP3	5q31.3	1.18	0.00325
ARHGAP10	4q31.23	1.18	0.00262
SERPINE2	2q36.1	1.18	0.02841
SETD7	4q28	1.18	0.00663
SDCBP	8q12	1.18	0.00290
CLEC14A	14q21.1	1.19	0.01485
CDC42EP1	22q13.1	1.19	0.00203

GJD3	17q21.2	1.19	0.00323
ARL4C	2q37.1	1.19	0.00579
MTCL1	NA	1.19	0.00115
AHR	7p15	1.19	0.00700
CCPG1	15q21.1	1.19	0.00065
THSD7A	7p21.3	1.19	0.00852
ST6GALNAC2	17q25.1	1.20	0.00534
EOGT	3p14.1	1.20	0.00788
NOS3	7q36	1.20	0.03899
ADCY4	14q12	1.20	0.00701
B3GALNT1	3q25	1.20	0.00126
CYBA	16q24	1.20	0.02738
CD81	11p15.5	1.21	0.00315
RBPMS	8p12	1.21	0.00264
H1FX-AS1	3q21.3	1.21	0.00755
SPARCL1	4q22.1	1.21	0.03285
TNFRSF17	16p13.1	1.21	0.02942
EGFL7	9q34.3	1.21	0.02455
CLIP4	2p23.2	1.21	0.00442
KCTD12	13q22.3	1.21	0.00093
ETV5	3q28	1.21	0.00341
CDK14	7q21-q22	1.22	0.02232
STARD13	13q13.1	1.22	0.00921
F2R	5q13	1.22	0.00135
ST8SIA4	5q21	1.22	0.00721
NRSN2	20p13	1.22	0.00465
ARSB	5q14.1	1.22	0.00651
PSTPIP2	18q12	1.22	0.04349
ME1	6q12	1.22	0.00036
FARP1	13q32.2	1.22	0.00019
CX3CL1	16q13	1.22	0.00538
ABL2	1q25.2	1.22	0.00485
SPTBN5	15q21	1.22	0.01967
B9D1	17p11.2	1.22	0.01142

TRIP6	7q22	1.22	0.00017
PHLDA2	11p15.4	1.22	0.02790
ZMYND15	17p13.2	1.23	0.01601
USHBP1	19p13	1.23	0.00122
SLA	8q24	1.23	0.02305
AGTRAP	1p36.22	1.23	0.01217
ANXA1	9q21.13	1.23	0.00203
GNG11	7q21	1.23	0.00511
NAT14	19q13.42	1.23	0.01166
PODN	1p32.3	1.23	0.02908
CPM	12q14.3	1.23	0.03495
C3AR1	12p13.31	1.23	0.04715
GAS7	17p13.1	1.23	0.02156
GLT8D2	12q	1.23	0.00351
NTM	11q25	1.24	0.01897
AIF1	6p21.3	1.24	0.00195
EMP3	19q13.3	1.24	0.02431
DSEL	18q22.1	1.24	0.01346
NUCB2	11p15.1	1.24	0.00370
RCN1	11p13	1.25	0.00154
ODF3B	22q13.33	1.25	0.00816
VASN	16p13.3	1.25	0.01068
NLRC5	16q13	1.25	0.01399
KCNMA1	10q22.3	1.25	0.02563
COL5A3	19p13.2	1.25	0.02962
MAPK11	22q13.33	1.25	0.01459
STXBP1	9q34.1	1.25	0.00558
SDC2	8q22-q23	1.25	0.00498
ZCCHC24	10q22.3	1.25	0.00293
KCNE3	11q13.4	1.25	0.02057
IGDCC4	15q22.31	1.26	0.02958
TSHZ3	19q12	1.26	0.01240
ANXA5	4q27	1.26	0.00097
SLCO3A1	15q26	1.26	0.01256

SPATA18	4q12	1.26	0.00072
GNAI1	7q21	1.26	0.00139
PTK7	6p21.1-p12.2	1.26	0.00530
CRIP2	14q32.3	1.26	0.01675
B4GALT1	9p13	1.26	0.00098
PCDHGB7	5q31	1.26	0.01433
MTHFD1L	6q25.1	1.26	0.00199
ADGRE3	NA	1.27	0.01308
ARHGEF17	11q13.4	1.27	0.00319
MFAP2	1p36.1-p35	1.27	0.04703
ARID5A	2q11.2	1.27	0.01457
MS4A4A	11q12	1.27	0.01068
SHISA4	1q32.1	1.27	0.00101
LBH	2p23.1	1.27	0.00607
VIM	10p13	1.27	0.02056
CFD	19p13.3	1.27	0.01702
UBXN10	1p36.12	1.27	0.01410
A2M	12p13.31	1.27	0.01284
TRIM69	15q21.1	1.27	0.01014
ASPHD2	22q12.1	1.28	0.00219
DACT3	19q13.32	1.28	0.02537
GPR161	1q24.2	1.28	0.01970
SULF2	20q12-q13.2	1.28	0.02259
ARHGEF15	17p13.1	1.28	0.00921
SEMA3A	7p12.1	1.28	0.00440
CD109	6q13	1.28	0.01298
CFI	4q25	1.28	0.00229
CHST2	3q24	1.28	0.01281
BICD1	12p11.2-p11.1	1.28	0.00183
CCDC88A	2p16.1	1.28	0.00225
SPRY1	4q28.1	1.28	0.00547
TTC7B	14q32.11	1.28	0.00118
PLK3	1p34.1	1.28	0.04432
SULF1	8q13.1	1.28	0.01726

FAM212B	1p13.2	1.28	0.00114
TM4SF1	3q21-q25	1.28	0.01824
FRMD6	14q22.1	1.29	0.00770
PRDX4	Xp22.11	1.29	0.03388
LRRC4	7q31.3	1.29	0.02149
SMOX	20p13	1.29	0.00085
HEY1	8q21	1.29	0.00462
PDLIM4	5q31.1	1.29	0.01184
TSPAN2	1p13.2	1.29	0.00623
A4GALT	22q13.2	1.29	0.00674
CCM2L	20q11.2	1.29	0.00262
HERPUD1	16q13	1.29	0.00182
AMOTL2	3q21-q22	1.29	0.01041
CD34	1q32	1.29	0.00592
PLCB1	20p12	1.30	0.00450
FADS1	11q12.2-q13.1	1.30	0.02881
KCNE4	2q36.1	1.30	0.00606
CHST11	12q	1.30	0.02230
AMOTL1	11q14.3	1.30	0.00414
CADM1	11q23.2	1.30	0.00102
CAVIN2	NA	1.30	0.00047
RBMS2	12q13.3	1.30	0.00056
CYYR1	21q21.2	1.30	0.00125
ERG	21q22.3	1.30	0.00308
PRCP	11q14	1.30	0.00447
ADA2	NA	1.30	0.00842
FSCN1	7p22	1.30	0.01286
LZTS1	8p22	1.31	0.04253
MMRN2	10q23.2	1.31	0.00676
COL6A1	21q22.3	1.31	0.02448
ENAH	1q42.12	1.31	0.00130
DNAJB4	1p31.1	1.31	0.01131
RHOJ	14q23.2	1.31	0.00098
ADGRE1	NA	1.31	0.00763

MFGE8	15q25	1.31	0.01055
QKI	6q26	1.31	0.00717
SIGLEC9	19q13.41	1.31	0.03667
MAPK8IP1	11p11.2	1.32	0.00038
VEGFC	4q34.3	1.32	0.00590
MAGI2-AS3	NA	1.32	0.00077
NUPR1	16p11.2	1.32	0.00702
CPNE5	6p21.1	1.32	0.02415
GPT2	16q12.1	1.32	0.01044
SSPN	12p11.2	1.32	0.00233
C1ORF162	NA	1.32	0.00068
HEG1	3q21.2	1.32	0.00687
GDF15	19p13.11	1.32	0.01830
IKBIP	12q23.1	1.32	0.00227
LRRC25	19p13.11	1.32	0.00752
SLC38A2	12q	1.32	0.00151
MBNL1-AS1	3q25.1	1.33	0.00813
PDGFRB	5q33.1	1.33	0.01421
PDZRN3	3p13	1.33	0.00156
LGI4	19q13.11;19q13.12	1.33	0.01450
RAB34	17q11.2	1.33	0.00522
HSPA12A	10q26.12	1.33	0.00547
IL10RA	11q23	1.33	0.01561
PDK1	2q31.1	1.33	0.00282
SLAMF7	1q23.1-q24.1	1.33	0.02479
SCNN1B	16p12.2-p12.1	1.33	0.01247
SHANK3	22q13.3	1.33	0.00377
C4ORF32	NA	1.33	0.00218
MPDZ	9p23	1.33	0.00782
MECOM	3q26.2	1.34	0.00740
DPYSL3	5q32	1.34	0.01784
NUP50-AS1	NA	1.34	0.00092
LDB2	4p16	1.34	0.00157
PNMA2	8p21.2	1.34	0.01770

SEMA6B	19p13.3	1.34	0.00247
PTPN13	4q21.3	1.34	0.00428
ANOS1	NA	1.34	0.00814
PDE4D	5q12	1.34	0.00158
PDE7B	6q23-q24	1.35	0.00028
ZBP1	20q13.31	1.35	0.00352
TGFB1I1	16p11.2	1.35	0.00048
CARMN	NA	1.35	0.01765
NDUFA4L2	12q13.3	1.35	0.02235
NLRP3	1q44	1.35	0.00541
CD86	3q21	1.35	0.02668
BLVRA	7p13	1.35	0.00083
RUNX1	21q22.3	1.35	0.00080
MXRA7	17q25.1	1.35	0.00934
SH3TC1	4p16.1	1.35	0.00644
MSC-AS1	NA	1.35	0.01624
NOTCH4	6p21.3	1.35	0.00396
AOC3	17q21	1.36	0.02289
ADAM33	20p13	1.36	0.01126
CACNA1C	12p13.3	1.36	0.03026
FEZ1	11q24.2	1.36	0.00021
TCEAL7	Xq22.1	1.36	0.00074
OLFML3	1p13.2	1.36	0.00110
FHL1	Xq26	1.36	0.01128
TPM4	19p13.1	1.36	0.00062
ZEB2	2q22.3	1.36	0.00688
FILIP1	6q14.1	1.36	0.00344
C1QA	1p36.12	1.36	0.00403
SPI1	11p11.2	1.37	0.01781
PLEKHG2	19q13.2	1.37	0.00868
KLF6	10p15	1.37	0.00224
FAT4	4q28.1	1.37	0.00404
CCDC170	6q25.1	1.37	0.00875
CTLA4	2q33	1.37	0.03440

GLI3	7p13	1.37	0.00210
LAMA2	6q22-q23	1.38	0.00143
SDK1	7p22.2	1.38	0.03874
MEIS1	2p14	1.38	0.00667
EFEMP2	11q13.1	1.38	0.00075
TSPAN4	11p15.5	1.38	0.00214
RAMP2	17q12-q21.1	1.38	0.01134
TGFBR1	9q22	1.38	0.00132
ADGRL2	NA	1.38	0.00569
LY6E	8q24.3	1.38	0.01893
DENND5A	11p15.4	1.38	0.03509
CYBB	Xp21.1	1.38	0.03294
ACTN1	14q22-q24;14q24	1.38	0.00422
TESC	12q24.22	1.38	0.01246
F3	1p22-p21	1.38	0.00084
AGPAT4	6q26	1.39	0.00706
BCL6B	17p13.1	1.39	0.00645
LAMC1	1q31	1.39	0.00537
HSPA12B	20p13	1.39	0.00471
LST1	6p21.3	1.39	0.00115
EGFLAM	5p13.2-p13.1	1.40	0.01637
ABCC9	12p12.1	1.40	0.00456
PCDH7	4p15	1.40	0.02127
SLC36A4	11q21	1.40	0.00040
PHLDA3	1q31	1.40	0.00918
ERRFI1	1p36	1.40	0.02754
CHSY1	15q26.3	1.40	0.00909
NRN1	6p25.1	1.40	0.00497
FAM26E	6q22.1	1.40	0.00754
DENND2A	7q34	1.40	0.00032
XBP1	22q12;22q12.1	1.41	0.00491
TUBA1A	12q13.12	1.41	0.00282
FBLN5	14q32.1	1.41	0.00055
ENG	9q34.11	1.41	0.00305

PDE4A	19p13.2	1.41	0.00038
MEG3	14q32	1.41	0.00164
FOLR2	11q13.3-q13.5	1.41	0.00629
PLTP	20q13.12	1.41	0.00252
CMAHP	6p21.32	1.41	0.00134
CFP	Xp11.4	1.41	0.00953
PGF	14q24.3	1.42	0.03754
TIE1	1p34-p33	1.42	0.00562
ZNF385A	12q13.13	1.42	0.00120
CD300LB	17q25.1	1.42	0.00070
BEST1	11q13	1.42	0.01082
ABCA1	9q31.1	1.42	0.01767
SEC14L1	17q25.2	1.42	0.00104
SLC16A6	17q24.2	1.42	0.02414
FGR	1p36.2-p36.1	1.42	0.02549
ARMC9	2q37.1	1.42	0.00231
CNN2	19p13.3	1.42	0.00107
GNA14	9q21	1.42	0.00028
HYAL1	3p21.3-p21.2	1.42	0.00064
RAB11FIP5	2p13	1.43	0.00124
FAM198B	4q32.1	1.43	0.00224
ECM1	1q21	1.43	0.00149
ZNF618	9q32	1.43	0.00336
WTIP	19q13.11	1.43	0.00013
DOCK4	7q31.1	1.43	0.04224
POU2AF1	11q23.1	1.43	0.01586
C1ORF198	NA	1.43	0.00025
DUSP10	1q41	1.44	0.00107
DNAJB5	9p13.3	1.44	0.00313
MYC	8q24.21	1.44	0.00185
RUSC2	9p13.3	1.44	0.00223
NHSL2	Xq13.1	1.44	0.00391
CHST3	10q22.1	1.44	0.00588
RAB3B	1p32-p31	1.45	0.00803

GLDC	9p22	1.45	0.00054
COL6A2	21q22.3	1.45	0.00740
CALU	7q32.1	1.45	0.00106
ITGB2	21q22.3	1.45	0.03351
CHST15	10q26	1.45	0.00113
SOX18	20q13.33	1.45	0.01469
DZIP1L	3q22.3	1.45	0.00321
RDX	11q23	1.45	0.00376
GBP4	1p22.2	1.46	0.02712
NOD2	16q21	1.46	0.02623
S1PR1	1p21	1.46	0.00926
EMILIN1	2p23.3-p23.2	1.46	0.00018
DBN1	5q35.3	1.46	0.00072
SSR4	Xq28	1.46	0.02197
IGFBP4	17q12-q21.1	1.46	0.01732
ZYX	7q32	1.46	0.00019
TGFBI	5q31	1.46	0.00782
CEBPB	20q13.1	1.47	0.00675
MIR100HG	11q24.1	1.47	0.02915
ZNF521	18q11.2	1.47	0.00192
BICC1	10q21.1	1.47	0.03496
FGF2	4q26	1.47	0.00764
APOD	3q26.2-qter	1.47	0.00017
C2	6p21.3	1.48	0.00074
GEM	8q13-q21	1.48	0.02132
MTHFD2	2p13.1	1.48	0.00116
MAP1A	15q15.3	1.48	0.00043
UBTD1	10q24.2	1.48	0.00507
KLHDC7B	22q13.33	1.48	0.00494
EGR2	10q21.1	1.48	0.02722
C1QB	1p36.12	1.48	0.00257
NTN1	17p13-p12	1.48	0.04543
LITAF	16p13.13	1.48	0.00806
S100A4	1q21	1.48	0.00752

DAPP1	4q25-q27	1.48	0.03044
TRABD2B	1p33	1.48	0.01057
APOE	19q13.2	1.49	0.03196
PLXNA4	7q32.3	1.49	0.03215
PFKFB3	10p15.1	1.49	0.01977
CD59	11p13	1.49	0.00059
BOC	3q13.2	1.49	0.03963
LILRA1	19q13.4	1.49	0.01091
INMT	7p14.3	1.49	0.02685
ANK1	8p11.1	1.49	0.02198
LIMS2	2q14.3	1.49	0.01358
CDH5	16q22.1	1.49	0.00205
DUSP6	12q22-q23	1.49	0.00083
C1S	12p13	1.49	0.00723
AK1	9q34.1	1.50	0.01649
CREM	10p11.21	1.50	0.00122
ELN	7q11.23	1.50	0.01535
PLXND1	3q22.1	1.50	0.01270
PIK3R3	1p34.1	1.50	0.00003
EHD2	19q13.3	1.50	0.00112
GJA1	6q22.31	1.50	0.00321
TYMP	22q13.33	1.50	0.00793
ANTXR1	2p13.1	1.50	0.01112
NXN	17p13.3	1.50	0.00241
CFL2	14q12	1.50	0.00137
DYRK3	1q32.1	1.51	0.00721
STARD8	Xq13.1	1.51	0.01085
ANGPT2	8p23.1	1.51	0.01380
MPP3	17q21.31	1.51	0.01237
SCN1B	19q13.1	1.51	0.00395
MEIS3	19q13.32	1.51	0.00004
SH3PXD2B	5q35.1	1.51	0.02604
FMOD	1q32	1.51	0.00260
ESAM	11q24.2	1.52	0.00240

RARRES2	7q36.1	1.52	0.00905
RBMS1	2q24.2	1.52	0.00190
CTSL	9q21.33	1.52	0.01006
C8ORF4	NA	1.52	0.00175
TLR4	9q33.1	1.52	0.01562
VWA1	1p36.33	1.52	0.00147
MITF	3p14.2-p14.1	1.52	0.00247
SELENOM	NA	1.52	0.03684
PDGFB	22q13.1	1.53	0.00084
GZMH	14q11.2	1.53	0.00550
TP53INP1	8q22	1.53	0.00854
CXCR4	2q21	1.53	0.04125
KCNMB1	5q34	1.53	0.00144
CAV1	7q31.1	1.53	0.00164
APCDD1	18p11.22	1.53	0.02489
PDE1A	2q32.1	1.53	0.00565
PTGES	9q34.3	1.53	0.01153
RGCC	13q14.11	1.53	0.00366
MRAS	3q22.3	1.53	0.00726
SYT11	1q21.2	1.54	0.00131
JCHAIN	NA	1.54	0.00285
ARHGAP29	1p22.1	1.54	0.00154
ITGAX	16p11.2	1.54	0.04352
S100B	21q22.3	1.55	0.00004
EBF1	5q34	1.55	0.01610
APLNR	11q12	1.55	0.00146
FHL3	1p34	1.55	0.00023
PTPRU	1p35.3	1.55	0.02185
SFRP1	8p11.21	1.55	0.03113
COPZ2	17q21.32	1.55	0.00463
TYROBP	19q13.1	1.55	0.00235
RERG	12p12.3	1.56	0.00573
RASIP1	19q13.33	1.56	0.00312
FAM26F	6q22.1	1.56	0.02071

ICAM1	19p13.3-p13.2	1.56	0.03739
MSRB3	12q14.3	1.56	0.00416
TWSG1	18p11.3	1.56	0.00053
GNB4	3q26.33	1.56	0.00334
ADGRL4	NA	1.56	0.00551
BATF3	1q32.3	1.56	0.00215
MYH10	17p13	1.56	0.00349
CLMP	11q24.1	1.56	0.00914
RASGRP4	19q13.1	1.57	0.00887
NID1	1q43	1.57	0.00112
CLEC7A	12p13.2	1.57	0.01187
ANGPT1	8q23.1	1.57	0.03941
SOD3	4p15.2	1.57	0.03340
PRICKLE2	3p14.1	1.57	0.00101
QSOX1	1q24	1.57	0.00001
PHLDB2	3q13.2	1.57	0.00450
FADS2	11q12.2	1.58	0.00781
CCND1	11q13	1.58	0.00002
CCL8	17q11.2	1.58	0.01407
BTBD19	1p34.1	1.58	0.02902
OSMR	5p13.1	1.58	0.00727
FGFR1	8p12	1.58	0.00755
C1R	12p13	1.58	0.00704
TMEM170B	6p24.2	1.58	0.00071
IGFBP7	4q12	1.58	0.00059
ADAMTS12	5q35	1.58	0.00961
PXDN	2p25	1.59	0.00423
FNDC3B	3q26.31	1.59	0.00194
FAM46C	1p12	1.59	0.00254
ST3GAL4	11q24.2	1.60	0.00803
BST2	19p13.1	1.60	0.00107
INHBB	2cen-q13	1.60	0.00432
SNX10	7p15.2	1.60	0.04800
TMEM47	Xp11.4	1.60	0.00223

ITPRIP	10q25.1	1.60	0.00556
SERPING1	11q12.1	1.60	0.01570
CALCRL	2q32.1	1.60	0.00716
PTPN14	1q32.2	1.60	0.00082
TPST1	7q11.21	1.60	0.00117
ELL2	5q15	1.61	0.00127
CEP112	17q24.1	1.61	0.00005
C16ORF45	NA	1.61	0.00019
SLC17A9	20q13.33	1.61	0.00120
FKBP11	12q13.12	1.61	0.00379
SPON2	4p16.3	1.61	0.00064
NECTIN4	NA	1.62	0.00900
LMOD1	1q32	1.62	0.01377
MYCT1	6q25.2	1.62	0.00018
TIMP2	17q25	1.62	0.00635
PLS3	Xq23	1.62	0.00008
IRAK3	12q14.3	1.62	0.00680
TNS1	2q35-q36	1.62	0.01242
GPR176	15q14-q15.1	1.62	0.03690
AKAP12	6q24-q25	1.62	0.00229
TSPAN18	11p11.2	1.63	0.00027
LGALS1	22q13.1	1.63	0.00641
DKK3	11p15.2	1.63	0.00046
ANKRD36BP2	2p11.2	1.63	0.00143
CCR1	3p21	1.63	0.02272
GLUL	1q31	1.63	0.00740
IFI30	19p13.1	1.63	0.00231
RAB23	6p11	1.64	0.00471
SEMA4A	1q22	1.64	0.01337
ARHGAP28	18p11.31	1.64	0.00037
GPX8	5q11.2	1.64	0.00063
FAM167B	1p35.1	1.64	0.00463
TUBB6	18p11.21	1.64	0.00087
FNDC4	2p23.3	1.64	0.00480

LRCH2	Xq23	1.64	0.00295
CH25H	10q23	1.64	0.00574
NAP1L5	4q21-q22;4q22.1	1.64	0.00001
SRPX2	Xq21.33-q23	1.64	0.00410
CDH11	16q21	1.65	0.00256
ZBTB47	3p22.1	1.65	0.00048
LOXL2	8p21.3	1.65	0.00565
APOC1	19q13.2	1.65	0.00993
COL4A2	13q34	1.65	0.00185
IL33	9p24.1	1.65	0.00537
ADGRE2	NA	1.65	0.00643
FKBP10	17q21.2	1.66	0.00272
LAX1	1q32.1	1.66	0.00759
ITGA11	15q23	1.66	0.00352
PTAFR	1p35-p34.3	1.66	0.01381
C19ORF38	NA	1.66	0.00101
DUSP1	5q34	1.66	0.00584
DRAM1	12q23.2	1.66	0.00218
CYTOR	NA	1.67	0.01627
SDC3	1pter-p22.3	1.67	0.00031
MRC1	10p12.33	1.67	0.00140
NFAM1	22q13.2	1.67	0.01522
MS4A7	11q12	1.68	0.00138
ADAMTSL3	15q25.2	1.68	0.04749
CD93	20p11.21	1.68	0.00251
ASPN	9q22	1.68	0.00940
MRVI1	11p15	1.68	0.00568
TSHZ2	20q13.2	1.68	0.00069
GRASP	12q13.13	1.68	0.00015
IRS1	2q36	1.68	0.00016
CSGALNACT1	8p21.3	1.69	0.01356
ADAMTS5	21q21.3	1.69	0.00142
UBE2E2	3p24.2	1.69	0.00063
SSC5D	19q13.42	1.69	0.00208

HMCN1	1q25.3-q31.1	1.69	0.00626
STEAP1	7q21	1.69	0.01400
LHX6	9q33.2	1.69	0.00494
RAB31	18p11.3	1.70	0.00611
CSTA	3q21	1.70	0.00259
FAM49A	2p24.2	1.70	0.00178
LOC729737	1p36.33	1.70	0.03929
HEYL	1p34.3	1.71	0.00162
S1PR3	9q22.1-q22.2	1.71	0.03050
HSPB8	12q24.23	1.71	0.01875
PRKD1	14q11	1.71	0.00170
S100A11	1q21	1.71	0.00034
DLC1	8p22	1.72	0.00128
KCNAB1	3q26.1	1.72	0.00978
CCL13	17q11.2	1.72	0.00566
GPNMB	7p15	1.72	0.01272
RASSF8	12p12.3	1.73	0.01407
FSTL3	19p13	1.73	0.00184
SGCD	5q33-q34	1.73	0.00125
TMEM255B	13q34	1.73	0.00103
CALD1	7q33	1.73	0.00057
ACSL4	Xq22.3-q23	1.73	0.00535
FAM124A	13q14.3	1.73	0.00787
NPL	1q25	1.73	0.00179
WWTR1	3q23-q24	1.74	0.00015
MXRA5	Xp22.33	1.74	0.01266
GZMB	14q11.2	1.74	0.03770
VLDLR	9p24	1.74	0.00530
PRSS23	11q14.1	1.74	0.00102
DUSP14	17q12	1.74	0.00220
PTGER3	1p31.2	1.74	0.01345
FBLN1	22q13.31	1.74	0.00103
GBP1P1	1p22.2	1.75	0.03002
LYZ	12q15	1.75	0.00858

ECSCR	5q31.2	1.75	0.00463
GJA4	1p35.1	1.75	0.00130
LAMA4	6q21	1.75	0.00009
PLAU	10q22.2	1.76	0.00259
BASP1	5p15.1	1.76	0.00820
MRC2	17q23.2	1.76	0.00116
RAMP3	7p13-p12	1.76	0.00579
LSAMP	3q13.2-q21	1.76	0.02740
ROBO4	11q24.2	1.76	0.00014
COL15A1	9q21-q22	1.76	0.00340
DPT	1q12-q23	1.77	0.02358
MMRN1	4q22	1.77	0.00089
CLEC12A	12p13.2	1.77	0.00208
BAG2	6p12.1-p11.2	1.77	0.00194
CDH13	16q23.3	1.77	0.00289
CPZ	4p16.1	1.77	0.00271
COL5A1	9q34.2-q34.3	1.78	0.00403
CLIC4	1p36.11	1.78	0.00152
FERMT2	14q22.1	1.78	0.00018
MAFF	22q13.1	1.78	0.00697
CXORF36	NA	1.78	0.00041
RNF144B	6p22.3	1.79	0.00462
DDIT4	10q22.1	1.79	0.00106
THBD	20p11.2	1.79	0.00478
TMTC1	12p11.22	1.79	0.00005
STON1	2p16.3	1.79	0.00104
FOXP3	Xp11.23	1.79	0.00958
CREB5	7p15.1	1.79	0.02166
ARID5B	10q21.2	1.79	0.00023
GREB1	2p25.1	1.80	0.00643
RTL8B	NA	1.80	0.00046
LOC283710	15q13.3	1.81	0.00507
PAPPA	9q33.2	1.81	0.00299
CHN1	2q31.1	1.82	0.01270

ACSL1	4q35	1.82	0.02825
STAT1	2q32.2	1.83	0.00412
LHFP	13q12	1.83	0.00015
FLNA	Xq28	1.83	0.00241
LY96	8q21.11	1.84	0.00371
JCAD	NA	1.84	0.00092
GJC1	17q21.31	1.85	0.00181
LRRN4CL	11q12.3	1.85	0.00080
IFITM3	11p15.5	1.85	0.00131
TLR8	Xp22	1.85	0.01991
MEOX1	17q21	1.85	0.00090
TMEM45A	3q12.2	1.86	0.00425
RGS5	1q23.1	1.86	0.00020
COL5A2	2q14-q32	1.86	0.00532
ADAMTS9	3p14.1	1.86	0.00453
SGIP1	1p31.3	1.86	0.00030
EGR3	8p23-p21	1.86	0.02079
C11ORF96	NA	1.87	0.00043
GUCY1A3	4q31.1-q31.2	1.87	0.01065
CCDC3	10p13	1.87	0.00281
TIMP3	22q12.3	1.88	0.00044
CD163	12p13.3	1.88	0.01361
POSTN	13q13.3	1.88	0.02653
MEI1	22q13.2	1.88	0.00149
MYOF	10q24	1.88	0.00015
PSAT1	9q21.2	1.88	0.00211
FAM129A	1q25	1.88	0.00437
NRP2	2q33.3	1.89	0.00131
PHGDH	1p12	1.89	0.00177
PRR16	5q23.1	1.89	0.00345
ARHGAP23	17q12	1.89	0.00168
HTRA3	4p16.1	1.90	0.00404
TMEM132A	11q12.2	1.90	0.02723
LILRB4	19q13.4	1.91	0.02783

ADAMTSL4	1q21.3	1.91	0.00254
CLIC6	21q22.12	1.91	0.00014
PDCD1LG2	9p24.2	1.91	0.01526
MAP1B	5q13	1.91	0.00022
MYL9	20q11.23	1.92	0.00185
GPC3	Xq26.1	1.92	0.00373
F5	1q23	1.92	0.00673
FLRT2	14q24-q32	1.92	0.00987
ACKR1	NA	1.93	0.00621
CCBE1	18q21.32	1.93	0.00043
ADAMTS6	5q12	1.93	0.00459
ADAMTS2	5qter	1.93	0.02108
MCAM	11q23.3	1.93	0.00403
EREG	4q13.3	1.93	0.02081
PLA2G7	6p21.2-p12	1.93	0.00277
VNN2	6q23-q24	1.94	0.03922
GNA15	19p13.3	1.94	0.00104
FN1	2q34	1.94	0.00779
FST	5q11.2	1.94	0.00739
TIMP1	Xp11.3-p11.23	1.94	0.00042
LMCD1	3p26-p24	1.95	0.00098
GJA5	1q21.1	1.95	0.00047
FBN1	15q21.1	1.95	0.00200
HSPA6	1q23	1.95	0.00685
TNFSF13B	13q32-q34	1.95	0.00193
CD38	4p15	1.95	0.00056
CLC	19q13.1	1.96	0.02684
F13A1	6p25.3-p24.3	1.96	0.00966
SPHK1	17q25.2	1.96	0.00532
CCL3	17q12	1.96	0.01506
PECAM1	17q23.3	1.96	0.00058
SPARC	5q31.3-q32	1.97	0.00229
HTRA1	10q26.3	1.98	0.00015
KLF2	19p13.11	1.98	0.00304

FOS	14q24.3	1.99	0.00472
EMILIN2	18p11.3	1.99	0.00060
SLC1A3	5p13	2.00	0.04137
GUCY1B3	4q31.3-q33	2.00	0.00429
MYADM	19q13.42	2.00	0.00028
KCNN3	1q21.3	2.00	0.00086
IFITM2	11p15.5	2.00	0.00192
PILRA	7q22.1	2.01	0.00946
DERL3	22q11.23	2.01	0.00183
ALDH1L2	12q23.3	2.01	0.00461
CNN1	19p13.2-p13.1	2.02	0.00276
CDH6	5p13.3	2.02	0.00008
VCAN	5q14.3	2.02	0.00211
GPR4	19q13.3	2.03	0.00593
HBEGF	5q23	2.03	0.01973
CAVIN1	NA	2.03	0.00014
COL12A1	6q12-q13	2.03	0.00399
CD14	5q31.1	2.03	0.00373
CAVIN3	NA	2.04	0.00186
P2RY14	3q24-q25.1	2.04	0.00133
CXCL11	4q21.2	2.04	0.03817
FCER1G	1q23	2.05	0.00404
EDNRA	4q31.22	2.05	0.00108
DES	2q35	2.05	0.04005
FAM110B	8q12.1	2.05	0.00019
ACTG2	2p13.1	2.05	0.00834
EFEMP1	2p16	2.06	0.00033
SELP	1q22-q25	2.07	0.00467
LOC284454	19p13.13	2.07	0.00058
NCF2	1q25	2.08	0.00360
FLNC	7q32-q35	2.08	0.01013
PDLIM7	5q35.3	2.08	0.00025
IL1R2	2q12	2.08	0.02937
P3H2	NA	2.09	0.00045

COL18A1	21q22.3	2.09	0.00004
MNDA	1q22	2.09	0.00736
CPXM1	20p13	2.09	0.00576
COL3A1	2q31	2.10	0.00999
IDO1	8p12-p11	2.10	0.01539
OLFML2B	1q23.3	2.10	0.01056
MN1	22q12.1	2.10	0.00074
KCNJ8	12p11.23	2.11	0.00033
LATS2	13q11-q12	2.11	0.00040
FAM20A	17q24.2	2.11	0.02094
GBP1	1p22.2	2.12	0.00641
WARS	14q32.31	2.12	0.00136
ITGA5	12q11-q13	2.12	0.00055
LUM	12q21.3-q22	2.13	0.00040
MSLN	16p13.3	2.13	0.04134
AKR1C1	10p15-p14	2.14	0.00464
AEBP1	7p13	2.14	0.00297
TAGLN	11q23.2	2.14	0.00049
GPC6	13q32	2.14	0.00002
PYGL	14q21-q22	2.15	0.00032
ROR2	9q22	2.15	0.00044
GRIN2D	19q13.33	2.16	0.00073
VSIG2	11q24	2.16	0.03300
COL7A1	3p21.1	2.16	0.00718
KYNU	2q22.2	2.17	0.00775
LPL	8p22	2.17	0.00066
SLC2A3	12p13.3	2.17	0.00568
SRGN	10q22.1	2.17	0.00070
FILIP1L	3q12.1	2.17	0.00219
ATF3	1q32.3	2.18	0.00097
TPM2	9p13	2.18	0.00073
ADM	11p15.4	2.18	0.00334
MIR4435-2HG	NA	2.19	0.00190
RHOB	2p24	2.20	0.00006

NOTCH3	19p13.2-p13.1	2.20	0.00185
HGF	7q21.1	2.21	0.00025
SVEP1	9q32	2.21	0.00017
BGN	Xq28	2.21	0.00133
CRISPLD2	16q24.1	2.22	0.00034
CERCAM	9q34.11	2.22	0.00045
HK3	5q35.2	2.22	0.00098
PER1	17p13.1	2.22	0.00003
PLN	6q22.1	2.23	0.00865
NEXN	1p31.1	2.23	0.00000
TSC22D3	Xq22.3	2.24	0.00042
DCN	12q21.33	2.24	0.00179
MFAP5	12p13.1-p12.3	2.24	0.00358
DYSF	2p13.3	2.25	0.00203
F2RL2	5q13	2.25	0.00003
PRDM1	6q21	2.27	0.00041
PDE4B	1p31	2.28	0.00213
PLAUR	19q13	2.28	0.00164
FBLN2	3p25.1	2.28	0.01719
MZB1	5q31.2	2.28	0.00352
PHLDA1	12q15	2.29	0.00013
ANO1	11q13.3	2.30	0.00057
BHLHE22	8q13	2.30	0.00117
CTSK	1q21	2.31	0.00130
CLDN1	3q28-q29	2.31	0.00941
ACTA2	10q23.3	2.31	0.00025
OSCAR	19q13.42	2.31	0.00274
VWF	12p13.3	2.32	0.00066
BHMT2	5q13	2.32	0.00011
WNT5A	3p21-p14	2.33	0.00268
STX11	6q24.2	2.33	0.00062
HSPB6	19q13.12	2.33	0.00716
SLC7A11	4q28-q32	2.33	0.00015
HAPLN3	15q26.1	2.34	0.01430

MMP2	16q13-q21	2.34	0.00198
FSTL1	3q13.33	2.34	0.00012
S100P	4p16	2.34	0.03514
COL4A1	13q34	2.35	0.00063
PLA1A	3q13.13-q13.2	2.35	0.00163
ADGRG3	NA	2.36	0.00036
COL6A3	2q37	2.36	0.00021
PRRX1	1q24	2.37	0.03388
PTGS2	1q25.2-q25.3	2.37	0.00341
CXCL2	4q21	2.37	0.02221
PCDH17	13q21.1	2.38	0.00171
H19	11p15.5	2.40	0.00537
PDE10A	6q26	2.41	0.00003
ANKRD22	10q23.31	2.41	0.00058
COL1A2	7q22.1	2.42	0.00108
LAMC3	9q31-q34	2.43	0.01525
ANGPTL2	9q34	2.43	0.00067
PTGDS	9q34.2-q34.3	2.43	0.00410
IL1RN	2q14.2	2.44	0.00994
FGF7	15q21.2	2.44	0.00203
SAA1	11p15.1	2.45	0.03303
PALMD	1p22-p21	2.45	0.00110
MGP	12p12.3	2.46	0.00024
MSR1	8p22	2.47	0.00027
CD248	11q13	2.47	0.00045
CYP7B1	8q21.3	2.48	0.00256
MRGPRF	11q13.3	2.48	0.00003
AOX1	2q33	2.49	0.00650
SDS	12q24.13	2.49	0.00030
SPAG4	20q11.21	2.50	0.00032
STEAP4	7q21.12	2.50	0.00958
CCL2	17q11.2-q12	2.51	0.00158
CCDC80	3q13.2	2.52	0.00104
THEMIS2	1p35.3	2.52	0.00005

LILRB2	19q13.4	2.53	0.00117
LINC00312	3p25.3	2.53	0.00076
CCL11	17q12	2.53	0.00154
ALPL	1p36.12	2.55	0.00296
SOCS3	17q25.3	2.55	0.02219
GAS1	9q21.3-q22	2.55	0.04265
ARSJ	4q26	2.56	0.00014
RGS16	1q25-q31	2.57	0.00418
CD55	1q32	2.57	0.00037
SLC7A5	16q24.3	2.57	0.00029
NNMT	11q23.1	2.59	0.00067
MEFV	16p13.3	2.60	0.00678
CXCL3	4q21	2.61	0.01430
RSPO3	6q22.33	2.62	0.00858
SYNPO2	4q26	2.63	0.00025
MEDAG	13q12.3	2.63	0.00175
CHAC1	15q15.1	2.63	0.00572
FMO3	1q24.3	2.63	0.00061
WISP1	8q24.22	2.64	0.00056
PDPN	1p36.21	2.64	0.00011
OGN	9q22	2.64	0.00725
IER3	6p21.3	2.64	0.00006
GBP5	1p22.2	2.68	0.01821
VSIG1	Xq22.3	2.68	0.01523
SPP1	4q22.1	2.69	0.01270
APOLD1	12p13.1	2.69	0.00035
MMP19	12q14	2.69	0.00117
MIR650	22q11.22	2.69	0.00067
BCAT1	12p12.1	2.70	0.00061
THY1	11q23.3	2.70	0.00017
CXCL9	4q21	2.70	0.03948
SIK1	21q22.3	2.71	0.00093
COL1A1	17q21.33	2.72	0.00119
BMS1P20	NA	2.74	0.00067

FCGR2A	1q23	2.74	0.00156
IGFBP5	2q33-q36	2.75	0.00030
LILRB3	19q13.4	2.75	0.00178
STC2	5q35.1	2.75	0.00249
CTHRC1	8q22.3	2.80	0.00091
COL14A1	8q23	2.83	0.00019
HBB	11p15.5	2.91	0.00030
DUSP4	8p12-p11	2.93	0.00006
C5AR1	19q13.3-q13.4	2.94	0.00065
MYEOV	11q13	2.94	0.00246
EGR1	5q31.1	2.96	0.00018
TGFB2	1q41	2.97	0.00074
LILRA6	19q13.4	2.97	0.00247
HBA2	16p13.3	2.98	0.00662
PDZRN4	12q12	3.02	0.00093
CSF3R	1p35-p34.3	3.03	0.00519
CXCR2	2q35	3.04	0.00680
PIM2	Xp11.23	3.08	0.00011
MUC1	1q21	3.09	0.00613
IGLL5	22q11.22	3.10	0.00004
NR4A1	12q13	3.14	0.00019
STC1	8p21-p11.2	3.16	0.00051
TMEM158	3p21.3	3.20	0.00097
NR4A2	2q22-q23	3.21	0.00006
IL1B	2q14	3.22	0.00400
CLDN18	3q22.3	3.26	0.03657
COL8A1	3q12.3	3.29	0.00532
SHC3	9q22.1	3.29	0.00126
INHBA	7p15-p13	3.33	0.00144
FIBIN	11p14.2	3.36	0.00000
TFPI2	7q22	3.37	0.00022
SLPI	20q12	3.40	0.00367
LILRA5	19q13.4	3.40	0.00023
FOXQ1	6p25	3.50	0.00367

SLC11A1	2q35	3.52	0.00319
CLEC4E	12p13.31	3.53	0.00171
ADAMTS1	21q21.2	3.53	0.00000
VIP	6q25	3.56	0.01058
CA2	8q22	3.58	0.00022
FAP	2q23	3.60	0.00961
RGS1	1q31	3.61	0.00002
MMP1	11q22.3	3.63	0.00219
CXCL1	4q21	3.63	0.00064
ADAMTS4	1q21-q23	3.65	0.00333
DUOX2	15q15.3	3.66	0.04276
THBS2	6q27	3.67	0.00078
CD300E	17q25.1	3.71	0.00368
CXCL6	4q13.3	3.75	0.00031
THBS1	15q15	3.75	0.00006
FCN1	9q34	3.78	0.00007
MMP3	11q22.3	3.82	0.01510
VGLL3	3p12.1	3.83	0.00021
S100A9	1q21	3.93	0.00145
KCNJ15	21q22.2	3.94	0.00519
FCGR3A	1q23	3.96	0.00006
GREM1	15q13.3	4.10	0.00320
CHI3L1	1q32.1	4.16	0.00470
NR4A3	9q22	4.17	0.00006
TNFAIP6	2q23.3	4.21	0.00369
FPR1	19q13.4	4.27	0.00014
CXCL5	4q13.3	4.28	0.00839
FCGR1A	1q21.2-q21.3	4.37	0.00008
S100A8	1q21	4.40	0.00056
TREM1	6p21.1	4.40	0.00023
FPR2	19q13.3-q13.4	4.46	0.00048
CHRDL2	11q14	4.52	0.00018
FCGR3B	1q23	4.54	0.00188
CTGF	6q23.1	4.57	0.00000

SERPINE1	7q22.1	4.83	0.00001
CXCR1	2q35	4.87	0.00136
FOLH1	11p11.2	4.88	0.00025
FOSB	19q13.32	4.94	0.00008
CXCL8	NA	5.29	0.00042
AQP9	15q	5.30	0.00101
SFRP2	4q31.3	5.53	0.00007
CYR61	1p22.3	5.62	0.00000
EGFL6	Xp22	5.62	0.00016
MUC6	11p15.5	5.84	0.00340
PGC	6p21.1	6.89	0.02007
